# Tracking gut microbiome and bloodstream infection in critically ill adults

**DOI:** 10.1371/journal.pone.0289923

**Published:** 2023-10-10

**Authors:** Christopher H. Gu, Layla A. Khatib, Ayannah S. Fitzgerald, Jevon Graham-Wooten, Caroline A. Ittner, Scott Sherrill-Mix, YuChung Chuang, Laurel J. Glaser, Nuala J. Meyer, Frederic D. Bushman, Ronald G. Collman

**Affiliations:** 1 Department of Microbiology, University of Pennsylvania School of Medicine, Philadelphia, PA, United States of America; 2 Department of Medicine, Pulmonary and Critical Care Division and the Center for Translational Lung Biology / Lung Biology Institute, University of Pennsylvania School of Medicine, Philadelphia, PA, United States of America; 3 Department of Pathology and Laboratory Medicine, University of Pennsylvania School of Medicine, Philadelphia, PA, United States of America; UAE University: United Arab Emirates University, UNITED ARAB EMIRATES

## Abstract

**Background:**

The gut microbiome is believed to contribute to bloodstream infection (BSI) via translocation of dominant gut bacteria in vulnerable patient populations. However, conclusively linking gut and blood organisms requires stringent approaches to establish strain-level identity.

**Methods:**

We enrolled a convenience cohort of critically ill patients and investigated 86 bloodstream infection episodes that occurred in 57 patients. Shotgun metagenomic sequencing was used to define constituents of their gut microbiomes, and whole genome sequencing and assembly was done on 23 unique bloodstream isolates that were available from 21 patients. Whole genome sequences were downloaded from public databases and used to establish sequence-identity distribution and define thresholds for unrelated genomes of BSI species. Gut microbiome reads were then aligned to whole genome sequences of the cognate bloodstream isolate and unrelated database isolates to assess identity.

**Results:**

Gut microbiome constituents matching the bloodstream infection species were present in half of BSI episodes, and represented >30% relative abundance of gut sequences in 10% of episodes. Among the 23 unique bloodstream organisms that were available for whole genome sequencing, 14 were present in gut at the species level. Sequence alignment applying defined thresholds for identity revealed that 6 met criteria for identical strains in blood and gut, but 8 did not. Sequence identity between BSI isolates and gut microbiome reads was more likely when the species was present at higher relative abundance in gut.

**Conclusion:**

In assessing potential gut source for BSI, stringent sequence-based approaches are essential to determine if organisms responsible for BSI are identical to those in gut: of 14 evaluable patients in which the same species was present in both sites, they were identical in 6/14, but were non-identical in 8/14 and thus inconsistent with gut source. This report demonstrates application of sequencing as a key tool to investigate infection tracking within patients.

## Introduction

Bloodstream infections (BSI) are a major cause of morbidity and mortality in hospitalized patients. BSIs often occur in vulnerable populations such as critically ill and immunocompromised patients and arise from multiple potential sources such as focal organ infection (pneumonia, urinary tract infection), indwelling vascular catheters, or other known infection sites. BSI are considered primary infections when no source is evident, which occurs in 10–20% of BSI episodes [1–4]. Rapid antibiotic treatment targeting the BSI organism is essential for optimal outcomes [5–7]; thus early identification and/or prediction of BSI organisms is critical.

Increasing interest has focused on translocation of microbes from the gut microbiome as a source of BSI [8–10]. Several studies reported organisms in stool being closely related to BSI organisms in hematopoietic stem cell transplant (HSCT) patients, who are extremely vulnerable to BSI due to combined immunosuppression and damaged mucosa from chemotherapy [11–15]. In these patients, BSI is typically associated with dominance of the gut microbiome by those organisms. The CDC has introduced the term mucosal barrier injury-associated, laboratory-confirmed bloodstream infection (MBI-LCBI) in surveillance of hematologic cancer patients [16–19]. MBI-LCBI are associated with significantly worse outcomes after HSCT [20]. Critically ill patients are another vulnerable population in which BSI are frequent. Critically ill patients often have dysbiotic gut microbiomes, frequently dominated by single genera [21–24], which is associated with poorer clinical outcomes [25–27]. Critically ill patients also show evidence of increased intestinal permeability suggesting mucosal barrier dysfunction [28–32], which might enable opportunistic translocation of gut microbiota through the mucosal barrier. However, there have been no studies relating BSI and gut microbiome in critically ill patients to date. Thus, our study investigated the relationship between BSI and the gut microbiome in a critically ill patient population.

To address the relatedness between gut microbiome and BSI organisms in critically ill patients, we collected stool and blood cultures from patients in a medical intensive care unit (ICU). A total of 86 BSI episodes that occurred in 57 patients were investigated. For each of the identified BSI species, we generated a matrix of whole genome sequences downloaded from public databases, curated to eliminate related or duplicated genomes, and established empiric sequence variance thresholds to distinguish whether BSI and gut sequences reflect the same organism. We performed shotgun metagenomic sequencing on stool to characterize the gut microbiome constituents, and whole genome sequencing (WGS) with de novo assembly on available blood culture isolates. By aligning stool reads to BSI whole genome sequences and applying a stringent single nucleotide variants per megabase pair (SNV/Mbp) threshold, we found that even when a BSI species was present within the gut microbiome, they were often distinct strains. Our study provides insight on the relationship between BSI and gut microbiome organisms in a novel population, critically ill patients, describes a method of establishing identity thresholds, and reveals that more than half of patients with the same species represented in blood and gut, the strains were non-identical.

## Methods

### Subjects and specimens

Patients admitted to the medical ICU of the Hospital of the University of Pennsylvania were enrolled prospectively and only those for whom stool was available were included. Enrollment criteria were age ≥18 years and expected ICU stay of >72 hours or clinical suspicion of sepsis. To capture additional BSI episodes, some patients were enrolled at the time of positive blood culture. Patients or surrogates provided informed consent under IRB-approved protocol #823392. Stool was obtained from spontaneous movements or fecal management system containers and stored (-80°C) until sequenced. BSI were identified by blood culture, which were collected, processed and the organisms identified per routine clinical practice. Blood cultures that were positive for *Staphylococcus epidermidis* were not included in our analysis due to their frequent appearance as contaminants, and patients with only *S*. *epidermidis* were considered as BSI-negative subjects. Blood culture isolates were identified by the clinical microbiology lab at the species level except for an *Achromobacter* BSI that was identified only at the genus level [33,34] and analyzed here as such. BSI isolates were frozen (-80°C) until sequenced. Clinical data were extracted from the electronic medical record (**Tables 1** and **S1**). Healthy stool microbiome profiles were from the Human Microbiome Project [35]. Further details are in Supplemental Methods.

**Table 1:** Clinical characteristics of enrolled patients. Major diagnosis refers to most common principal acute and underlying reasons for ICU stay; full details are in **S1 Table**. Numbers add to greater than 100% due to concomitant processes. There are no significant differences in any clinical characteristics between BSI-positive subjects and BSI-negative subjects or between BSI-positive subjects and BC-available subjects (chi-square test if categorical, t-test if continuous; applying 0.05 significance threshold).

	Total cohort subjects (N = 139)	BSI positive subjects (N = 57)	BSI negative subjects (N = 82)	BC available subjects (N = 21)
AGE (mean, range)	58.7 [20.0–99.0]	57.9 [22.0–99.0]	59.3 [20.0–91.0]	61 [22.0–99.0]
GENDER				
Female	64 (46.0%)	27 (47.4%)	37 (45.1%)	11 (52.4%)
Male	75 (54.0%)	30 (52.6%)	45 (54.9%)	10 (47.6%)
RACE				
White	67 (48.2%)	28 (49.1%)	39 (47.6%)	6 (28.6%)
Black	53 (38.1%)	24 (42.1%)	29 (35.4%)	13 (61.9%)
Asian	3 (2.2%)	1 (1.8%)	2 (2.4%)	1 (4.8%)
Unknown	16 (11.5%)	4 (7.1%)	12 (14.6%)	1 (4.8%)
MAJOR DIAGNOSIS / COMORBIDITY				
COVID-19	19 (13.7%)	6 (10.5%)	13 (15.9%)	3 (14.3%)
Hematologic malignancy	24 (17.3%)	14 (24.6%)	10 (12.2%)	5 (23.8%)
Solid tumor	27 (19.4%)	11 (19.3%)	16 (19.5%)	3 (14.3%)
Cirrhosis	22 (15.8%)	10 (17.5%)	12 (14.6%)	1 (4.8%)
Gastrointestinal Bleeding	15 (10.8%)	5 (8.8%)	10 (12.2%)	0 (0%)
Renal Failure	111 (79.9%)	48 (84.2%)	63 (76.8%)	17 (81.0%)
Neutropenia	9 (6.5%)	5 (8.8%)	4 (4.9%)	3 (14.3%)
Shock	111 (79.9%)	50 (87.7%)	61 (74.4%)	19 (90.5%)
**Blood cultures (genus)**	**Total BC+ episodes (N = 86)**			**(N = 23)**
*Achromobacter*	1			1
*Acinetobacter*	1			-
*Aerococcus*	1			-
*Bacteroides*	3			1
*Candida*	12			1
*Corynebacterium*	1			-
*Enterobacter*	3			-
*Enterococcus*	11			5
*Escherichia*	7			2
*Gemella*	1			-
*Klebsiella*	8			3
*Lactobacillus*	1			-
*Morganella*	2			1
*Mycobacterium*	1			-
*Proteus*	2			1
*Pseudomonas*	8			3
*Serratia*	2			1
*Staphylococcus (non epidermidis)*	14			3
*Stenotrophomonas*	1			-
*Streptococcus*	6			1

### Metagenomic sequencing

DNA was extracted from stool samples using the DNeasy PowerSoil Kit. Libraries for Illumina sequencing were prepared using the NexteraXT DNA Library Preparation Kit with Illumina’s indexes. Sequencing was performed on the NovaSeq6000 or HiSeq2500 using 2x150bp or 2x125bp chemistry, respectively. Blood culture isolates were subcultured on blood agar plates, colonies inoculated into Blood Heart Infusion Broth, and grown overnight at 37°C. DNA was extracted and libraries prepared as above. Sequencing was performed on the MiSeq platform with 2x250bp or 2x300bp chemistry.

### Gut microbiome analysis and BSI whole genome assembly

Stool samples (**S2 Table**) were demultiplexed using DNAbc [36]. Sequencing reads were processed and taxonomically classified using the Sunbeam pipeline v3.0.0 [37]. Reads were classified by Kraken 2 using the PlusPF database, which includes archaea, bacteria, virus, protozoa, and fungi [38,39]. Analysis was performed in R version 3.4.0 [40]. A list of R packages used in the analysis and their versions is in the Supplemental Methods. Composition was assessed by Bray-Curtis, diversity by Shannon index, and dominance by Berger-Parker Index. Statistical testing included PERMANOVA or Wilcoxon rank-sum as indicated.

Blood isolate WGS sequences (**S3 Table**) were demultiplexed through bcl2fastq v2.19.0.316 and processed using the Sunbeam pipeline. The processed sequencing reads were assembled using Unicycler v0.4.7 with the Illumina-only assembly method [41]. Genomes were quality checked and pangenome analysis performed by CoreSNPs as described [42].

Sequence data are available at Genbank or SRA under BioProjectID PRJNA848838.

### Gut/BSI analysis

To establish a threshold for defining identity between gut and BSI organisms we first generated a matrix of unrelated strains for each identified BSI species and calculated single nucleotide variants per megabase pair (SNV/Mbp) from all within-species alignments. WGS were downloaded from Genbank (**S4 Table**), including only a single sequence from any project in an effort to avoid related strains. WGS from BSI isolates in this study were included in this matrix as well. Out of 2296 pair-wise within-species comparisons, 20 (0.9%) fell below 100 SNP/Mbp (**S1 Fig**). The purportedly unrelated pairs that were below this threshold were then subject to investigation as to the origin of the isolates sequenced (**S5 Table**). This analysis revealed that seven of the 20 pairs were actually independent sequences of the same strain or derivatives of the strain. Another three pairs of sequences were reportedly different strains though were submitted from the same institutions. Three pairs of sequences had no obvious connection but actually matched a widely-used reference strain. The remaining seven showed no obvious connection. Once the 10 clearly-related strain comparisons were removed, the 99^th^ percentile for SNV/Mbp within each species was re-calculated and employed as a threshold, with 100 SNV/Mbp employed as the maximum threshold for identity (**S6 Table**). Further details are in Supplemental Methods.

Reads from stool were mapped to assembled blood isolate WGS and reference genomes using a version of hisss modified to incorporate bcftools v1.8 to extract variants calls from mapped reads against the genomes [43]. Variant calls were considered if they had >7-fold coverage, >30 PHRED quality score, and >14 mapping quality score. The degree of matching between stool reads and both cognate BSI and unrelated species-matched WGSs was quantified by SNV/Mbp from the alignments. We also calculated the SNV/Mbp expected from sequencing error alone based on a single genome re-sequenced 39 time [42] and from within-patient heterogeneity using WGS from three sequential positive blood cultures on a single patient, as detailed in Supplemental Methods.

## Results

### Patient cohort and BSI episodes

We enrolled 139 critically ill patients with at least one stool sample (**Table 1**). The population was heterogenous (**S1 Table**) and included 24 patients (17.3%) with hematologic malignancy (leukemia, lymphoma, myelodysplastic syndrome), 32 (23%) who received cytotoxic chemotherapy, and 9 (6.5%) with neutropenia. Of the subjects, 57 had one or more positive blood cultures (excluding *Staphylococcus epidermidis*; see Methods) (**Table 1**), including 27 with more than one episode or organism.

**Fig 1:**
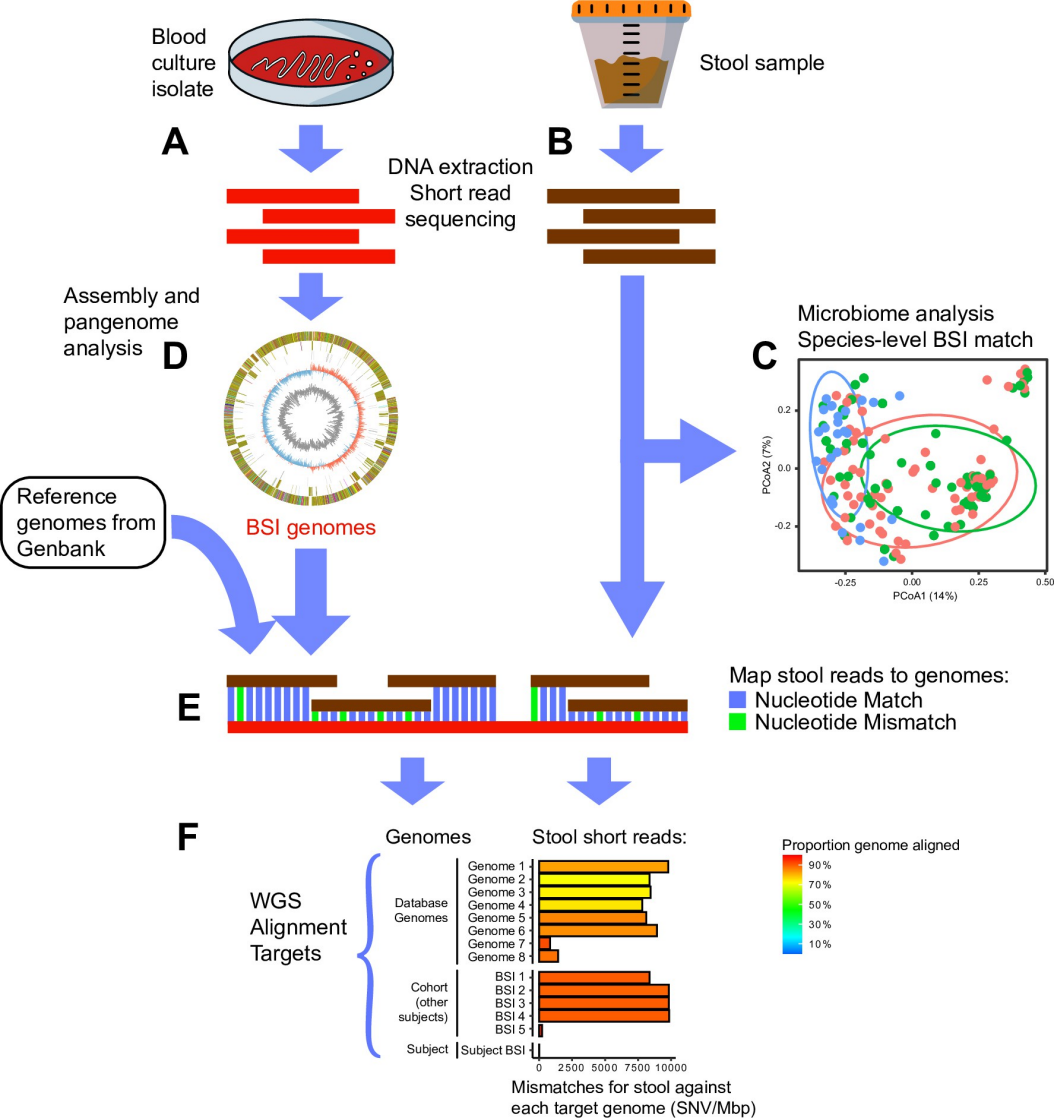
Schematic of gut microbiome and BSI whole genome sequence (WGS) analysis. BSI isolates (A) and stool (B) were subjected to shotgun metagenomic deep sequencing. Stool samples were used for microbiome analysis of composition, diversity and dominance (C). BSI isolates were used for assembly of whole genomes (D). Stool and BSI WGS together were used to test identity between organisms in the stool and blood culture isolates by mapping stool reads to the cognate BSI WGS (E). Single nucleotide variants per mega base pair (SNV/Mbp) was calculated to represent the degree of mismatch, and WGS of BSI isolates of same species from the cohort (if available) and reference genomes downloaded from databases were included as control comparisons (hypothetical example shown in F).

There were 86 organisms identified from the 57 patients. Of these, 23 unique blood isolates from 21 patients were available for WGS. Stool was subject to shotgun sequencing with mean 7,303,821 reads/sample (**S2 Table)**. WGS of BSI isolates yielded a mean 2,141,628 reads/sample, and assembly produced genomes with average 97.49% completeness and depth of 78.81 (**S3 Table**). **Fig 1** shows an overview of the analysis.

### Structure of the gut microbiome in critically ill patients

**Fig 2:**
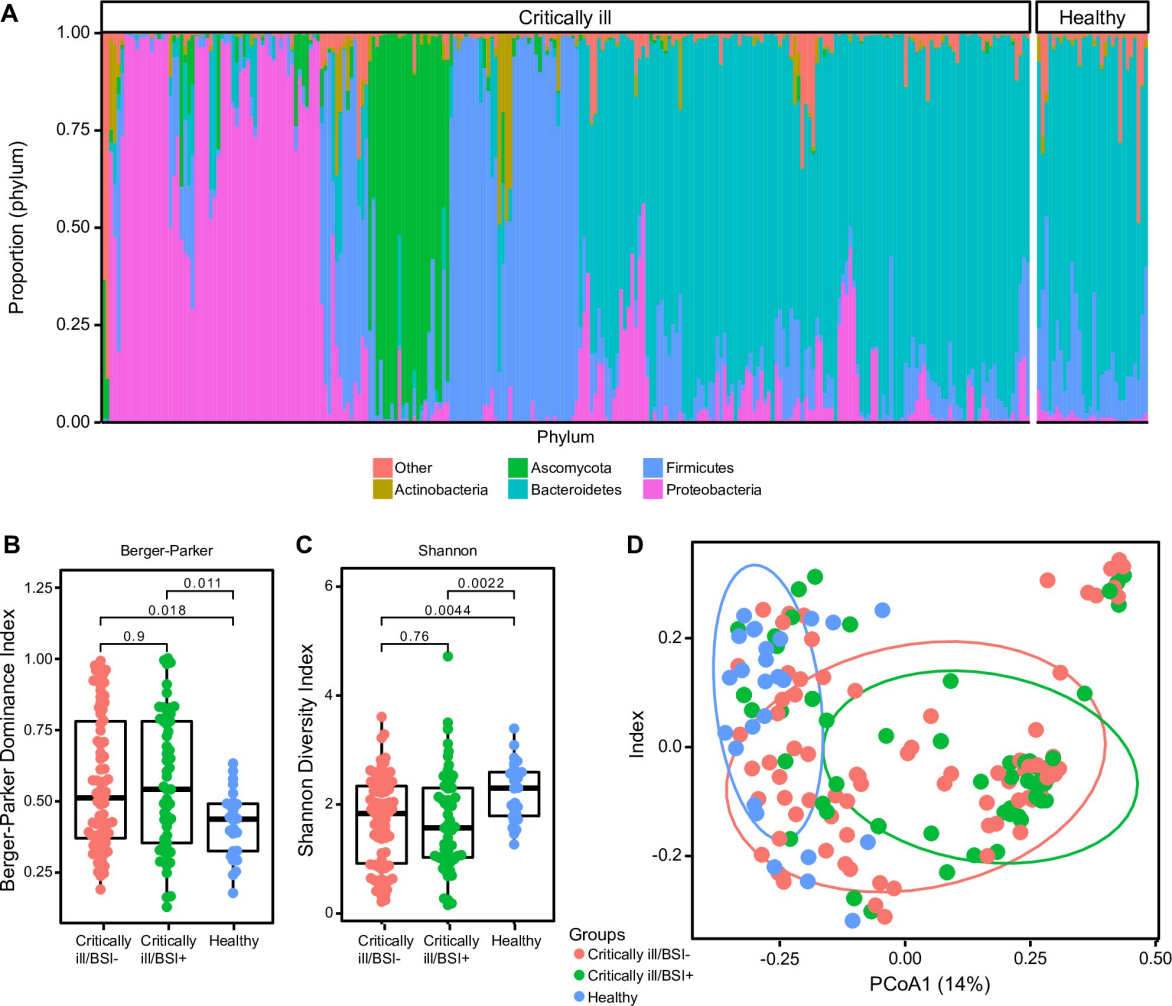
Gut microbiota of critically ill patients are dysbiotic but do not differ among those with or without bloodstream infection. Stool samples all critically ill subjects enrolled in the study were compared with healthy individuals reported from the Human Microbiome Project (HMP). (A) Bar graph representing phylum level composition of critically ill and healthy subjects clustered by Bray-Curtis dissimilarity. Some critically ill subjects’ gut microbiota resembles the gut microbiome composition of healthy individuals, while others are dysbiotic and dominated by Proteobacteria, Firmicutes, or fungi (Ascomycota). (B) Alpha diversity using the Shannon index. Critically ill subjects are colored based on whether they did (BSI+; green) or did not (BSI-; red) have a bloodstream infection. Healthy and critically ill are significantly different (p = 0.003; Wilcoxon rank-sum with Benjamini-Hochberg adjustment for false discovery) but there is no difference between critically ill/BSI + and critically ill/BSI- groups. (C) Dominance by the Berger-Parker dominance index. Healthy and critically ill are significantly different (p = 0.008; Wilcoxon rank-sum with Benjamini-Hochberg adjustment for false discovery), but there is no difference between critically ill/BSI+ and critically ill/BSI- groups. (D) Principal coordinate analysis of stool microbiota based on Bray-Curtis dissimilarity. Critically ill subjects diverge significantly from healthy individuals (PERMANOVA with Benjamini-Hochberg adjustment for false discovery; p = 0.008) but critically ill/BSI+ and critically ill/BSI- do not differ from each other from each other. Panel A includes all stool samples, while panels B-D include just the first collected sample for each subject.

We analyzed the gut microbiome of our patients, shown as a bar chart of relative abundances at the phylum level in **Fig 2A**. Compared to healthy individuals, critically ill patients overall had significantly lower gut microbiome diversity (a measure of both number of taxa and their evenness of distribution; Shannon index; mean critically ill: 1.63 vs healthy: 2.16; p = 0.003; Wilcoxon rank-sum; **Fig 2B**), higher dominance (the proportion of the microbiome comprised of single taxa; Berger-Parker index; mean critically ill: 0.5569 vs healthy: 0.4137; p = 0. 008; Wilcoxon rank-sum; **Fig 2C**) and distinct composition by Bray-Curtis analysis (an integrated measure of composition dissimilarity; PERMANOVA; p = 0.008; **Fig 2D**). There was considerable heterogeneity within the critically ill group and many were similar to healthy subjects (**Fig 2A**), There were no differences between critically ill patients who did or did not experience BSI in diversity (p = 0.756), dominance (p = 0.903) or global composition (p = 0.194) (**Fig 2B–2D**), though some of the stool samples were not contemporaneous with BSI episodes.

### Association between gut microbiome constituents and BSI

We examined 86 BSIs in which stool was available within approximately one month, and queried whether the BSI species was present within the gut microbiota **(Table 2)**, focusing on samples with highest relative abundance of the BSI species if multiple specimens were available. The BSI species were present in gut microbiota at the threshold typically defined as “dominant” (≥30% relative abundance (10,25)) in 9 episodes (10%). For 12 episodes (14%) the BSI species were present at ≥10% relative abundance, and in 20 (23%) were present at ≥2.5%. An additional 23 (27%) were detectable at low levels (0.01–2.5%). In contrast, species-matched taxa were essentially absent in gut (<0.01% relative abundance) in 43 episodes (50%). The relationship between BSI species representation in gut and selected clinical conditions is shown in Table 3. Because some of the stool samples were temporally distant from the BSI, this enables identification of related species for further interrogation but precludes conclusions as to the frequency of contemporaneous stool/BSI concordance.

**Table 2:** Species-level detection of BSI isolates in gut microbiome. BSI organism species-level representation in the gut microbiome. Data reflect the number of episodes in which the gut microbiome specimen with highest relative abundance of the BSI species was detected at the indicated relative abundance threshold.

BSI species representation in gut microbiome	BSI episodes (n = 86)	Percent of total
<0.01% relative abundance	43	50%
≥0.01% relative abundance	43	50%
≥2.5% relative abundance	20	23%
≥10% relative abundance	12	14%
≥30% relative abundance	9	10%

**Table 3:** Association between detection of BSI species in the gut microbiome and selected underlying conditions.

	N = 86	<0.01% stool match	≥0.01% stool match	≥2.5% stool match	≥10% stool match	≥30% stool match
Heme malignancy	present (n = 20)	9	11	7	5	5
	absent (n = 66)	34	32	13	7	4
	Odds ratio (95% CI)	REF	1.30 (0.48–3.55)	2.03 (0.63–4.21)	2.70 (0.69–5.07)	4.72 (1.05–5.86)
Neutropenic	present (n = 5)	2	3	1	1	1
	absent (n = 81)	41	40	19	11	8
	Odds ratio (95% CI)	REF	1.54 (0.24–9.70)	1.08 (0.09–18.02)	1.86 (0.15–18.56)	2.56 (0.21–19.06)
Cytotoxic chemo	present (n = 19)	10	9	6	4	3
	absent (n = 67)	33	34	14	8	6
	Odds ratio (95% CI)	REF	0.87 (0.31–2.42)	1.41 (0.43–2.87)	1.65 (0.41–3.52)	1.65 (0.35–4.41)
Immuno-suppressive therapy	present (n = 22)	11	11	7	5	5
	absent (n = 64)	32	32	13	7	4
	Odds ratio (95% CI)	REF	1.00 (0.38–2.63)	1.57 (0.5–3.15)	2.08 (0.55–3.81)	3.64 (0.82–4.40)
Any of these factors	present (n = 26)	13	13	9	7	6
	absent (n = 60)	30	30	11	5	3
	Odds ratio (95% CI)	REF	1.00 (0.40–2.51)	1.89 (0.86–3.74)	3.23 (0.86–3.74)	4.62 (1.00–4.62)

### Establishing a threshold for related versus unrelated sequences

To interrogate the relatedness between microbial constituents in gut and BSI isolates stringently, we subjected the 23 available BSI isolates to whole genome sequencing. We then aligned stool reads from each subject with the WGS of their cognate blood isolate, with WGS from other subjects in the cohort with the same species BSI, and with genomes of that species from GenBank. To quantify the degree of matching between BSI isolate sequences and stool reads, we calculated single nucleotide variants per megabase pair (SNV/Mbp) from the alignments.

To define a threshold for related versus unrelated whole genome sequences, we first estimated the variability that might occur based on biological variability and sequence error alone. We previously carried out re-sequencing of a single *Cholera vibrio* genome 39 times and found the average difference between genomes was 3.6 SNV/Mbp core genomes [42]. We also carried out WGS on three independent blood culture isolates obtained on sequential days from a subject with *E*. *faecalis* BSI (**S3 Table**); two comparisons resulted in 3.12 SNV/Mbp (S127_1 vs S127_2 and S127_2 vs S127_3) and one resulted in 1.25 SNV/Mbp (S127_1 vs S127_2) between sequences. These findings suggest a level of difference that might be expected based on sequence error and authentic intra-host variability.

We then compared sequences from unrelated genomes belonging to each species reported by blood culture (or in the case of one *Achromobacter* BSI, genus-level). To do this, we downloaded only one randomly selected sequence from each study to minimize the likelihood of including related organisms and examined the distribution of pair-wise comparisons (**S1 Fig**). We found a median of 9593.5 SNVs per Mbp between unrelated strains. However, we identified several pair-wise comparisons that were unexpectedly low. Investigation of these closely related whole genome sequences revealed that several were actually replicate sequences of the same strains, while other had no obvious relation (**S5 Table**). Based on this information, we established thresholds that excluded >99% of alignments between unrelated genomes within each species, and cognate stool/BSI whole genome sequence alignments that fell below that threshold was considered a match (**S6 Table**).

### Relationship between gut microbiota and BSI through WGS

**Fig 3:**
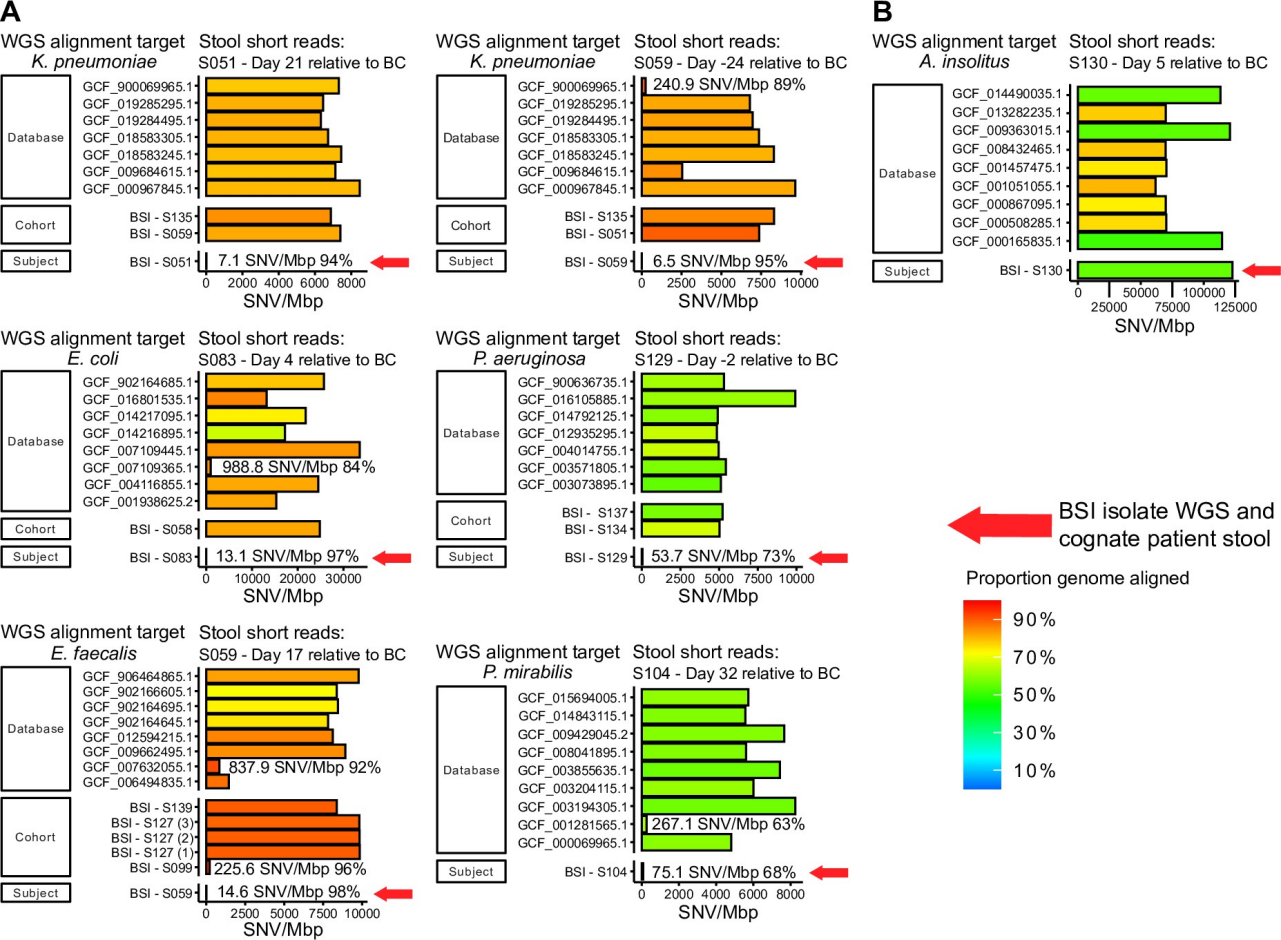
Mapping stool reads against BSI whole genome sequences (WGS). Each available BSI isolate was subjected to whole genome shotgun sequencing and then assembly into WGS (mean 97.49% coverage; mean 78.81X depth). Stool short reads were then aligned with multiple genomes (indicated on the left of the graph), which included the cognate BSI WGS (“subject”), the WGS of other subjects’ BSI organisms of the same species if any (“cohort”), and unrelated species-matched WGS downloaded from Genbank (“database”). Sequence similarity was calculated based on mismatches defined as single nucleotide variant per megabase pair (SNV/Mbp), reflected along the X axis for each stool sample compared with multiple genomes. (A) Results for six BSI episodes where stool and BSI WGS showed sequence-based strain identity. The SNV/Mbp is indicated along the X axis for each alignment; for clarity, the value is shown for those less than 1000 SNV/Mbp followed by the proportion genome aligned. The red arrow indicates the cognate BSI WGS matched to the stool sample. Each bar is colored to indicate the proportion of genome aligned with stool short reads (because of the short bar, the proportion of genome aligned is shown as a fraction next to the SNVs for the low-SNV matched sample. (B) Representative example of a stool/BSI WGS that did not match. Stool alignment with the cognate *Achromobacter* BSI WGS showed SNV/Mbp that was no lower than when compared with unrelated WGS of *Achromobacter* (which was identified only at the genus level by the clinical microbiology lab) downloaded from GenBank.

Among the 23 BSI isolates with whole genome sequences available, 9 were from BSI episodes with essentially no species-level gut representation (<0.01%), while 14 were from episodes with BSI species present at ≥0.01% relative abundance in gut, including 6 with ≥2.5% and 2 with ≥30% relative abundance **(Tables 4 and S1)**. Among these 14 BSIs where the BSI species was present at ≥0.01% relative abundance in gut, application of our sequence identity threshold showed that 8 did not match by sequence alignment, indicating that BSI and stool sequences represented different strains, while 6 showed high sequence similarity. The 6 BSI episodes where the stool reads matched the BSI organism WGS included two *K*. *pneumonia*, and one each with *E*. *coli*, *P*. *mirabilis*, *P*. *aeruginosa*, *and E*. *faecalis*. **Fig 3A** shows these 6 alignments. Sequence identity matching was more frequent when the BSI species was present at higher abundance in the gut microbiome (e.g.; sequence matched in 5/6 episodes where the gut microbiome relative abundance was ≥2.5% but only 1/8 where relative abundance in gut was <2.5%) **(Table 4)**.

**Table 4:** BSI episodes where species were detected in gut. Subjects are ordered by the relative abundance of BSI species in highest abundance stool sample, and grouped into those with dominant (≥30%), 2.5–30%, and 0.1–2.5% relative abundances. The last column indicates whether the stool reads matched the BSI isolate WGS for those episodes where the BSI isolate was available for sequencing.

		Stool dated closest to BC		Stool with maximum relative abundance of BC organism		
Subject	Blood Culture Organism	Days relative to BC	Relative Abundance (%)	Days relative to BC	Relative Abundance (%)	WGS stool match
S071	*Candida albicans*	8	81.02	8	81.02	
S067	*Candida albicans*	4	78.76	4	78.76	
S098	*Enterococcus faecium*	30	78.44	30	78.44	
S130	*Achromobacter species*	5	77.66	5	77.66	NO
S076	*Bacteroides fragilis*	-4	68.34	-4	68.34	
S020	*Escherichia coli*	4	53.76	4	53.76	
S111	*Klebsiella pneumoniae*	29	1.78	32	43.44	
S047	*Pseudomonas aeruginosa*	11	42.11	11	42.11	
S059	*Klebsiella pneumoniae*	15	0.06	-21	39.37	YES
S101	*Proteus vulgaris*	6	28.48	6	28.48	
S048	*Klebsiella pneumoniae*	15	0.02	29	22.75	
S084	*Escherichia coli*	-19	10.77	-19	10.77	
S002	*Escherichia coli*	1	8.61	1	8.61	
S005	*Klebsiella pneumoniae*	1	7.97	1	7.97	
S059	*Enterococcus faecalis*	-1	0.86	16	6.17	YES
S122	*Candida albicans*	-37	5.14	-37	5.14	
S051	*Klebsiella pneumoniae*	7	0.19	21	4.57	YES
S083	*Escherichia coli*	4	3.88	4	3.88	YES
S104	*Proteus mirabilis*	10	0.8	20	3.09	YES
S082	*Pseudomonas aeruginosa*	1	2.97	1	2.97	
S047	*Klebsiella pneumoniae*	15	2.15	15	2.15	
S129	*Pseudomonas aeruginosa*	-2	1.26	-2	1.26	YES
S139	*Enterococcus faecalis*	3	0.97	3	0.97	NO
S022	*Pseudomonas aeruginosa*	20	0.92	20	0.92	
S099	*Enterococcus faecalis*	-1	0.49	-1	0.49	NO
S047	*Stenotrophomonas maltophilia*	5	0.49	5	0.49	
S114	*Staphylococcus aureus*	1	0.41	1	0.41	
S134	*Staphylococcus haemolyticus*	22	0.37	22	0.37	NO
S019	*Escherichia coli*	-1	0.35	-1	0.35	
S083	*Enterococcus faecium*	16	0.28	16	0.28	
S119	*Gemella morbillorum*	1	0.24	1	0.24	
S003	*Staphylococcus aureus*	0	0.18	0	0.18	
S106	*Streptococcus gallolyticus*	2	0.04	3	0.11	
S037	*Pseudomonas aeruginosa*	9	0.07	9	0.07	
S047	*Candida albicans*	0	0.07	0	0.07	
S101	*Bacteroides ovatus*	4	0.06	4	0.06	NO
S097	*Mycobacterium abscessus*	2	0.04	2	0.04	
S105	*Escherichia coli*	1	0.04	1	0.04	NO
S019	*Enterococcus faecalis*	5	0.03	5	0.03	
S087	*Klebsiella pneumoniae*	18	0.02	18	0.02	
S135	*Klebsiella pneumoniae*	11	0.01	11	0.01	NO
S064	*Staphylococcus aureus*	2	0.01	2	0.01	
S078	*Morganella morganii*	12	0.01	12	0.01	NO

One surprising result was a BSI with *Achromobacter* (identified by blood culture only at the genus level as *Achromobacter* species). *Achromobacter* comprised 77% of a near-contemporaneous gut microbiome sample. Unexpectedly, gut sequences were no more closely related to the cognate BSI whole genome sequence than to unrelated *Achromobacter* genomes from databases (**Fig 3B**). WGS of the blood culture isolate revealed an *Achromobacter insolitus* genome, and metagenomic stool sequencing revealed that *A*. *insolitus* existed within the gut microbiome at low relative abundance (0.65%) while the majority of gut reads were other *Achromobacter* species. This patient had a respiratory tract infection that grew *Achromobacter* species; while the respiratory tract isolate was not available for sequencing, the lack of alignment between BSI and gut sequences gut suggests the lungs may have been responsible for the BSI despite high *Achromobacter* abundance in gut. Thus, this patient appeared to have two independent strains of *Achromobacter* present, emphasizing the importance of sequence-based identity confirmation.

## Discussion

We investigated the relationship between gut microbiome and BSI organisms in critically ill adults, a population that has a high rate of both BSI and dysbiosis, but has not been studies for gut microbiome-BSI relationships. The species responsible for BSI of about half of these BSI episodes were detectable in gut, and were dominant gut taxa in 10%, even though some of the stool samples were relatively distant from the time of BSI. Sequence-based comparison of the gut and available BSI isolate sequences confirmed strain-level identity in half of these strain-level matched episodes, which included most but not all (5/6) episodes associated with ≥2.5% gut representation, though few (1/8) with lower levels of gut representation.

Key to interrogating the relatedness of microbiota present at different sites is to employ a threshold that can distinguish the same versus unrelated strains. We found that it was sometimes challenging to unravel the origins of bacterial isolates linked to whole genome sequences present in public databases. Based on the unexpectedly close relationship between sequences from different studies, we attempted to track down their origins. We discovered that several were in fact sequences of the same bacterial isolate that had been reported in the literature under different names, such as *Achromobacter insolitis* strains LMG 6003 (GCF_002209555.1) and DSM 23807 (GCF_001971645.1), and *A*. *xylosoxidans* strains KM54*3 (*GCF_000508285.1 and GCF_001457475.1). In several other cases, we found multiple independent deposits of the same strain such as *P*. *aeruginosa* strains PA01 (GCF_900070375.1, GCF_004014755.1, GCF_013305765.1, and GCF_004102665.1) and PA14 (GCF_900095805.1, GCF_000014625.1), explaining their close similarity. In contrast, we found other closely related sequences that were reported by different studies but from the same institution, such as *P*. *mirabilis* strains T21 (GCF_002310875.1) and L90-1 (GCF_013343255.1), and other closely related strains were seen we were unable to find a link between the isolates. However, because those sequences were more closely related than the majority of sequence comparisons, we raise the question of whether obscure origins or even cross-contamination among laboratory-passaged bacterial isolates might be responsible.

The six BSIs where we confirmed sequence identity with gut organisms included two episodes of *K*. *pneumonia*, and one each of *E*. *coli*, *E*. *faecium*, *P*. *mirabilis* and *P*. *aeruginosa*. All of these are well-recognized gut-derived pathogens. Sequence identity would be consistent with a gut origin for the BSI. However, when investigating the gut as a potential source, directionality can be difficult to discern, as both gut and BSI could be seeded from other sources. Compounding this, some of our stool samples with sequence matching were temporally distant from the BSI. Thus, we cannot establish directionality for certain or exclude the possibility that both gut and blood may have been seeded from another site, or that gut seeded an intermediate site (e.g.; skin) that then led to BSI, or other potential relationships.

Unexpectedly, we had one episode of *Achromobacter* BSI where the strain did not match the gut sequences even though *Achromobacter* represented 77% of gut taxa sampled only 5 days apart. We hypothesize that the BSI may have been linked to a contemporaneous respiratory tract infection, emphasizing that even in the context of high-level gut dominance, confirming the identity would be critical to track the origin of BSI.

Several other BSI episodes were associated with high representation in the gut but for which the BSI isolate was not available for WGS. Those with >30% gut representation included several additional BSIs with these organisms, and two BSIs with *Candida albicans* that represented ~80% of near-contemporaneous gut specimens. Gut colonization is increasingly recognized as a driver of systemic candidemia and clinical outcomes [44–47], though whole genome sequences were not available here to confirm identity.

Our study has several limitations. By design, our cohort was a mixed group of critically ill patients representing a common population at risk for BSI, but this heterogeneity may obscure relationships present in subpopulations. Enrollment focused on patients with suspected sepsis or confirmed BSI, and blood cultures were obtained based on clinical justification rather than systematic screening, so these findings may not be generalizable to all ICU patients. Stool samples were sometimes not available prior to BSI and several were from relatively distal timepoints, and so it is not possible for us to impute directionality and gut translocation as the origin of these BSIs. Blood isolates were available for WGS in only a subset of patients. Finally, we focused on culture-positive BSI, but translocation of gut microbiome products has been implicated as a driver of sepsis and multisystem organ failure in critically ill patients in the absence blood culture positivity [48–51], which reflects a distinct potential mechanism of microbiome-driven pathogenesis.

In summary, our findings provide insight into the relationship between organisms responsible for BSIs in critically ill patients and the gut microbiome. We highlight the importance of sequence-based confirmation of identity, and the challenge of database genomes that may have obscure origins and reflect related isolates. Our data suggests that members of the gut microbiome were identical to the BSI and thus could have originated from the gut, warranting further investigation. Future studies may be warranted to test whether monitoring the gut microbiome in ICU patients for dominant taxa might have value in predicting BSI and tailoring initial antimicrobial therapy. In addition, an avenue for future research is whether there might be benefit to interventions that suppress highly dominant communities in some critically ill patients using targeted antimicrobial prophylaxis or microbiome transplantation, or other microbiome-targeted therapeutics [50,52–54].

## Supporting information

S1 Fig Distribution of within-species pairwise comparisons. (PDF)

S1 Table Clinical features associated with BSI episodes where BSI species was detected in gut microbiome (≥0.01%). (PDF)

S2 Table Metagenomic stool samples analyzed. (PDF)

S3 Table Whole genome sequence assembled from blood culture isolates. (PDF)

S4 Table Reference genomes used for pangenome analysis. (PDF)

S5 Table Pair-wise comparisons between purportedly unrelated strains within species. (PDF)

S6 Table SNV/Mbp analysis of unrelated reference and study genomes per species. (PDF)

S1 File (PDF)
