# Supplementary material for: Tracking gut microbiome and bloodstream infection in critically ill adults
Source: PLoS One. 2023 Oct 10;18(10):e0289923. doi: 10.1371/journal.pone.0289923 (PMC10564172; doi:10.1371/journal.pone.0289923)
Supplement: S1 Fig — (PDF) [file pone.0289923.s001.pdf]

Supplemental Figure

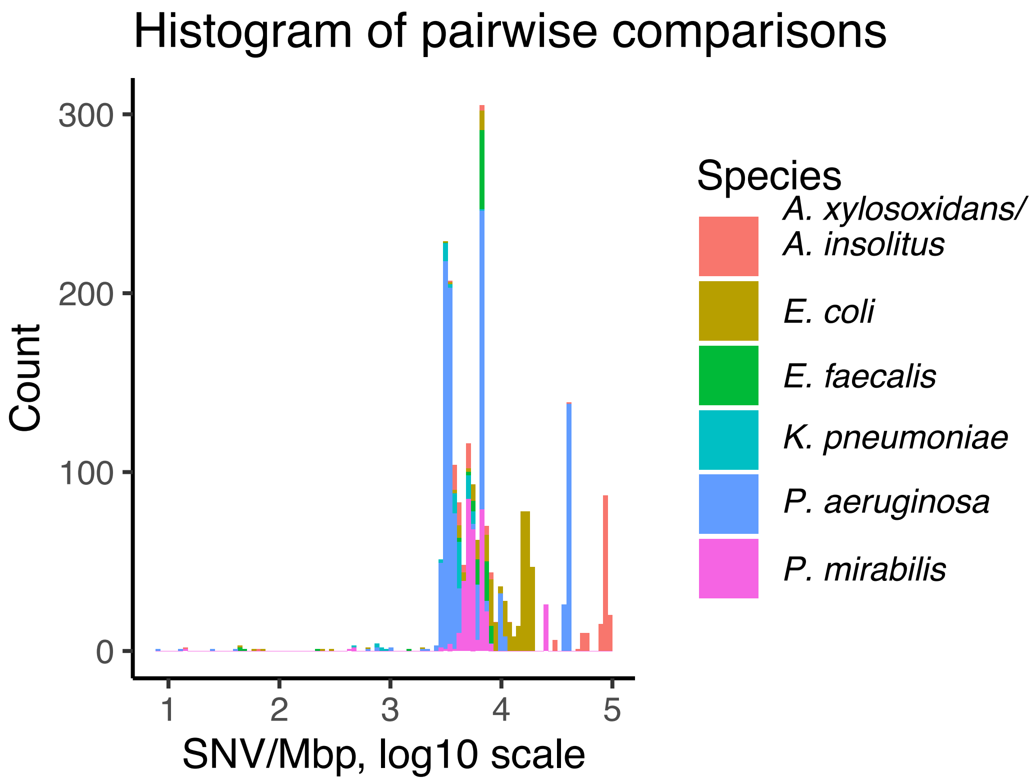

Figure S1. Distribution of within-species pairwise comparisons. Histogram of 2296 comparisons, each comparison done against another genome of the respective species. The x axis represents bins of SNV/Mbp core genome comparisons and the y axis is the number of genomes falling into the respective SNV/Mbp bins; there are 100 bins total for the x axis. Color of the column represents the species contributing to the count. The *A. insolitus* within-species comparison was combined with the *A. xylosoxidans* within-species comparison due to low number of complete unique reference genomes.
