# Supplementary material for: Tracking gut microbiome and bloodstream infection in critically ill adults
Source: PLoS One. 2023 Oct 10;18(10):e0289923. doi: 10.1371/journal.pone.0289923 (PMC10564172; doi:10.1371/journal.pone.0289923)
Supplement: S1 Table — (PDF) [file pone.0289923.s002.pdf]

Table S1. All BSI episodes with BSI species detected in gut microbiome (≥0.01%)

		Stool dated closest to BC		Stool with maximum relative abundance of BC organism									
Subject	Blood Culture Organism	Days relative to BC	Relative Abundance (%)	Days relative to BC	Relative Abundance (%)	WGS available	WGS match to stool	Major diagnoses	Hematologic malignancy	Solid Cancer	Neutropenia at time of BSI	Prior cytotox chemo	immunosupp therapy
S002	Escherichia coli	1	8.61	1	8.61	NO		Colitis, GI bleeding, chronic neurological deficit; chronic trach/PEG	NO	NO	NO	NO	NO
S003	Staphylococcus aureus	0	0.18	0	0.18	NO		cirrhosis, AKI, resp failure	NO	NO	NO	NO	NO
S005	Klebsiella pneumoniae	1	7.97	1	7.97	NO		AML, s/p allo-BMT	YES	NO	YES	YES (1 m earlier, & TBI)	YES
S019	Enterococcus faecalis	5	0.03	5	0.03	NO		panc cancer, biliary obstruction, liver abscesses	NO	YES	NO	NO	NO
S019	Escherichia coli	-1	0.35	-1	0.35	NO		panc cancer, biliary obstruction, liver abscesses	NO	YES	NO	NO	NO
S020	Escherichia coli	4	53.76	4	53.76	NO		cirhosis	NO	NO	NO	NO	NO
S022	Pseudomonas aeruginosa	20	0.92	20	0.92	NO		AML, resp failure	YES	NO	YES	YES	YES
S037	Pseudomonas aeruginosa	9	0.07	9	0.07	NO		cirrhosis, chronic chylothorax, TPN	NO	NO	NO	NO	NO
S047	Candida albicans	0	0.07	0	0.07	NO		acute cholangitis, hypoNa, AKI, CAD, chronic osteo	NO	NO	NO	NO	NO
S047	Klebsiella pneumoniae	15	2.15	15	2.15	NO		acute cholangitis, hypoNa, AKI, CAD, chronic osteo	NO	NO	NO	NO	NO
S047	Pseudomonas aeruginosa	11	42.11	11	42.11	NO		acute cholangitis, hypoNa, AKI, CAD, chronic osteo	NO	NO	NO	NO	NO
S047	Stenotrophomonas maltophilia	5	0.49	5	0.49	NO		acute cholangitis, hypoNa, AKI, CAD, chronic osteo	NO	NO	NO	NO	NO
S048	Klebsiella pneumoniae	15	0.02	29	22.75	NO		SJS, resp failure, HSV/CMV	NO	NO	NO	NO	YES
S051	Klebsiella pneumoniae	7	0.19	21	4.57	YES	YES	ESRD, respiratory failure, acute stroke, chronic ostemyelitis	NO	NO	NO	NO	NO
S059	Enterococcus faecalis	-1	0.86	16	6.17	YES	YES	ALL, APLS with venous clots	YES	NO	NO	NO	YES
S059	Klebsiella pneumoniae	15	0.06	-21	39.37	YES	YES	ALL, APLS with venous clots	YES	NO	NO	NO	YES
S064	Staphylococcus aureus	2	0.01	2	0.01	NO		cirrhosis, PNA, acute resp failure, AKI	NO	NO	NO	NO	NO
S067	Candida albicans	4	78.76	4	78.76	NO		cirrhosis, resp failure, PNA, yeast UTI	NO	YES	NO	NO	NO
S071	Candida albicans	8	81.02	8	81.02	NO		met GB cancer, resp failure	NO	YES	NO	YES (2 wk)	NO
S076	Bacteroides fragilis	-4	68.34	-4	68.34	NO		T cell lymphoma, neutropenia, colitis/tiflitis, resp failure, AKI	YES	NO	YES	YES	NO
S078	Morganella morganii	12	0.01	12	0.01	YES	NO	Myeloma, COVID19	YES	NO	YES	YES	NO
S082	Pseudomonas aeruginosa	1	2.97	1	2.97	NO		SJS, GBS meningitis, acute resp failure	NO	NO	YES	NO	NO
S083	Enterococcus faecium	16	0.28	16	0.28	NO		ESRD, CAD, atrial thrombus, osteomyelitis	NO	NO	NO	NO	NO
S083	Escherichia coli	4	3.88	4	3.88	YES	YES	ESRD, CAD, atrial thrombus, osteomyelitis	NO	NO	NO	NO	NO
S084	Escherichia coli	-19	10.77	-19	10.77	NO		cirrhosis, ICH, C. diff colitis	NO	NO	NO	NO	NO
S087	Klebsiella pneumoniae	18	0.02	18	0.02	NO		AML (refractory)	YES	NO	YES	NO	YES
S097	Mycobacterium abscessus	2	0.04	2	0.04	NO		MDS, S/P Allo BMT, COVID19, lung GVHD & m. abcessus infection	YES	NO	NO	NO	YES
S098	Enterococcus faecium	30	78.44	30	78.44	NO		ALL 40 d s/p Allo BMT, urinary obstruction	YES	NO	NO	YES (49 d prior)	YES
S099	Enterococcus faecalis	-1	0.49	-1	0.49	YES	NO	HIV, Pulm HTN, chronic resp failure, AKI	NO	NO	NO	NO	NO
S101	Bacteroides ovatus	4	0.06	4	0.06	YES	NO	cellulitis, C. diff colitis, AKI	NO	NO	NO	NO	NO
S101	Proteus vulgaris	6	28.48	6	28.48	NO		cellulitis, C. diff colitis, AKI	NO	NO	NO	NO	NO
S104	Proteus mirabilis	10	0.8	20	3.09	YES	YES	ESRD, stroke, resp failure	NO	NO	NO	NO	NO
S105	Escherichia coli	1	0.04	1	0.04	YES	NO	acute resp failure, chronic venous insufficiency, opioid & alcohol use	NO	NO	NO	NO	NO
S106	Streptococcus bovis	2	0.04	3	0.11	NO		Panc neuro endocrine tumor, portal vein thrombosis, colitis	NO	YES	NO	NO	NO
S111	Klebsiella pneumoniae	29	1.78	32	43.44	NO		MDS, allo-BMT, acute resp failure	YES	NO	YES	YES	YES
S114	Staphylococcus aureus	1	0.41	1	0.41	NO		bladder cancer, urinary obstruction, recurrent UTIs, bowel obstruction, TPN	NO	YES	NO	NO	NO
S119	Gemella morbillorum	1	0.24	1	0.24	NO		asthma, acute hemolytic anemia	NO	NO	NO	NO	NO
S122	Candida albicans	-37	5.14	-37	5.14	NO		chronic VDRF, recurrent PNA, DM, s/p HHNK, gastroparesis	NO	NO	NO	NO	NO
S129	Pseudomonas aeruginosa	-2	1.26	-2	1.26	YES	YES	COVID19, acute resp failure	NO	NO	NO	NO	NO
S130	Achromobacter species	5	77.66	5	77.66	YES	NO	resp failure, AKI, PNA, DVT/PE, pul HTN	NO	NO	NO	NO	NO
S134	Staphylococcus haemolyticus	22	0.37	22	0.37	YES	NO	aortic aneurism repair, AKI, abd compartment	NO	NO	NO	NO	NO
S135	Klebsiella pneumoniae	11	0.01	11	0.01	YES	NO	urosepsis, AKI	NO	NO	NO	NO	NO
S139	Enterococcus faecalis	3	0.97	3	0.97	YES	NO	COVID19, ECMO	NO	NO	NO	NO	NO

MDS, myelodysplastic syndrome; BMT, bone marrow transplantation; GVHD, graft versus host disease; ALL, acute lymphoblastic leukemia; HTN, hypertension; AKI, acute kidney injury; ESRD, end stage renal disease; TPN, total parenteral nutrition; DVT, deep vein thrombosis; PE, pulmonary embolism; ECMO, extracorporeal membrane oxygenation; AML, acute myelogenous leukemia; SJS, Stevens-Johnson syndrome; PNA, pneumonia; GB, gallbladder; GBS, group B
