# Supplementary material for: Tracking gut microbiome and bloodstream infection in critically ill adults
Source: PLoS One. 2023 Oct 10;18(10):e0289923. doi: 10.1371/journal.pone.0289923 (PMC10564172; doi:10.1371/journal.pone.0289923)
Supplement: S2 Table — (PDF) [file pone.0289923.s003.pdf]

Table S2. Gut microbiome samples

"SAMN" indicates samples from subjects in this cohort sequenced here, while "SRR" are randomly-selected healthy gut microbiome data downloaded from the Human Microbiome Project.

Accession	SID	SampleID	type	total_reads	host	nonhost	proportion_nonhost	proportion_nonhost_identified
SAMN29636406	S088	S088_44204	STL	5022344	885	5021459	0.999823787	0.976825859
SAMN29636407	S089	S089_44207	STL	11150987	559216	10591771	0.949850538	0.92352176
SAMN29636408	S089	S089_44231	STL	2948672	912	2947760	0.999690708	0.994823866
SAMN29636409	S090	S090_44186	STL	7189518	4602500	2587018	0.359831911	0.85106559
SAMN29636410	S091	S091_44204	STL	5271841	117556	5154285	0.977701148	0.804122007
SAMN29636411	S091	S091_44211	STL	2331138	329	2330809	0.999858867	0.858479609
SAMN29636412	S092	S092_44211	STL	11380404	468	11379936	0.999958877	0.689385424
SAMN29636413	S092	S092_44232	STL	6862603	395	6862208	0.999942442	0.775951268
SAMN29636414	S093	S093_44209	STL	5943624	675	5942949	0.999886433	0.61052703
SAMN29636415	S094	S094_44211	STL	8995501	27096	8968405	0.996987828	0.960540587
SAMN29636416	S095	S095_44232	STL	2372322	728	2371594	0.999693128	0.862975282
SAMN29636417	S095	S095_44238	STL	8466897	17874	8449023	0.997888955	0.837227807
SAMN29636418	S096	S096_44224	STL	10253267	1066556	9186711	0.895978911	0.632176412
SAMN29636419	S097	S097_44222	STL	434520	274	434246	0.999369419	0.377765138
SAMN29636420	S097	S097_44238	STL	13194925	28336	13166589	0.997852508	0.995798304
SAMN29636421	S097	S097_44244	STL	6690312	13562	6676750	0.99797289	0.980903134
SAMN29636422	S097	S097_44256	STL	2455672	14567	2441105	0.994068019	0.996270541
SAMN29636423	S098	S098_44230	STL	1721452	8795	1712657	0.994890941	0.991122566
SAMN29636424	S098	S098_44237	STL	7575156	35752	7539404	0.995280361	0.956226381
SAMN29636425	S099	S099_44230	STL	1521170	105	1521065	0.999930974	0.78393757
SAMN29636426	S099	S099_44242	STL	7009527	7773	7001754	0.998891081	0.913240882
SAMN29636427	S099	S099_44259	STL	5915236	893	5914343	0.999849034	0.742788337
SAMN29636428	S099	S099_44266	STL	7134756	1431	7133325	0.999799433	0.80769557
SAMN29636429	S099	S099_44278	STL	2001132	150	2000982	0.999925042	0.895321397
SAMN29636430	S099	S099_44285	STL	3111882	446	3111436	0.999856678	0.844154596
SAMN29636431	S099	S099_44295	STL	4480701	889	4479812	0.999801594	0.843729603
SAMN29636432	S100	S100_44225	STL	9628426	7016	9621410	0.999271324	0.76765578
SAMN29636433	S100	S100_44243	STL	17215361	19998	17195363	0.998838363	0.984038662
SAMN29636434	S101	S101_44230	STL	8553474	4797	8548677	0.999439175	0.71376027
SAMN29636435	S102	S102_44244	STL	6795130	199542	6595588	0.970634557	0.574825171
SAMN29636436	S103	S103_44238	STL	2487496	3033	2484463	0.998780702	0.86551621
SAMN29636437	S103	S103_44243	STL	6660103	8002	6652101	0.998798517	0.763369047
SAMN29636438	S104	S104_44271	STL	16538976	3053	16535923	0.999815406	0.507072148
SAMN29636439	S104	S104_44281	STL	1674778	536	1674242	0.999679958	0.613941712
SAMN29636440	S104	S104_44288	STL	2406316	203	2406113	0.999915639	0.782665236
SAMN29636441	S104	S104_44291	STL	20768451	760	20767691	0.999963406	0.744427919
SAMN29636442	S104	S104_44299	STL	16390658	611	16390047	0.999962723	0.779655421
SAMN29636443	S105	S105_44281	STL	6152878	6095097	57781	0.00939089	0.703120403
SAMN29636444	S105	S105_44287	STL	1942359	3627	1938732	0.998132683	0.952110452
SAMN29636445	S106	S106_44257	STL	34329309	121264	34208045	0.996467625	0.789165853
SAMN29636446	S106	S106_44258	STL	8457242	15469	8441773	0.998170917	0.712108937
SAMN29636447	S107	S107_44271	STL	7662877	2783	7660094	0.99963682	0.827045073
SAMN29636448	S108	S108_44252	STL	4405024	57925	4347099	0.986850242	0.953850602
SAMN29636449	S109	S109_44246	STL	5510198	152416	5357782	0.972339288	0.958238876
SAMN29636450	S109	S109_44257	STL	11037171	619	11036552	0.999943917	0.958213036
SAMN29636451	S109	S109_44258	STL	2185106	44477	2140629	0.979645381	0.952125287
SAMN29636452	S109	S109_44275	STL	9753057	328534	9424523	0.966314767	0.971850459
SAMN29636453	S110	S110_44251	STL	1360188	337990	1022198	0.751512291	0.861624656
SAMN29636454	S110	S110_44263	STL	904912	28725	876187	0.968256582	0.86929046
SAMN29636455	S111	S111_44282	STL	3151905	266043	2885862	0.915592951	0.992314601
SAMN29636456	S111	S111_44285	STL	10802916	4054	10798862	0.999624731	0.991210555
SAMN29636457	S111	S111_44286	STL	33179571	7490	33172081	0.999774259	0.990484709
SAMN29636458	S112	S112_44262	STL	5689480	5684098	5382	9.46E-04	0.638610182
SAMN29636459	S112	S112_44267	STL	3463087	3459915	3172	9.16E-04	0.592055485
SAMN29636460	S113	S113_44271	STL	1830919	151054	1679865	0.917498262	0.990679013
SAMN29636461	S113	S113_44281	STL	3541786	101551	3440235	0.971327743	0.973159392
SAMN29636462	S114	S114_44250	STL	1884015	1777556	106459	0.05650645	0.876262223
SAMN29636463	S114	S114_44253	STL	2502804	714353	1788451	0.714578928	0.915889784
SAMN29636464	S115	S115_44274	STL	4969089	1368250	3600839	0.724647717	0.92312014
SAMN29636465	S116	S116_44288	STL	3406698	6524	3400174	0.998084949	0.835430481
SAMN29636466	S117	S117_44280	STL	12160607	56577	12104030	0.995347518	0.756746224
SAMN29636467	S117	S117_44303	STL	7550485	2501	7547984	0.999668763	0.820455369
SAMN29636468	S118	S118_44314	STL	10235615	38712	10196903	0.996217912	0.985453426

SAMN29636469	S118	S118_44322	STL	5523312	10293	5513019	0.998136444	0.976423263
SAMN29636470	S119	S119_44295	STL	7471146	6992242	478904	0.064100474	0.406795516
SAMN29636471	S120	S120_44285	STL	3776656	335	3776321	0.999911297	0.677462271
SAMN29636472	S120	S120_44308	STL	1633861	1054	1632807	0.999354902	0.606692646
SAMN29636473	S121	S121_44291	STL	5679918	681	5679237	0.999880104	0.751490033
SAMN29636474	S121	S121_44300	STL	3943304	1337	3941967	0.999660944	0.573190745
SAMN29636475	S122	S122_44288	STL	2839268	2677	2836591	0.999057151	0.979842706
SAMN29636476	S123	S123_44302	STL	3000878	70348	2930530	0.976557527	0.657138129
SAMN29636477	S123	S123_44309	STL	7706883	61476	7645407	0.992023234	0.979977783
SAMN29636478	S124	S124_44301	STL	32527475	2297070	30230405	0.929380624	0.979668516
SAMN29636479	S125	S125_44287	STL	14633529	43659	14589870	0.997016509	0.853004173
SAMN29636480	S126	S126_44298	STL	4683218	1626	4681592	0.999652803	0.783145135
SAMN29636481	S126	S126_44307	STL	13600101	3243	13596858	0.999761546	0.634107895
SAMN29636482	S127	S127_44301	STL	7522164	159754	7362410	0.978762229	0.418036078
SAMN29636483	S128	S128_44309	STL	6188882	1564	6187318	0.999747289	0.492064413
SAMN29636484	S128	S128_44316	STL	3623708	44324	3579384	0.98776833	0.941005491
SAMN29636485	S129	S129_44315	STL	16029133	22221	16006912	0.998613712	0.854864136
SAMN29636486	S130	S130_44309	STL	17055551	16551	17039000	0.999029583	0.916537532
SAMN29636487	S131	S131_44310	STL	13505578	93061	13412517	0.99310944	0.800833729
SAMN29636488	S131	S131_44319	STL	6521697	4884460	1637237	0.251044628	0.996341397
SAMN29636489	S132	S132_44336	STL	3864412	3603711	260701	0.06746201	0.558766556
SAMN29636490	S133	S133_44321	STL	21588024	15767	21572257	0.999269641	0.803691519
SAMN29636491	S134	S134_44323	STL	10434097	5603	10428494	0.999463011	0.758097478
SAMN29636492	S135	S135_44344	STL	6193373	636350	5557023	0.89725308	0.657653207
SAMN29636493	S136	S136_44341	STL	5836789	61723	5775066	0.989425179	0.171420032
SAMN29636494	S137	S137_44343	STL	4152497	3283	4149214	0.999209391	0.877751786
SAMN29636495	S138	S138_44343	STL	7360312	957	7359355	0.999869978	0.57551674
SAMN29636496	S139	S139_44349	STL	7309309	9494	7299815	0.998701108	0.6420486
SAMN29636594	S001	S001_001	STL	1484130	1449681	34449	0.023211579	0.960173009
SAMN29636595	S002	S002_002	STL	1723949	175550	1548399	0.898169841	0.740964054
SAMN29636596	S002	S002_003	STL	2501105	13721	2487384	0.994514025	0.944214082
SAMN29636597	S003	S003_004	STL	1916373	1552378	363995	0.189939537	0.938850808
SAMN29636598	S003	S003_005	STL	3040704	90805	2949899	0.97013685	0.994457437
SAMN29636599	S003	S003_006	STL	1319243	56540	1262703	0.957142088	0.992850259
SAMN29636600	S003	S003_007	STL	2588044	118203	2469841	0.954327283	0.994548232
SAMN29636601	S004	S004_008	STL	2248698	4042	2244656	0.998202515	0.779440591
SAMN29636602	S005	S005_009	STL	2003530	69277	1934253	0.965422529	0.990702871
SAMN29636603	S005	S005_010	STL	816824	67330	749494	0.917570982	0.981567564
SAMN29636604	S006	S006_011	STL	1497125	4805	1492320	0.996790515	0.69127801
SAMN29636605	S007	S007_012	STL	2233389	723388	1510001	0.676102999	0.698266425
SAMN29636606	S008	S008_013	STL	1776401	2921	1773480	0.998355664	0.731746059
SAMN29636607	S008	S008_014	STL	3488920	12135	3476785	0.996521846	0.529509302
SAMN29636608	S009	S009_015	STL	2028602	156009	1872593	0.923095314	0.933642281
SAMN29636609	S009	S009_016	STL	2356226	94553	2261673	0.959870997	0.935299665
SAMN29636610	S009	S009_017	STL	1769570	75007	1694563	0.957612866	0.93225333
SAMN29636611	S010	S010_018	STL	404674	6699	397975	0.983445934	0.87681387
SAMN29636612	S010	S010_019	STL	2342766	27966	2314800	0.988062828	0.963724296
SAMN29636613	S011	S011_020	STL	2921150	1135	2920015	0.999611454	0.757727272
SAMN29636614	S012	S012_021	STL	1102453	919291	183162	0.166140416	0.81246656
SAMN29636615	S013	S013_022	STL	2922291	5954	2916337	0.997962557	0.885623644
SAMN29636616	S014	S014_023	STL	102651	33028	69623	0.678249603	0.971417491
SAMN29636617	S014	S014_024	STL	4603536	890865	3712671	0.806482452	0.977154722
SAMN29636618	S015	S015_025	STL	2675148	707	2674441	0.999735716	0.427548037
SAMN29636619	S015	S015_026	STL	1786930	648	1786282	0.999637367	0.43671268
SAMN29636620	S015	S015_027	STL	1621582	372	1621210	0.999770594	0.973712227
SAMN29636621	S016	S016_028	STL	2158842	1630	2157212	0.999244966	0.715812818
SAMN29636622	S016	S016_029	STL	2290136	669	2289467	0.999707878	0.861174238
SAMN29636623	S016	S016_030	STL	1423367	444	1422923	0.999688064	0.849156982
SAMN29636624	S017	S017_031	STL	688845	684474	4371	0.006345404	0.819492107
SAMN29636625	S018	S018_032	STL	2149816	29385	2120431	0.986331388	0.763906017
SAMN29636626	S018	S018_033	STL	2704023	301229	2402794	0.88859969	0.923304703
SAMN29636627	S018	S018_034	STL	2287764	10929	2276835	0.995222846	0.610272593
SAMN29636628	S018	S018_035	STL	1980989	5320	1975669	0.997314473	0.609390034
SAMN29636629	S018	S018_036	STL	1345094	491	1344603	0.99963497	0.784778853
SAMN29636630	S019	S019_037	STL	1886886	1429	1885457	0.999242668	0.81131365
SAMN29636631	S020	S020_038	STL	1144277	1110250	34027	0.029736681	0.977047639
SAMN29636632	S021	S021_039	STL	2160953	1419388	741565	0.343165724	0.971863559
SAMN29636633	S021	S021_040	STL	614384	26288	588096	0.957212427	0.943249061
SAMN29636634	S021	S021_041	STL	2006940	1016	2005924	0.999493757	0.995664841
SAMN29636635	S022	S022_042	STL	1053960	660	1053300	0.99937379	0.995177063

SAMN29636636	S022	S022_043	STL	254075	82926	171149	0.673616058	0.913099112
SAMN29636637	S023	S023_044	STL	2898308	10407	2887901	0.996409284	0.851733837
SAMN29636638	S023	S023_045	STL	2167647	534	2167113	0.99975365	0.80157103
SAMN29636639	S023	S023_046	STL	1448126	2471	1445655	0.998293657	0.828243945
SAMN29636640	S024	S024_047	STL	855864	20574	835290	0.975961134	0.936753702
SAMN29636641	S024	S024_048	STL	3561863	1245	3560618	0.999650464	0.922580574
SAMN29636642	S024	S024_049	STL	733412	278	733134	0.99962095	0.952701689
SAMN29636643	S024	S024_050	STL	1542790	1093	1541697	0.999291543	0.926953221
SAMN29636644	S024	S024_051	STL	1863973	500	1863473	0.999731756	0.823259044
SAMN29636645	S024	S024_052	STL	3161143	1268	3159875	0.999598879	0.863760434
SAMN29636646	S024	S024_053	STL	1877796	1109	1876687	0.999409414	0.548652492
SAMN29636562	S025	S025_005	STL	2148182	934	2147248	0.999565214	0.904141953
SAMN29636647	S025	S025_054	STL	3842755	5199	3837556	0.998647064	0.905466656
SAMN29636648	S026	S026_055	STL	2076159	252299	1823860	0.878477997	0.680020396
SAMN29636558	S027	S027_001	STL	1327052	1434	1325618	0.998919409	0.993166961
SAMN29636559	S027	S027_002	STL	1452220	1070	1451150	0.999263197	0.987142611
SAMN29636560	S027	S027_003	STL	1738790	1648	1737142	0.999052214	0.771364114
SAMN29636563	S027	S027_006	STL	547329	959	546370	0.998247855	0.990861504
SAMN29636649	S027	S027_056	STL	1474958	525	1474433	0.999644058	0.992447944
SAMN29636650	S027	S027_057	STL	1392774	252	1392522	0.999819066	0.993820564
SAMN29636651	S027	S027_058	STL	4006487	642	4005845	0.99983976	0.986084834
SAMN29636561	S028	S028_004	STL	1624227	42541	1581686	0.973808464	0.88329542
SAMN29636564	S029	S029_007	STL	2832595	2280	2830315	0.999195084	0.899277642
SAMN29636565	S029	S029_008	STL	6468641	3478	6465163	0.999462329	0.913870076
SAMN29636497	S030	S030_42821	STL	16046398	10401	16035997	0.999351817	0.730227749
SAMN29636498	S031	S031_42811	STL	16976114	862283	16113831	0.949206102	0.64237021
SAMN29636499	S032	S032_42852	STL	5376274	686	5375588	0.999872402	0.99068753
SAMN29636500	S033	S033_42892	STL	326	6	320	0.981595092	0.475
SAMN29636501	S034	S034_43236	STL	9687192	3010	9684182	0.99968928	0.447603422
SAMN29636502	S035	S035_43318	STL	8361147	13389	8347758	0.998398665	0.795166319
SAMN29636503	S036	S036_43313	STL	14161634	45052	14116582	0.996818729	0.974705846
SAMN29636504	S036	S036_43318	STL	3806436	2192	3804244	0.999424133	0.978833639
SAMN29636505	S037	S037_43360	STL	5978450	5251804	726646	0.121544213	0.960967239
SAMN29636506	S038	S038_43392	STL	14709231	6756	14702475	0.999540697	0.408610387
SAMN29636507	S039	S039_43438	STL	4252513	269456	3983057	0.936636055	0.992058361
SAMN29636508	S039	S039_43441	STL	1957249	1061526	895723	0.457643866	0.967129347
SAMN29636509	S039	S039_43448	STL	12731342	9726480	3004862	0.236020837	0.667066574
SAMN29636510	S040	S040_43531	STL	12977491	160347	12817144	0.987644222	0.995931153
SAMN29636511	S044	S044_43999	STL	5757864	950	5756914	0.999835008	0.8396872
SAMN29636512	S045	S045_44008	STL	20542224	620	20541604	0.999969818	0.909712163
SAMN29636513	S046	S046_44032	STL	31018188	33398	30984790	0.998923277	0.700234341
SAMN29636514	S047	S047_44046	STL	45885207	10168	45875039	0.999778404	0.981025716
SAMN29636515	S047	S047_44050	STL	20888902	22266	20866636	0.998934075	0.806681297
SAMN29636516	S048	S048_44076	STL	2587341	299	2587042	0.999884437	0.935381026
SAMN29636517	S048	S048_44085	STL	6455303	3471	6451832	0.999462303	0.937981336
SAMN29636518	S048	S048_44088	STL	9728184	52324	9675860	0.994621401	0.76346423
SAMN29636519	S048	S048_44090	STL	18025647	1350	18024297	0.999925107	0.902419495
SAMN29636520	S048	S048_44095	STL	7774752	2140	7772612	0.99972475	0.774165107
SAMN29636521	S049	S049_44071	STL	8880903	10359	8870544	0.998833565	0.394465323
SAMN29636522	S050	S050_44076	STL	14046071	27826	14018245	0.998018948	0.635980467
SAMN29636523	S050	S050_44078	STL	12988266	37065	12951201	0.99714627	0.615929364
SAMN29636524	S050	S050_44085	STL	7229513	2698	7226815	0.999626808	0.806007764
SAMN29636525	S050	S050_44088	STL	9411493	719	9410774	0.999923604	0.842256439
SAMN29636526	S051	S051_44123	STL	12324886	9558	12315328	0.999224496	0.890662595
SAMN29636527	S051	S051_44125	STL	4591968	6217	4585751	0.998646114	0.924997454
SAMN29636528	S051	S051_44127	STL	9824981	17149	9807832	0.998254551	0.827408952
SAMN29636529	S051	S051_44130	STL	20421535	2423	20419112	0.999881351	0.820889322
SAMN29636530	S051	S051_44137	STL	27025395	969	27024426	0.999964145	0.862952538
SAMN29636531	S051	S051_44144	STL	12205620	1783766	10421854	0.853856994	0.770919646
SAMN29636532	S051	S051_44146	STL	313	95	218	0.696485623	0.47706422
SAMN29636533	S052	S052_44095	STL	10712699	39009	10673690	0.996358621	0.709493718
SAMN29636534	S052	S052_44106	STL	17148933	36135	17112798	0.997892872	0.653567874
SAMN29636535	S053	S053_44109	STL	15428108	4922	15423186	0.999680972	0.871450685
SAMN29636536	S054	S054_44134	STL	6939762	1118	6938644	0.999838899	0.83003927
SAMN29636537	S055	S055_44125	STL	22065194	77952	21987242	0.996467196	0.785089098
SAMN29636538	S055	S055_44127	STL	10145032	5022	10140010	0.999504979	0.76890506
SAMN29636539	S055	S055_44130	STL	7583558	2736	7580822	0.999639219	0.75708373
SAMN29636540	S055	S055_44137	STL	13135767	887	13134880	0.999932474	0.943901429
SAMN29636541	S055	S055_44139	STL	14767388	233	14767155	0.999984222	0.975285896
SAMN29636542	S056	S056_44116	STL	6026670	1268225	4758445	0.789564552	0.859819962

SAMN29636543	S056	S056_44120	STL	15592253	49519	15542734	0.996824128	0.881533583
SAMN29636544	S056	S056_44123	STL	6405136	1439	6403697	0.999775337	0.924967874
SAMN29636545	S056	S056_44127	STL	10349653	5479	10344174	0.99947061	0.896816991
SAMN29636546	S056	S056_44130	STL	7813474	47967	7765507	0.993860989	0.8863693
SAMN29636547	S056	S056_44144	STL	4878513	7544	4870969	0.998453627	0.815756577
SAMN29636548	S057	S057_44118	STL	3615422	2969	3612453	0.999178796	0.946881523
SAMN29636549	S058	S058_44148	STL	11048163	1968	11046195	0.999821871	0.795904563
SAMN29636550	S059	S059_44155	STL	14146685	420930	13725755	0.970245326	0.972888777
SAMN29636551	S059	S059_44158	STL	2795786	122544	2673242	0.956168319	0.967816232
SAMN29636552	S059	S059_44160	STL	7500412	1599849	5900563	0.786698517	0.972377551
SAMN29636553	S059	S059_44169	STL	16866038	1541184	15324854	0.908622049	0.958231054
SAMN29636554	S059	S059_44172	STL	7642916	566790	7076126	0.925841132	0.982303594
SAMN29636555	S059	S059_44173	STL	285986	51	285935	0.99982167	0.833140399
SAMN29636556	S059	S059_44209	STL	25995595	3701	25991894	0.99985763	0.969829517
SAMN29636557	S060	S060_44328	STL	6449896	2071102	4378794	0.678893737	0.99397688
SAMN29636566	S061	S061_43242	STL	7993553	5494890	2498663	0.312584779	0.90000092
SAMN29636567	S062	S062_43291	STL	11121353	2225586	8895767	0.799881723	0.778584691
SAMN29636568	S063	S063_43292	STL	5471799	4871	5466928	0.999109799	0.868975959
SAMN29636569	S064	S064_43327	STL	9850398	3982696	5867702	0.595681718	0.743861907
SAMN29636570	S065	S065_43346	STL	9282111	2028	9280083	0.999781515	0.935645403
SAMN29636571	S065	S065_43354	STL	13472862	2447	13470415	0.999818376	0.942251742
SAMN29636572	S066	S066_43361	STL	13130863	68852	13062011	0.994756476	0.920690696
SAMN29636573	S067	S067_43374	STL	2044974	1833779	211195	0.103275152	0.92552854
SAMN29636574	S068	S068_43389	STL	5362082	24037	5338045	0.995517226	0.987933597
SAMN29636575	S069	S069_43390	STL	8003903	39313	7964590	0.995088271	0.990481117
SAMN29636576	S070	S070_43399	STL	10010967	535030	9475937	0.946555612	0.38980103
SAMN29636577	S071	S071_43412	STL	5565146	5516370	48776	0.00876455	0.883426275
SAMN29636578	S072	S072_43413	STL	11181694	1758	11179936	0.999842779	0.683748816
SAMN29636579	S073	S073_43445	STL	5265330	854929	4410401	0.8376305	0.213433427
SAMN29636580	S074	S074_43495	STL	4992158	171004	4821154	0.965745475	0.993855828
SAMN29636581	S075	S075_43495	STL	20380425	5489	20374936	0.999730673	0.675571153
SAMN29636582	S076	S076_43514	STL	10051278	23209	10028069	0.99769094	0.892117715
SAMN29636583	S077	S077_43514	STL	9302200	5672	9296528	0.999390252	0.298363109
SAMN29636584	S078	S078_43515	STL	8063130	874858	7188272	0.891498959	0.939844931
SAMN29636585	S079	S079_43543	STL	7016709	53061	6963648	0.992437908	0.930242741
SAMN29636586	S080	S080_43543	STL	12105610	158657	11946953	0.986893928	0.913249512
SAMN29636587	S081	S081_43559	STL	2311405	557	2310848	0.999759021	0.876370925
SAMN29636588	S082	S082_43613	STL	8446151	4739	8441412	0.999438916	0.819264834
SAMN29636589	S083	S083_43616	STL	25616574	3093	25613481	0.999879258	0.866692231
SAMN29636590	S084	S084_43691	STL	6567344	30135	6537209	0.995411387	0.812255199
SAMN29636591	S085	S085_43864	STL	9281830	3260	9278570	0.999648776	0.775570697
SAMN29636592	S086	S086_43881	STL	9917297	246027	9671270	0.975192131	0.996030304
SAMN29636593	S087	S087_44092	STL	9656825	358984	9297841	0.962825877	0.764958661
SRR1031102	SRR1031102	SRR1031102	STL	-	-	-	-	-
SRR1031154	SRR1031154	SRR1031154	STL	-	-	-	-	-
SRR1564387	SRR1564387	SRR1564387	STL	-	-	-	-	-
SRR1565914	SRR1565914	SRR1565914	STL	-	-	-	-	-
SRR1803355	SRR1803355	SRR1803355	STL	-	-	-	-	-
SRR1803358	SRR1803358	SRR1803358	STL	-	-	-	-	-
SRR1803862	SRR1803862	SRR1803862	STL	-	-	-	-	-
SRR1803892	SRR1803892	SRR1803892	STL	-	-	-	-	-
SRR1803903	SRR1803903	SRR1803903	STL	-	-	-	-	-
SRR1804009	SRR1804009	SRR1804009	STL	-	-	-	-	-
SRR1804055	SRR1804055	SRR1804055	STL	-	-	-	-	-
SRR1804086	SRR1804086	SRR1804086	STL	-	-	-	-	-
SRR1804107	SRR1804107	SRR1804107	STL	-	-	-	-	-
SRR1804119	SRR1804119	SRR1804119	STL	-	-	-	-	-
SRR1804148	SRR1804148	SRR1804148	STL	-	-	-	-	-
SRR1804203	SRR1804203	SRR1804203	STL	-	-	-	-	-
SRR1804286	SRR1804286	SRR1804286	STL	-	-	-	-	-
SRR1804539	SRR1804539	SRR1804539	STL	-	-	-	-	-
SRR1804618	SRR1804618	SRR1804618	STL	-	-	-	-	-
SRR1804648	SRR1804648	SRR1804648	STL	-	-	-	-	-
SRR1804676	SRR1804676	SRR1804676	STL	-	-	-	-	-
SRR1804688	SRR1804688	SRR1804688	STL	-	-	-	-	-
SRR1804756	SRR1804756	SRR1804756	STL	-	-	-	-	-
SRR512768	SRR512768	SRR512768	STL	-	-	-	-	-
SRR514196	SRR514196	SRR514196	STL	-	-	-	-	-
SRR532163	SRR532163	SRR532163	STL	-	-	-	-	-
