# Supplementary material for: Tracking gut microbiome and bloodstream infection in critically ill adults
Source: PLoS One. 2023 Oct 10;18(10):e0289923. doi: 10.1371/journal.pone.0289923 (PMC10564172; doi:10.1371/journal.pone.0289923)
Supplement: S3 Table — (PDF) [file pone.0289923.s004.pdf]

Table S3. Whole genome sequence assembled from blood culture isolates.

Twenty-five blood culture isolates were subjected to WGS, which includes 23 unique BSI episodes (one E. faecalis BSI was subjected to WGS in three serial positive blood cultures).

Accesssion	Bin Id	Organism	Completeness	Contamination	Strain heterogeneity	Genome size (bp)	# contigs	N50 (contigs)	Mean contig length (bp)	Longest contig (bp)	GC	Depth
SAMN29017764	S130_1	Achromobacter insolitus	92.3	1.4	0	6068322	720	12197	8428	113836	64.7	201.50
SAMN29017754	S101_1	Bacteroides ovatus	99.26	0	0	5310990	2	5277274	2655495	5277274	43.2	41.94
-	S082_1	Candida albicans	-	-	-	9329155	4875	2045	1913	21196	33.9	14.17
SAMN29017778	S059_1	Enterococcus faecalis	99.53	0.37	0	3243856	199	74874	16300	167826	37.2	52.63
SAMN29017753	S099_1	Enterococcus faecalis	99.16	0.37	0	3097254	265	27609	11687	84506	37.4	58.32
SAMN29017760	S127_1	Enterococcus faecalis	99.63	0	0	2707870	142	33960	19069	87305	37.7	19.78
SAMN29017761	S127_2	Enterococcus faecalis	99.63	0	0	2717664	130	36409	20905	79026	37.6	56.27
SAMN29017762	S127_3	Enterococcus faecalis	99.63	0	0	2739774	80	70540	34247	166515	37.5	320.11
SAMN29017773	S139_1	Enterococcus faecalis	99.63	0.37	0	2707031	111	38795	24387	84336	37.7	48.69
SAMN29017765	S132_1	Enterococcus gallinarum	99.25	0.57	0	3396116	78	123125	43539	259560	40.4	97.66
SAMN29017777	S055_1	Escherichia coli	99.67	0.24	0	5219644	343	116963	15217	279776	50.7	31.50
SAMN29017783	S083_1	Escherichia coli	99.97	0.32	14.29	5253887	244	57137	21532	262052	50.5	33.03
SAMN29017774	S051_1	Klebsiella pneumoniae	98.66	0.12	0	5096264	223	35644	22853	141660	57.5	74.85
SAMN29017780	S059_3	Klebsiella pneumoniae	99.9	0.03	0	5680364	276	87456	20581	262051	57.1	40.11
SAMN29017770	S135_1	Klebsiella pneumoniae	99.64	0.34	0	5477913	141	84135	38850	262825	57.1	34.02
SAMN29017781	S078_1	Morganella morganii	100	0.03	0	3798406	60	226244	63306	390970	51.1	46.10
SAMN29017755	S104_1	Proteus mirabilis	100	0.08	0	3920890	357	20426	10982	76611	39.4	92.62
SAMN29017763	S129_1	Pseudomonas aeruginosa	94.18	0.62	0	5800952	793	11416	7315	59956	65.8	118.89
SAMN29017767	S134_1	Pseudomonas aeruginosa	88.98	0.85	0	5098874	871	8657	5854	48968	65.4	26.10
SAMN29017772	S137_1	Pseudomonas aeruginosa	73.38	0.57	0	4556470	580	12451	7855	91530	64	48.20
SAMN29017758	S117_1	Serratia marcescens	99.13	0.15	0	5086323	330	25949	15413	91865	59.8	197.11
SAMN29017766	S133_1	Staphylococcus aureus	98.65	0.28	0	2652869	372	11610	7131	54456	33.1	262.10
SAMN29017771	S136_1	Staphylococcus aureus	97.8	0.76	0	2578543	302	12597	8538	76981	33	64.39
SAMN29017768	S134_2	Staphylococcus haemolyticus	96.45	0.57	0	2345600	385	9927	6092	40455	33	81.76
SAMN29017782	S081_1	Streptococcus pyogenes	99.85	0	0	1821535	35	115206	52043	442723	38.1	52.73

S082_1 does not have a genBank accession number due to incompleteness of the genome and will have raw sequence data uploaded to SRA

S082_1 does not have a Completeness, Contamination or Strain heterogeneity value because these results are from checkM and checkM only works on prokaryotic genomes
