# Supplementary material for: Tracking gut microbiome and bloodstream infection in critically ill adults
Source: PLoS One. 2023 Oct 10;18(10):e0289923. doi: 10.1371/journal.pone.0289923 (PMC10564172; doi:10.1371/journal.pone.0289923)
Supplement: S4 Table — (PDF) [file pone.0289923.s005.pdf]

Table S4. Reference genomes used for analysis

Bin Id	Accession	Organism	Completeness	Contamination	Strain heterogeneity	Genome size (bp)	# contigs	N50 (contigs)	Mean contig length (bp)	Longest contig (bp)	GC
GCF_000165835.1_ASM16583v1_genomic	GCF_000165835.1	Achromobacter xylosoxidans	99.53	0.03	0	7359146	3	7013095	2453048	7013095	66
GCF_000508285.1_ASM50828v1_genomic	GCF_000508285.1	Achromobacter xylosoxidans	99.53	0.58	0	6683584	1	6683583	6683583	6683583	68
GCF_000783435.2_ASM78343v2_genomic	GCF_000783435.2	Achromobacter insolitus	99.53	0.47	0	6893045	2	6523893	3446522	6523893	65
GCF_000967095.2_A.x.NH44784-1996_genomic	GCF_000967095.2	Achromobacter xylosoxidans	99.53	0.93	0	6916670	16	627136	431445	2591805	67
GCF_001051055.1_ASM105105v1_genomic	GCF_001051055.1	Achromobacter xylosoxidans	99.07	0	0	5876049	1	5876049	5876049	5876049	68
GCF_001639685.1_ASM163968v1_genomic	GCF_001639685.1	Achromobacter insolitus	99.53	0.47	0	6515216	58	554927	112329	1211020	65
GCF_001641515.1_ASM164151v1_genomic	GCF_001641515.1	Achromobacter insolitus	100	0.47	0	6738489	172	117992	39177	341890	65
GCF_001971645.1_ASM197164v1_genomic	GCF_001971645.1	Achromobacter insolitus	98.91	0.47	0	6492697	1	6492697	6492697	6492697	65
GCF_002209555.1_ASM220955v1_genomic	GCF_002209555.1	Achromobacter insolitus	99.53	0.47	0	6444435	29	1063361	222221	1212004	65
GCF_003293535.1_ASM329353v1_genomic	GCF_003293535.1	Achromobacter insolitus	99.53	0.47	0	6438278	18	725996	357580	1206205	65
GCF_008245125.1_ASM824512v1_genomic	GCF_008245125.1	Achromobacter insolitus	99.44	0.47	0	6428890	1	6428890	6428890	6428890	65
GCF_008432465.1_ASM843246v1_genomic	GCF_008432465.1	Achromobacter xylosoxidans	99.53	0.47	0	6402982	1	6402982	6402982	6402982	68
GCF_009363015.1_ASM936301v1_genomic	GCF_009363015.1	Achromobacter xylosoxidans	99.53	0.47	0	6222121	2	5943204	3111060	5943204	66
GCF_011063385.1_ASM1106338v1_genomic	GCF_011063385.1	Achromobacter insolitus	99.53	0.47	0	6438429	53	333164	121461	717244	65
GCF_013282235.1_ASM1328223v1_genomic	GCF_013282235.1	Achromobacter xylosoxidans	99.53	0.47	0	6452474	1	6452474	6452474	6452474	68
GCF_014490035.1_ASM1449003v1_genomic	GCF_014490035.1	Achromobacter xylosoxidans	99.44	0	0	6761376	1	6761376	6761376	6761376	66
GCF_022325125.1_ASM2232512v1_genomic	GCF_022325125.1	Achromobacter insolitus	99.53	0.47	0	6380249	67	462186	95224	646916	65
GCF_900009115.1_BN2877_genomic	GCF_900009115.1	Achromobacter xylosoxidans	99.53	0.47	0	6501194	1	6501194	6501194	6501194	68
GCF_902860205.1_LMG_6001_genomic	GCF_902860205.1	Achromobacter insolitus	99.53	0.47	0	6320373	36	568051	175565	1134874	65
GCF_000391485.2_ASM39148v2_genomic	GCF_000391485.2	Enterococcus faecalis	99.53	0.19	0	3267721	3	3152103	1089240	3152103	37
GCF_000550745.1_ASM55074v1_genomic	GCF_000550745.1	Enterococcus faecalis	99.63	0.19	0	2961043	1	2961043	2961043	2961043	38
GCF_001989555.1_ASM198955v1_genomic	GCF_001989555.1	Enterococcus faecalis	99.63	0.37	0	3243539	3	3111017	1081179	3111017	37
GCF_002208945.1_ASM220894v2_genomic	GCF_002208945.1	Enterococcus faecalis	99.63	0	0	2861022	1	2861022	2861022	2861022	38
GCF_003966385.1_ASM396638v1_genomic	GCF_003966385.1	Enterococcus faecalis	99.63	0.75	0	3138474	4	2893216	784618	2893216	37
GCF_006349345.1_ASM634934v1_genomic	GCF_006349345.1	Enterococcus faecalis	99.63	0.09	0	2955738	2	2881081	1477869	2881081	37
GCF_007632055.1_ASM763205v1_genomic	GCF_007632055.1	Enterococcus faecalis	98.72	0	0	2695820	287	25508	9293	113017	38
GCF_009662495.1_ASM966249v1_genomic	GCF_009662495.1	Enterococcus faecalis	99.53	0.56	0	3117418	3	2944359	1039139	2944359	37
GCF_010103655.1_ASM1010365v1_genomic	GCF_010103655.1	Enterococcus faecalis	99.63	0	0	2933718	1	2933718	2933718	2933718	38
GCF_012594215.1_ASM1259421v1_genomic	GCF_012594215.1	Enterococcus faecalis	99.63	0	0	2993403	1	2993403	2993403	2993403	37
GCF_014893535.1_ASM1489353v1_genomic	GCF_014893535.1	Enterococcus faecalis	98.5	0	0	2940017	251	64792	10610	204291	38
GCF_017639585.1_ASM1763958v1_genomic	GCF_017639585.1	Enterococcus faecalis	99.25	0.37	0	2806628	3	2754417	935542	2754417	38
GCF_018517125.1_ASM1851712v1_genomic	GCF_018517125.1	Enterococcus faecalis	99.63	0	0	3097453	3	2991600	1032484	2991600	38
GCF_018986755.2_ASM1898675v2_genomic	GCF_018986755.2	Enterococcus faecalis	99.63	0.56	0	3485032	7	3264821	497861	3264821	37
GCF_902164645.1_25426_7_121_genomic	GCF_902164645.1	Enterococcus faecalis	97.75	0	0	3380663	114	49852	23004	153985	38
GCF_000166535.2_ASM16653v3_genomic	GCF_000166535.2	Escherichia coli	99.97	0.84	16.67	5094491	37	4870513	137688	4870513	51
GCF_001938625.2_ASM193862v2_genomic	GCF_001938625.2	Escherichia coli	99.97	0.05	0	5439104	7	5060620	776984	5060620	51
GCF_002125925.1_ASM212592v1_genomic	GCF_002125925.1	Escherichia coli	99.67	0.74	62.5	4863033	2	4771482	2431466	4771482	51
GCF_002634895.1_ASM263489v1_genomic	GCF_002634895.1	Escherichia coli	99.67	0.14	0	5059202	5	4897877	1011840	4897877	51
GCF_002736085.1_ASM273608v1_genomic	GCF_002736085.1	Escherichia coli	99.93	0.18	0	5017436	23	710110	218066	1309910	51
GCF_002879735.1_ASM287973v1_genomic	GCF_002879735.1	Escherichia coli	99.97	0.06	0	4985704	2	4824922	2492752	4824922	51
GCF_003017885.1_ASM301788v1_genomic	GCF_003017885.1	Escherichia coli	99.93	0.55	0	5626021	3	5394679	1875307	5394679	51
GCF_003028695.1_ASM302869v1_genomic	GCF_003028695.1	Escherichia coli	99.97	0.33	0	4989876	47	211807	106067	621047	51
GCF_003072445.1_ASM307244v1_genomic	GCF_003072445.1	Escherichia coli	99.93	0.14	0	5500653	3	5299162	1833481	5299162	51
GCF_003112045.1_ASM311204v1_genomic	GCF_003112045.1	Escherichia coli	99.93	0.35	14.29	5166836	4	4742904	1291684	4742904	50
GCF_003194125.1_ASM319412v1_genomic	GCF_003194125.1	Escherichia coli	99.87	0.45	12.5	5896485	6	5219446	982730	5219446	50
GCF_003367575.1_ASM336757v1_genomic	GCF_003367575.1	Escherichia coli	99.93	0.82	0	4938383	2	4689476	2469141	4689476	51
GCF_003666405.1_ASM366640v1_genomic	GCF_003666405.1	Escherichia coli	99.93	0.56	0	5222791	8	4864848	652836	4864848	51
GCF_003999775.1_ASM399977v1_genomic	GCF_003999775.1	Escherichia coli	99.97	0.33	0	5199428	66	207446	78702	544102	51
GCF_004116595.1_ASM411659v1_genomic	GCF_004116595.1	Escherichia coli	99.93	0.42	0	5038603	8	1107219	629769	2205881	51
GCF_004138625.1_ASM413862v1_genomic	GCF_004138625.1	Escherichia coli	99.93	0.27	0	5083560	1	5083460	5083460	5083460	51
GCF_004358945.1_ASM435894v1_genomic	GCF_004358945.1	Escherichia coli	99.63	0.19	0	4592928	6	1395506	765404	1765655	51
GCF_006514375.1_ASM651437v1_genomic	GCF_006514375.1	Escherichia coli	99.82	0.09	0	5522685	133	318816	40710	1018691	51
GCF_007107705.1_ASM710770v1_genomic	GCF_007107705.1	Escherichia coli	99.97	0.11	0	5417941	4	5215159	1354460	5215159	51
GCF_012955465.1_ASM1295546v1_genomic	GCF_012955465.1	Escherichia coli	98.59	3.62	95.65	4937450	23	448479	214661	1179710	51
GCF_013344585.1_ASM1334458v1_genomic	GCF_013344585.1	Escherichia coli	99.97	0.36	0	5192998	7	5015404	741856	5015404	51
GCF_014216355.1_ASM1421635v1_genomic	GCF_014216355.1	Escherichia coli	95.17	0.39	0	5689720	789	14125	6054	435041	51
GCF_014898635.1_ASM1489863v1_genomic	GCF_014898635.1	Escherichia coli	98.79	0.09	0	5133298	401	27926	11102	164796	51
GCF_016801475.1_ASM1680147v1_genomic	GCF_016801475.1	Escherichia coli	99.62	0.1	0	4805003	1	4804903	4804903	4804903	51
GCF_016864435.1_ASM1686443v1_genomic	GCF_016864435.1	Escherichia coli	99.97	0.33	0	5684545	2	5543715	2842172	5543715	51
GCF_018208275.1_ASM1820827v1_genomic	GCF_018208275.1	Escherichia coli	99.25	0.04	0	5127133	233	80094	19104	332595	51
GCF_018986775.1_ASM1898677v1_genomic	GCF_018986775.1	Escherichia coli	99.97	0.38	33.33	5163097	4	4932427	1290749	4932427	51
GCF_019614135.1_ASM1961413v1_genomic	GCF_019614135.1	Escherichia coli	99.55	0.19	25	4820169	95	123813	50640	379066	51
GCF_902164685.1_25426_7_120_genomic	GCF_902164685.1	Escherichia coli	99.67	0.08	0	5132864	49	228858	100519	621057	51
GCF_000240185.1_ASM24018v2_genomic	GCF_000240185.1	Klebsiella pneumoniae	99.65	0.19	0	5682322	7	5333941	811760	5333941	57
GCF_000967845.1_ASM96784v1_genomic	GCF_000967845.1	Klebsiella pneumoniae	98.8	0.75	0	5384455	31	509627	173548	1029726	57
GCF_001006265.1_ASM100626v1_genomic	GCF_001006265.1	Klebsiella pneumoniae	97.01	0.33	75	5081069	148	127267	32691	383638	58
GCF_002211665.1_ASM221166v1_genomic	GCF_002211665.1	Klebsiella pneumoniae	100	0.81	60	5745128	5	5208104	1149005	5208104	57
GCF_003815075.1_ASM381507v1_genomic	GCF_003815075.1	Klebsiella pneumoniae	99.84	0.14	66.67	5276587	113	85425	46595	257932	58
GCF_003963495.1_ASM396349v1_genomic	GCF_003963495.1	Klebsiella pneumoniae	100	0.11	0	5385944	1	5385844	5385844	5385844	58
GCF_009684615.1_ASM968461v1_genomic	GCF_009684615.1	Klebsiella pneumoniae	99.14	0.12	0	5812894	5	5447404	1162558	5447404	57
GCF_013407495.1_ASM1340749v1_genomic	GCF_013407495.1	Klebsiella pneumoniae	99.54	0.04	0	5005233	3	2143158	1668381	2436811	58
GCF_013706045.1_ASM1370604v1_genomic	GCF_013706045.1	Klebsiella pneumoniae	100	2.05	18.75	5870747	21	5309503	279559	5309503	57
GCF_016859025.1_ASM1685902v1_genomic	GCF_016859025.1	Klebsiella pneumoniae	99.97	0.95	20	5725436	59	480929	97041	1139383	57
GCF_018583165.1_ASM1858316v1_genomic	GCF_018583165.1	Klebsiella pneumoniae	100	0.48	12.5	5854337	5	5315268	1170867	5315268	57
GCF_019284495.1_ASM1928449v1_genomic	GCF_019284495.1	Klebsiella pneumoniae	99.24	0.04	0	5540574	244	99599	20650	271565	58
GCF_019711255.1_ASM1971125v1_genomic	GCF_019711255.1	Klebsiella pneumoniae	99.43	0.33	0	5540574	247	188301	20702	468430	58
GCF_900069965.1_KPN_RH201207_genomic	GCF_900069965.1	Klebsiella pneumoniae	100	0.02	0	5870288	15	3455463	391285	3455463	57
GCF_000069965.1_ASM6996v1_genomic	GCF_000069965.1	Proteus mirabilis	100	0	0	4099895	2	4063606	2049947	4063606	39
GCF_000444425.1_ASM44442v1_genomic	GCF_000444425.1	Proteus mirabilis	100	0	0	3846754	1	3846754	3846754	3846754	39
GCF_000783575.2_ASM78357v2_genomic	GCF_000783575.2	Proteus mirabilis	100	0	0	4077315	1	4077315	4077315	4077315	39
GCF_001281545.1_ASM128154v1_genomic	GCF_001281545.1	Proteus mirabilis	99.46	0	0	3793000	1	3793000	3793000	3793000	39
GCF_001640985.1_ASM164098v1_genomic	GCF_001640985.1	Proteus mirabilis	100	0	0	4272433	1	4272433	4272433	4272433	39
GCF_002055685.1_ASM205568v1_genomic	GCF_002055685.1	Proteus mirabilis	100	0.54	0	4191021	1	4191021	4191021	4191021	39
GCF_002310875.1_ASM231087v1_genomic	GCF_002310875.1	Proteus mirabilis	99.46	0	0	4286593	3	4090879	1428864	4090879	40
GCF_002945235.1_ASM294523v1_genomic	GCF_002945235.1	Proteus mirabilis	100	0	0	4012640	1	4012640	4012640	4012640	39
GCF_003030945.1_ASM303094v1_genomic	GCF_003030945.1	Proteus mirabilis	100	0	0	3935626	2	3144533	1967761	3144533	39
GCF_003073935.1_ASM307393v1_genomic	GCF_003073935.1	Proteus mirabilis	100	0	0	4238045	6	4219380	706340	4219380	39
GCF_003194305.1_ASM319430v1_genomic	GCF_003194305.1	Proteus mirabilis	100	0	0	4101638	2	3909768	2050768	3909768	39
GCF_003855615.1_ASM385561v1_genomic	GCF_003855615.1	Proteus mirabilis	100	0	0	4074828	1	4074828	4074828	4074828	39
GCF_008041895.1_ASM804189v1_genomic	GCF_008041895.1	Proteus mirabilis	100	0	0	4090308	1	4090308	4090308	4090308	39
GCF_008195605.1_ASM819560v1_genomic	GCF_008195605.1	Proteus mirabilis	100	0	0	4204856	2	4158695	2102428	4158695	39
GCF_008630655.1_ASM863065v1_genomic	GCF_008630655.1	Proteus mirabilis	100	0	0	3957207	1	3957207	3957207	3957207	39
GCF_009429045.2_ASM942904v2_genomic	GCF_009429045.2	Proteus mirabilis	100	0	0	4120262	3	4018329	1373420	4018329	39
GCF_009684595.1_ASM968459v1_genomic	GCF_009684595.1	Proteus mirabilis	100	0	0	4105071	16	506894	256480	827606	39
GCF_010442675.1_ASM1044267v1_genomic	GCF_010442675.1	Proteus mirabilis	98.38	0	0	3819372	1	3819372	3819372	3819372	39
GCF_011045575.1_ASM1104557v1_genomic	GCF_011045575.1	Proteus mirabilis	100	0.54	0	4131513	1	4131513	4131513	4131513	40
GCF_011149675.1_ASM1114967v1_genomic	GCF_011149675.1	Proteus mirabilis	100	0	0	4087423	3	4075803	1362474	4075803	39
GCF_011383025.1_ASM1138302v1_genomic	GCF_011383025.1	Proteus mirabilis	100	0	0	4012915	1	4012915	4012915	4012915	39
GCF_013343255.1_ASM1334325v1_genomic	GCF_013343255.1	Proteus mirabilis	100	0	0	4279833	3	4218783	1426611	4218783	40
GCF_014843115.1_ASM1484311v1_genomic	GCF_014843115.1	Proteus mirabilis	100	1.08	0	4072318	1	4072318	4072318	4072318	39

GCF_014931585.1_ASM1493158v1_genomic	GCF_014931585.1	Proteus mirabilis	100	0	0	4038038	1	4038038	4038038	4038038	39
GCF_015693865.1_ASM1569386v1_genomic	GCF_015693865.1	Proteus mirabilis	100	0	0	3919144	1	3919144	3919144	3919144	39
GCF_016772335.1_ASM1677233v1_genomic	GCF_016772335.1	Proteus mirabilis	100	0	0	4216749	1	4216749	4216749	4216749	39
GCF_016939715.1_ASM1693971v1_genomic	GCF_016939715.1	Proteus mirabilis	100	0	0	4296295	3	4166432	1432098	4166432	40
GCF_018138945.1_ASM1813894v1_genomic	GCF_018138945.1	Proteus mirabilis	100	0	0	3924008	1	3924008	3924008	3924008	39
GCF_018336495.1_ASM1833649v1_genomic	GCF_018336495.1	Proteus mirabilis	100	0	0	4180427	2	4014504	2090213	4014504	39
GCF_018972025.1_ASM1897202v1_genomic	GCF_018972025.1	Proteus mirabilis	100	1.08	0	4113626	1	4113626	4113626	4113626	39
GCF_019192645.1_ASM1919264v1_genomic	GCF_019192645.1	Proteus mirabilis	100	0	0	4189249	3	3946111	1396416	3946111	39
GCF_019443785.1_ASM1944378v1_genomic	GCF_019443785.1	Proteus mirabilis	100	0	0	4204682	1	4204682	4204682	4204682	39
GCF_000014625.1_ASM1462v1_genomic	GCF_000014625.1	Pseudomonas aeruginosa	99.68	0.11	0	6537648	1	6537637	6537637	6537637	66
GCF_000017205.1_ASM1720v1_genomic	GCF_000017205.1	Pseudomonas aeruginosa	100	0.17	0	6588339	1	6588339	6588339	6588339	66
GCF_000026645.1_ASM2664v1_genomic	GCF_000026645.1	Pseudomonas aeruginosa	99.68	0.45	0	6601757	1	6601757	6601757	6601757	66
GCF_000226155.1_ASM22615v1_genomic	GCF_000226155.1	Pseudomonas aeruginosa	99.51	0.11	0	6327754	1	6327754	6327754	6327754	67
GCF_000271985.2_ASM27198v2_genomic	GCF_000271985.2	Pseudomonas aeruginosa	99.68	0.22	0	6243825	1	6243825	6243825	6243825	67
GCF_000284555.1_ASM28455v1_genomic	GCF_000284555.1	Pseudomonas aeruginosa	99.68	0.43	0	6764661	1	6764661	6764661	6764661	66
GCF_000414035.1_ASM41403v1_genomic	GCF_000414035.1	Pseudomonas aeruginosa	99.68	0.11	0	6342034	1	6342034	6342034	6342034	67
GCF_000496605.2_ASM49660v2_genomic	GCF_000496605.2	Pseudomonas aeruginosa	99.68	0.43	50	6498072	1	6498072	6498072	6498072	66
GCF_000524595.1_ASM52459v1_genomic	GCF_000524595.1	Pseudomonas aeruginosa	99.68	0.43	0	6433441	1	6433441	6433441	6433441	66
GCF_000568855.2_ASM56885v2_genomic	GCF_000568855.2	Pseudomonas aeruginosa	99.24	0.11	0	6283316	1	6278626	6278626	6278626	66
GCF_000981825.1_ASM98182v1_genomic	GCF_000981825.1	Pseudomonas aeruginosa	99.68	0.11	0	7497593	1	7497593	7497593	7497593	66
GCF_001045685.1_ASM104568v1_genomic	GCF_001045685.1	Pseudomonas aeruginosa	99.68	0.11	0	6317050	1	6317050	6317050	6317050	67
GCF_001293085.1_ASM129308v1_genomic	GCF_001293085.1	Pseudomonas aeruginosa	99.68	0.3	87.5	6500439	1	6500439	6500439	6500439	66
GCF_001606045.1_ASM160604v1_genomic	GCF_001606045.1	Pseudomonas aeruginosa	99.68	0.11	0	6370730	1	6370730	6370730	6370730	66
GCF_001722005.2_ASM172200v2_genomic	GCF_001722005.2	Pseudomonas aeruginosa	99.68	1.73	0	6959251	2	6641902	3479625	6641902	66
GCF_001750705.1_ASM175070v1_genomic	GCF_001750705.1	Pseudomonas aeruginosa	99.03	0.15	0	6866790	1	6866790	6866790	6866790	66
GCF_001874465.1_ASM187446v1_genomic	GCF_001874465.1	Pseudomonas aeruginosa	99.68	0.54	0	6723378	1	6723378	6723378	6723378	66
GCF_001879525.1_ASM187952v1_genomic	GCF_001879525.1	Pseudomonas aeruginosa	99.68	0.11	0	6504659	1	6504659	6504659	6504659	66
GCF_001900195.1_ASM190019v1_genomic	GCF_001900195.1	Pseudomonas aeruginosa	99.59	0.11	0	6213026	1	6213026	6213026	6213026	67
GCF_002223805.1_ASM222380v1_genomic	GCF_002223805.1	Pseudomonas aeruginosa	99.68	0.17	0	6661962	1	6661962	6661962	6661962	66
GCF_002287725.2_ASM228772v2_genomic	GCF_002287725.2	Pseudomonas aeruginosa	99.68	0.47	0	6930893	1	6930893	6930893	6930893	66
GCF_002968515.1_ASM296851v1_genomic	GCF_002968515.1	Pseudomonas aeruginosa	99.68	0.11	0	6463575	1	6463575	6463575	6463575	66
GCF_003025345.2_ASM302534v2_genomic	GCF_003025345.2	Pseudomonas aeruginosa	99.84	0.17	0	6164858	2	6118054	3082429	6118054	67
GCF_003204335.1_ASM320433v1_genomic	GCF_003204335.1	Pseudomonas aeruginosa	99.68	0.49	0	6799785	1	6799785	6799785	6799785	66
GCF_003319235.1_ASM331923v1_genomic	GCF_003319235.1	Pseudomonas aeruginosa	99.68	0.15	0	6876988	1	6876988	6876988	6876988	66
GCF_003571505.1_ASM357150v1_genomic	GCF_003571505.1	Pseudomonas aeruginosa	99.51	0.11	0	6972899	1	6972899	6972899	6972899	66
GCF_004014755.1_ASM401475v2_genomic	GCF_004014755.1	Pseudomonas aeruginosa	99.68	0.11	0	6241875	1	6241875	6241875	6241875	67
GCF_004102665.1_ASM410266v1_genomic	GCF_004102665.1	Pseudomonas aeruginosa	99.68	0.11	0	6383803	1	6383803	6383803	6383803	66
GCF_006971785.1_ASM697178v1_genomic	GCF_006971785.1	Pseudomonas aeruginosa	99.68	0.36	0	6712466	2	6527298	3356233	6527298	66
GCF_008033725.1_ASM803372v1_genomic	GCF_008033725.1	Pseudomonas aeruginosa	99.68	1.08	85.71	6501414	1	6501414	6501414	6501414	66
GCF_009648875.1_ASM964887v1_genomic	GCF_009648875.1	Pseudomonas aeruginosa	99.68	0.11	0	6441924	1	6441924	6441924	6441924	66
GCF_009911735.1_ASM991173v1_genomic	GCF_009911735.1	Pseudomonas aeruginosa	99.68	0.14	0	6397159	1	6397159	6397159	6397159	66
GCF_012935295.1_ASM1293529v1_genomic	GCF_012935295.1	Pseudomonas aeruginosa	99.68	0.11	0	6288195	1	6288195	6288195	6288195	67
GCF_013305765.1_ASM1330576v1_genomic	GCF_013305765.1	Pseudomonas aeruginosa	99.68	0.11	0	6275136	1	6275136	6275136	6275136	67
GCF_014792125.1_ASM1479212v1_genomic	GCF_014792125.1	Pseudomonas aeruginosa	97.01	0.11	0	6923280	2	6893878	3461640	6893878	66
GCF_014854655.1_ASM1485465v1_genomic	GCF_014854655.1	Pseudomonas aeruginosa	99.68	0.22	0	6520277	1	6520277	6520277	6520277	66
GCF_016105505.1_ASM1610550v1_genomic	GCF_016105505.1	Pseudomonas aeruginosa	99.68	1.46	15.79	7157191	1	7157191	7157191	7157191	66
GCF_016126955.1_ASM1612695v1_genomic	GCF_016126955.1	Pseudomonas aeruginosa	99.68	0.11	0	6842475	1	6842475	6842475	6842475	66
GCF_016743035.1_ASM1674303v1_genomic	GCF_016743035.1	Pseudomonas aeruginosa	100	0.4	0	6374121	1	6374121	6374121	6374121	67
GCF_017900915.1_ASM1790091v1_genomic	GCF_017900915.1	Pseudomonas aeruginosa	99.68	0.22	0	6602988	1	6602988	6602988	6602988	66
GCF_018409365.1_ASM1840936v1_genomic	GCF_018409365.1	Pseudomonas aeruginosa	99.68	0.11	0	6411763	1	6411763	6411763	6411763	66
GCF_019466145.1_ASM1946614v1_genomic	GCF_019466145.1	Pseudomonas aeruginosa	99.68	0.11	0	6382345	1	6382345	6382345	6382345	67
GCF_900070375.1_PAO1OR_genomic	GCF_900070375.1	Pseudomonas aeruginosa	99.68	0.11	0	6276469	1	6276469	6276469	6276469	67
GCF_900095805.1_PA14Or_genomic	GCF_900095805.1	Pseudomonas aeruginosa	99.68	0.11	0	6541482	1	6541482	6541482	6541482	66
GCF_900149285.1_PcyII-10_genomic	GCF_900149285.1	Pseudomonas aeruginosa	99.68	0.11	0	6288645	1	6288645	6288645	6288645	67
GCF_900243355.1_RW109_genomic	GCF_900243355.1	Pseudomonas aeruginosa	99.68	2.75	15	7756224	3	7049347	2585408	7049347	65
GCF_900497025.1_ASM90049702v1_genomic	GCF_900497025.1	Pseudomonas aeruginosa	99.68	0.11	0	7231910	2	7207057	3615955	7207057	66
GCF_900636735.1_43941_C01_genomic	GCF_900636735.1	Pseudomonas aeruginosa	99.68	0.21	0	6766292	1	6766292	6766292	6766292	66
GCF_904866275.1_MINF_7A_genomic	GCF_904866275.1	Pseudomonas aeruginosa	99.68	0.19	0	6270455	1	6270455	6270455	6270455	67
