# Supplementary material for: Tracking gut microbiome and bloodstream infection in critically ill adults
Source: PLoS One. 2023 Oct 10;18(10):e0289923. doi: 10.1371/journal.pone.0289923 (PMC10564172; doi:10.1371/journal.pone.0289923)
Supplement: S5 Table — (PDF) [file pone.0289923.s006.pdf]

Table S5. Pair-wise comparisons. The two sequences are shown in the first two columns.

bin1	bin2	hamming	spM	Species	Comments
GCF_900070375.1_PAO1OR_genomic	GCF_004014755.1	3	2.13119797	P. aeruginosa	Different strains but all lab-derived from reference strain PAO1 (Strain PAO1_Orsay, PA0750, LIUYANG-E, AO1161)
GCF_013305765.1	GCF_004014755.1	3	2.13119797	P. aeruginosa	
GCF_900070375.1_PAO1OR_genomic	GCF_004102665.1	10	7.10399323	P. aeruginosa	
GCF_013305765.1	GCF_004102665.1	10	7.10399323	P. aeruginosa	
GCF_004014755.1	GCF_004102665.1	13	9.23519119	P. aeruginosa	
GCF_000014625.1	GCF_900095805.1_PA14Or_genomic	11	7.81439255	P. aeruginosa	Cluster of 3 with no reported connection but all match reference strain PA14 (strain UCBPP-PA14, strain L10, reference strain PA14)
GCF_002223805.1	GCF_900095805.1_PA14Or_genomic	35	24.8639763	P. aeruginosa	
GCF_002223805.1	GCF_000014625.1	46	32.6783688	P. aeruginosa	
GCF_002968515.1	GCF_003204335.1	57	40.4927614	P. aeruginosa	Isolated by same institution different timeframe and different strains (Strain AR_0360 and Strain AR_0110)
GCF_001750705.1	GCF_003319235.1	11	7.81439255	P. aeruginosa	Isolated by same lab similar timeframe but dfferent strains (Strain FA-HZ1, Strain HS9)
GCF_900497025.1	GCF_000981825.1	19	13.4975871	P. aeruginosa	No obvious connection (strain 5486, Carb01 63)
GCF_001971645.1	GCF_002209555.1	10	14.0842293	A. xylosoxidans/A. insolitus	Same isolate goes by different strain names (Strain DSM23807, Strain LMG 6003)
GCF_000508285.1	GCF_001457475.1_NCTC10807_genomic	44	61.970609	A. xylosoxidans/A. insolitus	Same isolate (Strain KM543) available from different sources identified as NBRC15126, ATCC27061, NCTC10807
GCF_002310875.1	GCF_013343255.1	35	14.8421999	P. mirabilis	Isolated by same lab similar timeframe but dfferent strains (StrainT21, Strain L90-1)
GCF_003999775.1	GCF_003028695.1	45	44.378742	E. coli	Cluster of 3 with no obvious connection: Strains C4435, 55989, SCU182
GCF_003028695.1	GCF_013344585.1	59	58.1854617	E. coli	
GCF_003999775.1	GCF_013344585.1	70	69.0335987	E. coli	
GCF_010103655.1	GCF_006349345.1	71	44.3235016	E. faecalis	Cluster of 3 with no obvious connection: Strains JY32; HA-1; TH4125
GCF_010103655.1	GCF_012594215.1	71	44.3235016	E. faecalis	
GCF_006349345.1	GCF_012594215.1	80	49.9419737	E. faecalis	
GCF_003367575.1	GCF_014216355.1	249	245.562372	E. coli	
GCF_006514375.1	GCF_016864435.1	291	286.982532	E. coli	
GCF_007632055.1	GCF_000391485.2	370	230.981628	E. faecalis	
GCF_003112045.1	GCF_003666405.1	623	614.399028	E. coli	
GCF_002287725.2	GCF_001722005.2	876	622.309807	P. aeruginosa	
GCF_001640985.1	GCF_002310875.1	1031	437.208801	P. mirabilis	
GCF_001640985.1	GCF_013343255.1	1062	450.35475	P. mirabilis	
GCF_014843115.1	GCF_018972025.1	1101	466.893201	P. mirabilis	
GCF_900243355.1_RW109_genomic	GCF_900497025.1	1104	784.280852	P. aeruginosa	
GCF_900243355.1_RW109_genomic	GCF_000981825.1	1105	784.991251	P. aeruginosa	
GCF_000240185.1	GCF_009684615.1	1371	457.323785	K. pneumoniae	
GCF_019466145.1	GCF_002968515.1	1396	991.717454	P. aeruginosa	
GCF_019466145.1	GCF_003204335.1	1397	992.427854	P. aeruginosa	
GCF_002634895.1	GCF_004358945.1	1981	1953.65084	E. coli	
GCF_002211665.1	GCF_000240185.1	2292	764.541295	K. pneumoniae	
GCF_002211665.1	GCF_009684615.1	2358	786.556882	K. pneumoniae	
GCF_018986755.2	GCF_001989555.1	2364	1475.78532	E. faecalis	
GCF_011063385.1	GCF_022325125.1	2486	3501.33941	A. xylosoxidans/A. insolitus	
GCF_900069965.1_KPN_RH201207_genomic	GCF_000240185.1	2570	857.273616	K. pneumoniae	
GCF_900069965.1_KPN_RH201207_genomic	GCF_002211665.1	2604	868.614979	K. pneumoniae	
GCF_008245125.1	GCF_011063385.1	2619	3688.65966	A. xylosoxidans/A. insolitus	
GCF_900069965.1_KPN_RH201207_genomic	GCF_009684615.1	2636	879.289203	K. pneumoniae	
GCF_008245125.1	GCF_022325125.1	2650	3732.32077	A. xylosoxidans/A. insolitus	
GCF_001641515.1	GCF_001639685.1	2680	3774.57346	A. xylosoxidans/A. insolitus	
GCF_001639685.1	GCF_022325125.1	2687	3784.43242	A. xylosoxidans/A. insolitus	
GCF_001639685.1	GCF_008245125.1	2736	3853.44514	A. xylosoxidans/A. insolitus	
GCF_009911735.1	GCF_018409365.1	2757	1958.57093	P. aeruginosa	
GCF_003293535.1	GCF_022325125.1	2767	3897.10625	A. xylosoxidans/A. insolitus	
GCF_000783435.2	GCF_001639685.1	2774	3906.96521	A. xylosoxidans/A. insolitus	
GCF_001639685.1	GCF_003293535.1	2778	3912.59891	A. xylosoxidans/A. insolitus	
GCF_001639685.1	GCF_902860205.1_LMG_6001_genomic	2782	3918.2326	A. xylosoxidans/A. insolitus	
GCF_902860205.1_LMG_6001_genomic	GCF_008245125.1	2797	3939.35894	A. xylosoxidans/A. insolitus	
GCF_001639685.1	GCF_011063385.1	2803	3947.80948	A. xylosoxidans/A. insolitus	
GCF_003293535.1	GCF_008245125.1	2806	3952.03475	A. xylosoxidans/A. insolitus	
GCF_902860205.1_LMG_6001_genomic	GCF_022325125.1	2814	3963.30213	A. xylosoxidans/A. insolitus	
GCF_001641515.1	GCF_011063385.1	2817	3967.5274	A. xylosoxidans/A. insolitus	
GCF_902860205.1_LMG_6001_genomic	GCF_011063385.1	2828	3983.02005	A. xylosoxidans/A. insolitus	
GCF_003293535.1	GCF_011063385.1	2834	3991.47059	A. xylosoxidans/A. insolitus	
GCF_001641515.1	GCF_003293535.1	2841	4001.32955	A. xylosoxidans/A. insolitus	
GCF_000783435.2	GCF_011063385.1	2842	4002.73797	A. xylosoxidans/A. insolitus	
GCF_003293535.1	GCF_902860205.1_LMG_6001_genomic	2846	4008.37167	A. xylosoxidans/A. insolitus	
GCF_000783435.2	GCF_022325125.1	2851	4015.41378	A. xylosoxidans/A. insolitus	
GCF_001641515.1	GCF_000783435.2	2857	4023.86432	A. xylosoxidans/A. insolitus	
GCF_001641515.1	GCF_902860205.1_LMG_6001_genomic	2857	4023.86432	A. xylosoxidans/A. insolitus	
GCF_000783435.2	GCF_003293535.1	2860	4028.08959	A. xylosoxidans/A. insolitus	
GCF_001641515.1	GCF_022325125.1	2876	4050.62435	A. xylosoxidans/A. insolitus	
GCF_000783435.2	GCF_902860205.1_LMG_6001_genomic	2879	4054.84962	A. xylosoxidans/A. insolitus	
GCF_001641515.1	GCF_008245125.1	2883	4060.48331	A. xylosoxidans/A. insolitus	
GCF_000783435.2	GCF_008245125.1	2885	4063.30016	A. xylosoxidans/A. insolitus	
GCF_900149285.1_PcyII-10_genomic	GCF_904866275.1_MINF_7A_genomic	2905	2063.71003	P. aeruginosa	
GCF_000166535.2	GCF_004358945.1	3048	3005.92012	E. coli	
GCF_002209555.1	GCF_001639685.1	3381	4761.87793	A. xylosoxidans/A. insolitus	
GCF_001971645.1	GCF_001639685.1	3389	4773.14532	A. xylosoxidans/A. insolitus	
GCF_002209555.1	GCF_003293535.1	3395	4781.59586	A. xylosoxidans/A. insolitus	
GCF_001971645.1	GCF_003293535.1	3403	4792.86324	A. xylosoxidans/A. insolitus	
GCF_002209555.1	GCF_011063385.1	3487	4911.17077	A. xylosoxidans/A. insolitus	
GCF_001971645.1	GCF_011063385.1	3495	4922.43815	A. xylosoxidans/A. insolitus	
GCF_001457475.1_NCTC10807_genomic	GCF_008432465.1	3572	5030.88671	A. xylosoxidans/A. insolitus	
GCF_002209555.1	GCF_008245125.1	3583	5046.37937	A. xylosoxidans/A. insolitus	
GCF_002209555.1	GCF_000783435.2	3584	5047.78779	A. xylosoxidans/A. insolitus	
GCF_002209555.1	GCF_902860205.1_LMG_6001_genomic	3585	5049.19621	A. xylosoxidans/A. insolitus	
GCF_002209555.1	GCF_022325125.1	3586	5050.60464	A. xylosoxidans/A. insolitus	
GCF_001971645.1	GCF_008245125.1	3591	5057.64675	A. xylosoxidans/A. insolitus	
GCF_001971645.1	GCF_000783435.2	3592	5059.05517	A. xylosoxidans/A. insolitus	

GCF_002209555.1	GCF_001641515.1	3593	5060.4636	A. xylosoxidans/A. insolitus
GCF_001971645.1	GCF_902860205.1_LMG_6001_genomic	3593	5060.4636	A. xylosoxidans/A. insolitus
GCF_001971645.1	GCF_022325125.1	3594	5061.87202	A. xylosoxidans/A. insolitus
GCF_001971645.1	GCF_001641515.1	3601	5071.73098	A. xylosoxidans/A. insolitus
GCF_002736085.1	GCF_003666405.1	3602	3552.27175	E. coli
GCF_000508285.1	GCF_008432465.1	3616	5092.85732	A. xylosoxidans/A. insolitus
GCF_000226155.1	GCF_900149285.1_PcyII-10_genomic	3756	2668.25986	P. aeruginosa
GCF_000026645.1	GCF_900149285.1_PcyII-10_genomic	3770	2678.20545	P. aeruginosa
GCF_002736085.1	GCF_003112045.1	3800	3747.53821	E. coli
GCF_000226155.1	GCF_000026645.1	3807	2704.49022	P. aeruginosa
GCF_014792125.1	GCF_014854655.1	3936	2796.13173	P. aeruginosa
GCF_003666405.1	GCF_016801475.1	3941	3886.5916	E. coli
GCF_001879525.1	GCF_000226155.1	3944	2801.81493	P. aeruginosa
GCF_016126955.1	GCF_000226155.1	3948	2804.65653	P. aeruginosa
GCF_001606045.1	GCF_900149285.1_PcyII-10_genomic	3965	2816.73331	P. aeruginosa
GCF_016743035.1	GCF_006971785.1	3985	2830.9413	P. aeruginosa
GCF_016126955.1	GCF_900149285.1_PcyII-10_genomic	3985	2830.9413	P. aeruginosa
GCF_003025345.2	GCF_006971785.1	3999	2840.88689	P. aeruginosa
GCF_008033725.1	GCF_000226155.1	4022	2857.22608	P. aeruginosa
GCF_003319235.1	GCF_900149285.1_PcyII-10_genomic	4027	2860.77807	P. aeruginosa
GCF_001750705.1	GCF_900149285.1_PcyII-10_genomic	4030	2862.90927	P. aeruginosa
GCF_008033725.1	GCF_900149285.1_PcyII-10_genomic	4040	2870.01326	P. aeruginosa
GCF_016126955.1	GCF_000026645.1	4045	2873.56526	P. aeruginosa
GCF_003319235.1	GCF_000226155.1	4046	2874.27566	P. aeruginosa
GCF_000226155.1	GCF_904866275.1_MINF_7A_genomic	4048	2875.69646	P. aeruginosa
GCF_001750705.1	GCF_000226155.1	4049	2876.40686	P. aeruginosa
GCF_000226155.1	GCF_001045685.1	4050	2877.11726	P. aeruginosa
GCF_001606045.1	GCF_000226155.1	4052	2878.53806	P. aeruginosa
GCF_003571505.1	GCF_000226155.1	4057	2882.09005	P. aeruginosa
GCF_009648875.1	GCF_900149285.1_PcyII-10_genomic	4063	2886.35245	P. aeruginosa
GCF_001606045.1	GCF_000026645.1	4064	2887.06285	P. aeruginosa
GCF_000226155.1	GCF_000271985.2	4078	2897.00844	P. aeruginosa
GCF_900070375.1_PAO1OR_genomic	GCF_900149285.1_PcyII-10_genomic	4086	2902.69163	P. aeruginosa
GCF_013305765.1	GCF_900149285.1_PcyII-10_genomic	4086	2902.69163	P. aeruginosa
GCF_004014755.1	GCF_900149285.1_PcyII-10_genomic	4089	2904.82283	P. aeruginosa
GCF_001900195.1	GCF_900149285.1_PcyII-10_genomic	4091	2906.24363	P. aeruginosa
GCF_004102665.1	GCF_900149285.1_PcyII-10_genomic	4096	2909.79563	P. aeruginosa
GCF_008033725.1	GCF_000026645.1	4102	2914.05802	P. aeruginosa
GCF_001879525.1	GCF_900149285.1_PcyII-10_genomic	4104	2915.47882	P. aeruginosa
GCF_018409365.1	GCF_900149285.1_PcyII-10_genomic	4105	2916.18922	P. aeruginosa
GCF_016743035.1	GCF_003025345.2	4123	2928.97641	P. aeruginosa
GCF_001900195.1	GCF_000226155.1	4126	2931.1076	P. aeruginosa
GCF_000226155.1	GCF_000414035.1	4131	2934.6596	P. aeruginosa
GCF_016126955.1	GCF_001900195.1	4132	2935.37	P. aeruginosa
GCF_001879525.1	GCF_000026645.1	4134	2936.7908	P. aeruginosa
GCF_000414035.1	GCF_900149285.1_PcyII-10_genomic	4134	2936.7908	P. aeruginosa
GCF_900243355.1_RW109_genomic	GCF_003571505.1	4138	2939.6324	P. aeruginosa
GCF_900070375.1_PAO1OR_genomic	GCF_000226155.1	4141	2941.76359	P. aeruginosa
GCF_013305765.1	GCF_000226155.1	4141	2941.76359	P. aeruginosa
GCF_018409365.1	GCF_000226155.1	4141	2941.76359	P. aeruginosa
GCF_004014755.1	GCF_000226155.1	4144	2943.89479	P. aeruginosa
GCF_004102665.1	GCF_000226155.1	4151	2948.86759	P. aeruginosa
GCF_000226155.1	GCF_009648875.1	4151	2948.86759	P. aeruginosa
GCF_000166535.2	GCF_002634895.1	4155	4097.63718	E. coli
GCF_003112045.1	GCF_016801475.1	4156	4098.62337	E. coli
GCF_000524595.1	GCF_900149285.1_PcyII-10_genomic	4156	2952.41958	P. aeruginosa
GCF_003571505.1	GCF_014854655.1	4162	2956.68198	P. aeruginosa
GCF_014854655.1	GCF_900149285.1_PcyII-10_genomic	4169	2961.65478	P. aeruginosa
GCF_003666405.1	GCF_003072445.1	4172	4114.40248	E. coli
GCF_016126955.1	GCF_001606045.1	4175	2965.91717	P. aeruginosa
GCF_000226155.1	GCF_000524595.1	4186	2973.73156	P. aeruginosa
GCF_018409365.1	GCF_000026645.1	4186	2973.73156	P. aeruginosa
GCF_003319235.1	GCF_000026645.1	4190	2976.57316	P. aeruginosa
GCF_003666405.1	GCF_003017885.1	4191	4133.14017	E. coli
GCF_001750705.1	GCF_000026645.1	4193	2978.70436	P. aeruginosa
GCF_001045685.1	GCF_000026645.1	4202	2985.09795	P. aeruginosa
GCF_000524595.1	GCF_000026645.1	4204	2986.51875	P. aeruginosa
GCF_001045685.1	GCF_900149285.1_PcyII-10_genomic	4213	2992.91235	P. aeruginosa
GCF_009648875.1	GCF_000026645.1	4214	2993.62275	P. aeruginosa
GCF_014854655.1	GCF_000226155.1	4219	2997.17474	P. aeruginosa
GCF_012935295.1	GCF_900149285.1_PcyII-10_genomic	4230	3004.98913	P. aeruginosa
GCF_000226155.1	GCF_012935295.1	4237	3009.96193	P. aeruginosa
GCF_008033725.1	GCF_016126955.1	4238	3010.67233	P. aeruginosa
GCF_016126955.1	GCF_000524595.1	4239	3011.38273	P. aeruginosa
GCF_003319235.1	GCF_001879525.1	4245	3015.64512	P. aeruginosa
GCF_003571505.1	GCF_900149285.1_PcyII-10_genomic	4245	3015.64512	P. aeruginosa
GCF_012935295.1	GCF_000026645.1	4246	3016.35552	P. aeruginosa
GCF_001750705.1	GCF_001879525.1	4248	3017.77632	P. aeruginosa
GCF_000226155.1	GCF_000568855.2	4251	3019.90752	P. aeruginosa
GCF_018409365.1	GCF_904866275.1_MINF_7A_genomic	4251	3019.90752	P. aeruginosa
GCF_000568855.2	GCF_900149285.1_PcyII-10_genomic	4258	3024.88032	P. aeruginosa
GCF_001900195.1	GCF_000026645.1	4259	3025.59071	P. aeruginosa
GCF_008033725.1	GCF_001879525.1	4262	3027.72191	P. aeruginosa
GCF_900070375.1_PAO1OR_genomic	GCF_003319235.1	4265	3029.85311	P. aeruginosa
GCF_013305765.1	GCF_003319235.1	4265	3029.85311	P. aeruginosa
GCF_004014755.1	GCF_003319235.1	4268	3031.98431	P. aeruginosa
GCF_900070375.1_PAO1OR_genomic	GCF_001750705.1	4270	3033.40511	P. aeruginosa
GCF_013305765.1	GCF_001750705.1	4270	3033.40511	P. aeruginosa
GCF_016126955.1	GCF_009648875.1	4270	3033.40511	P. aeruginosa
GCF_000026645.1	GCF_904866275.1_MINF_7A_genomic	4271	3034.11551	P. aeruginosa
GCF_004014755.1	GCF_001750705.1	4273	3035.53631	P. aeruginosa
GCF_004102665.1	GCF_003319235.1	4275	3036.9571	P. aeruginosa
GCF_001900195.1	GCF_000271985.2	4277	3038.3779	P. aeruginosa
GCF_004102665.1	GCF_001750705.1	4280	3040.5091	P. aeruginosa

GCF_003571505.1	GCF_000026645.1	4284	3043.3507	P. aeruginosa
GCF_003319235.1	GCF_016126955.1	4296	3051.87549	P. aeruginosa
GCF_016126955.1	GCF_018409365.1	4298	3053.29629	P. aeruginosa
GCF_001750705.1	GCF_016126955.1	4299	3054.00669	P. aeruginosa
GCF_000271985.2	GCF_900149285.1_PcyII-10_genomic	4302	3056.13789	P. aeruginosa
GCF_016126955.1	GCF_904866275.1_MINF_7A_genomic	4304	3057.55868	P. aeruginosa
GCF_900070375.1_PAO1OR_genomic	GCF_016126955.1	4305	3058.26908	P. aeruginosa
GCF_013305765.1	GCF_016126955.1	4305	3058.26908	P. aeruginosa
GCF_014854655.1	GCF_000026645.1	4307	3059.68988	P. aeruginosa
GCF_004014755.1	GCF_016126955.1	4308	3060.40028	P. aeruginosa
GCF_009911735.1	GCF_900149285.1_PcyII-10_genomic	4314	3064.66268	P. aeruginosa
GCF_009648875.1	GCF_904866275.1_MINF_7A_genomic	4314	3064.66268	P. aeruginosa
GCF_004102665.1	GCF_016126955.1	4315	3065.37308	P. aeruginosa
GCF_900070375.1_PAO1OR_genomic	GCF_000026645.1	4320	3068.92507	P. aeruginosa
GCF_013305765.1	GCF_000026645.1	4320	3068.92507	P. aeruginosa
GCF_009911735.1	GCF_000026645.1	4321	3069.63547	P. aeruginosa
GCF_004014755.1	GCF_000026645.1	4323	3071.05627	P. aeruginosa
GCF_000414035.1	GCF_000026645.1	4325	3072.47707	P. aeruginosa
GCF_001606045.1	GCF_904866275.1_MINF_7A_genomic	4329	3075.31867	P. aeruginosa
GCF_000524595.1	GCF_904866275.1_MINF_7A_genomic	4329	3075.31867	P. aeruginosa
GCF_004102665.1	GCF_000026645.1	4330	3076.02907	P. aeruginosa
GCF_002968515.1	GCF_900149285.1_PcyII-10_genomic	4331	3076.73947	P. aeruginosa
GCF_016126955.1	GCF_001879525.1	4332	3077.44987	P. aeruginosa
GCF_008033725.1	GCF_001606045.1	4333	3078.16026	P. aeruginosa
GCF_001900195.1	GCF_904866275.1_MINF_7A_genomic	4333	3078.16026	P. aeruginosa
GCF_003204335.1	GCF_900149285.1_PcyII-10_genomic	4334	3078.87066	P. aeruginosa
GCF_003319235.1	GCF_904866275.1_MINF_7A_genomic	4339	3082.42266	P. aeruginosa
GCF_001879525.1	GCF_000524595.1	4340	3083.13306	P. aeruginosa
GCF_003112045.1	GCF_003072445.1	4341	4281.06931	E. coli
GCF_001900195.1	GCF_000414035.1	4341	3083.84346	P. aeruginosa
GCF_014854655.1	GCF_016126955.1	4344	3085.97466	P. aeruginosa
GCF_001900195.1	GCF_018409365.1	4344	3085.97466	P. aeruginosa
GCF_001750705.1	GCF_904866275.1_MINF_7A_genomic	4344	3085.97466	P. aeruginosa
GCF_008033725.1	GCF_009648875.1	4347	3088.10586	P. aeruginosa
GCF_008033725.1	GCF_904866275.1_MINF_7A_genomic	4349	3089.52665	P. aeruginosa
GCF_003571505.1	GCF_016126955.1	4351	3090.94745	P. aeruginosa
GCF_009911735.1	GCF_000226155.1	4353	3092.36825	P. aeruginosa
GCF_001879525.1	GCF_904866275.1_MINF_7A_genomic	4354	3093.07865	P. aeruginosa
GCF_018409365.1	GCF_001606045.1	4355	3093.78905	P. aeruginosa
GCF_900070375.1_PAO1OR_genomic	GCF_001879525.1	4360	3097.34105	P. aeruginosa
GCF_013305765.1	GCF_001879525.1	4360	3097.34105	P. aeruginosa
GCF_008033725.1	GCF_001900195.1	4362	3098.76185	P. aeruginosa
GCF_008033725.1	GCF_018409365.1	4362	3098.76185	P. aeruginosa
GCF_001606045.1	GCF_001045685.1	4362	3098.76185	P. aeruginosa
GCF_001879525.1	GCF_001045685.1	4362	3098.76185	P. aeruginosa
GCF_014854655.1	GCF_001606045.1	4363	3099.47224	P. aeruginosa
GCF_004014755.1	GCF_001879525.1	4363	3099.47224	P. aeruginosa
GCF_001606045.1	GCF_001879525.1	4363	3099.47224	P. aeruginosa
GCF_003204335.1	GCF_000524595.1	4368	3103.02424	P. aeruginosa
GCF_002968515.1	GCF_000524595.1	4369	3103.73464	P. aeruginosa
GCF_004102665.1	GCF_001879525.1	4370	3104.44504	P. aeruginosa
GCF_014792125.1	GCF_009648875.1	4373	3106.57624	P. aeruginosa
GCF_003319235.1	GCF_018409365.1	4382	3112.96983	P. aeruginosa
GCF_001750705.1	GCF_018409365.1	4385	3115.10103	P. aeruginosa
GCF_001900195.1	GCF_001879525.1	4386	3115.81143	P. aeruginosa
GCF_000271985.2	GCF_000026645.1	4386	3115.81143	P. aeruginosa
GCF_900243355.1_RW109_genomic	GCF_900149285.1_PcyII-10_genomic	4386	3115.81143	P. aeruginosa
GCF_018409365.1	GCF_000524595.1	4390	3118.65303	P. aeruginosa
GCF_016126955.1	GCF_001045685.1	4390	3118.65303	P. aeruginosa
GCF_014854655.1	GCF_009648875.1	4393	3120.78422	P. aeruginosa
GCF_008033725.1	GCF_001045685.1	4394	3121.49462	P. aeruginosa
GCF_001606045.1	GCF_000524595.1	4401	3126.46742	P. aeruginosa
GCF_000524595.1	GCF_000568855.2	4401	3126.46742	P. aeruginosa
GCF_003112045.1	GCF_003017885.1	4402	4341.22716	E. coli
GCF_014792125.1	GCF_000226155.1	4402	3127.17782	P. aeruginosa
GCF_000414035.1	GCF_904866275.1_MINF_7A_genomic	4403	3127.88822	P. aeruginosa
GCF_900070375.1_PAO1OR_genomic	GCF_904866275.1_MINF_7A_genomic	4407	3130.72981	P. aeruginosa
GCF_013305765.1	GCF_904866275.1_MINF_7A_genomic	4407	3130.72981	P. aeruginosa
GCF_001606045.1	GCF_000271985.2	4408	3131.44021	P. aeruginosa
GCF_004014755.1	GCF_904866275.1_MINF_7A_genomic	4410	3132.86101	P. aeruginosa
GCF_014792125.1	GCF_016126955.1	4413	3134.99221	P. aeruginosa
GCF_008033725.1	GCF_000568855.2	4413	3134.99221	P. aeruginosa
GCF_003017885.1	GCF_016801475.1	4417	4356.02007	E. coli
GCF_004102665.1	GCF_904866275.1_MINF_7A_genomic	4417	3137.83381	P. aeruginosa
GCF_014792125.1	GCF_900149285.1_PcyII-10_genomic	4418	3138.54421	P. aeruginosa
GCF_016126955.1	GCF_000568855.2	4421	3140.67541	P. aeruginosa
GCF_900243355.1_RW109_genomic	GCF_000226155.1	4424	3142.8066	P. aeruginosa
GCF_000568855.2	GCF_000026645.1	4424	3142.8066	P. aeruginosa
GCF_018409365.1	GCF_001879525.1	4426	3144.2274	P. aeruginosa
GCF_018409365.1	GCF_009648875.1	4426	3144.2274	P. aeruginosa
GCF_001606045.1	GCF_012935295.1	4429	3146.3586	P. aeruginosa
GCF_003319235.1	GCF_008033725.1	4430	3147.069	P. aeruginosa
GCF_003319235.1	GCF_012935295.1	4430	3147.069	P. aeruginosa
GCF_001900195.1	GCF_001045685.1	4430	3147.069	P. aeruginosa
GCF_900243355.1_RW109_genomic	GCF_014854655.1	4431	3147.7794	P. aeruginosa
GCF_001750705.1	GCF_012935295.1	4433	3149.2002	P. aeruginosa
GCF_001900195.1	GCF_001606045.1	4434	3149.9106	P. aeruginosa
GCF_003571505.1	GCF_904866275.1_MINF_7A_genomic	4434	3149.9106	P. aeruginosa
GCF_001750705.1	GCF_008033725.1	4435	3150.621	P. aeruginosa
GCF_008033725.1	GCF_000524595.1	4435	3150.621	P. aeruginosa
GCF_001900195.1	GCF_012935295.1	4436	3151.33139	P. aeruginosa
GCF_014854655.1	GCF_904866275.1_MINF_7A_genomic	4438	3152.75219	P. aeruginosa
GCF_016126955.1	GCF_000414035.1	4439	3153.46259	P. aeruginosa
GCF_003319235.1	GCF_001606045.1	4440	3154.17299	P. aeruginosa

GCF_002968515.1	GCF_001879525.1	4440	3154.17299	P. aeruginosa
GCF_001045685.1	GCF_904866275.1_MINF_7A_genomic	4440	3154.17299	P. aeruginosa
GCF_003319235.1	GCF_009911735.1	4441	3154.88339	P. aeruginosa
GCF_003204335.1	GCF_001879525.1	4441	3154.88339	P. aeruginosa
GCF_014854655.1	GCF_008033725.1	4444	3157.01459	P. aeruginosa
GCF_019466145.1	GCF_900149285.1_PcyII-10_genomic	4444	3157.01459	P. aeruginosa
GCF_001750705.1	GCF_001606045.1	4445	3157.72499	P. aeruginosa
GCF_003319235.1	GCF_000414035.1	4445	3157.72499	P. aeruginosa
GCF_001750705.1	GCF_009911735.1	4446	3158.43539	P. aeruginosa
GCF_003319235.1	GCF_001045685.1	4448	3159.85619	P. aeruginosa
GCF_016126955.1	GCF_009911735.1	4449	3160.56659	P. aeruginosa
GCF_019466145.1	GCF_000524595.1	4449	3160.56659	P. aeruginosa
GCF_001750705.1	GCF_000414035.1	4450	3161.27699	P. aeruginosa
GCF_001750705.1	GCF_001045685.1	4451	3161.98738	P. aeruginosa
GCF_000568855.2	GCF_904866275.1_MINF_7A_genomic	4452	3162.69778	P. aeruginosa
GCF_900070375.1_PAO1OR_genomic	GCF_008033725.1	4453	3163.40818	P. aeruginosa
GCF_013305765.1	GCF_008033725.1	4453	3163.40818	P. aeruginosa
GCF_004014755.1	GCF_008033725.1	4456	3165.53938	P. aeruginosa
GCF_900070375.1_PAO1OR_genomic	GCF_012935295.1	4459	3167.67058	P. aeruginosa
GCF_013305765.1	GCF_012935295.1	4459	3167.67058	P. aeruginosa
GCF_003319235.1	GCF_000524595.1	4460	3168.38098	P. aeruginosa
GCF_003319235.1	GCF_014854655.1	4461	3169.09138	P. aeruginosa
GCF_900070375.1_PAO1OR_genomic	GCF_001606045.1	4461	3169.09138	P. aeruginosa
GCF_013305765.1	GCF_001606045.1	4461	3169.09138	P. aeruginosa
GCF_018409365.1	GCF_000568855.2	4461	3169.09138	P. aeruginosa
GCF_001879525.1	GCF_009648875.1	4462	3169.80178	P. aeruginosa
GCF_004014755.1	GCF_012935295.1	4462	3169.80178	P. aeruginosa
GCF_004102665.1	GCF_008033725.1	4463	3170.51218	P. aeruginosa
GCF_002736085.1	GCF_003017885.1	4464	4402.37121	E. coli
GCF_004014755.1	GCF_001606045.1	4464	3171.22258	P. aeruginosa
GCF_001750705.1	GCF_000524595.1	4465	3171.93298	P. aeruginosa
GCF_016126955.1	GCF_012935295.1	4465	3171.93298	P. aeruginosa
GCF_001750705.1	GCF_014854655.1	4466	3172.64337	P. aeruginosa
GCF_018409365.1	GCF_000414035.1	4466	3172.64337	P. aeruginosa
GCF_004102665.1	GCF_012935295.1	4467	3173.35377	P. aeruginosa
GCF_014854655.1	GCF_018409365.1	4470	3175.48497	P. aeruginosa
GCF_004102665.1	GCF_001606045.1	4471	3176.19537	P. aeruginosa
GCF_014792125.1	GCF_000026645.1	4472	3176.90577	P. aeruginosa
GCF_001900195.1	GCF_009648875.1	4474	3178.32657	P. aeruginosa
GCF_002736085.1	GCF_003072445.1	4476	4414.20554	E. coli
GCF_003072445.1	GCF_016801475.1	4476	4414.20554	E. coli
GCF_003571505.1	GCF_001900195.1	4479	3181.87857	P. aeruginosa
GCF_002968515.1	GCF_018409365.1	4479	3181.87857	P. aeruginosa
GCF_003204335.1	GCF_018409365.1	4480	3182.58897	P. aeruginosa
GCF_009648875.1	GCF_001045685.1	4486	3186.85136	P. aeruginosa
GCF_001606045.1	GCF_000414035.1	4487	3187.56176	P. aeruginosa
GCF_012935295.1	GCF_001045685.1	4489	3188.98256	P. aeruginosa
GCF_001900195.1	GCF_000524595.1	4491	3190.40336	P. aeruginosa
GCF_001879525.1	GCF_000568855.2	4494	3192.53456	P. aeruginosa
GCF_008033725.1	GCF_009911735.1	4495	3193.24495	P. aeruginosa
GCF_002968515.1	GCF_000226155.1	4495	3193.24495	P. aeruginosa
GCF_009911735.1	GCF_001879525.1	4496	3193.95535	P. aeruginosa
GCF_003204335.1	GCF_000226155.1	4496	3193.95535	P. aeruginosa
GCF_003319235.1	GCF_009648875.1	4496	3193.95535	P. aeruginosa
GCF_900070375.1_PAO1OR_genomic	GCF_001900195.1	4499	3196.08655	P. aeruginosa
GCF_013305765.1	GCF_001900195.1	4499	3196.08655	P. aeruginosa
GCF_001750705.1	GCF_009648875.1	4499	3196.08655	P. aeruginosa
GCF_002968515.1	GCF_000026645.1	4499	3196.08655	P. aeruginosa
GCF_000271985.2	GCF_000414035.1	4500	3196.79695	P. aeruginosa
GCF_003571505.1	GCF_001045685.1	4501	3197.50735	P. aeruginosa
GCF_004014755.1	GCF_001900195.1	4502	3198.21775	P. aeruginosa
GCF_003204335.1	GCF_000026645.1	4502	3198.21775	P. aeruginosa
GCF_000271985.2	GCF_001045685.1	4503	3198.92815	P. aeruginosa
GCF_900070375.1_PAO1OR_genomic	GCF_000414035.1	4504	3199.63855	P. aeruginosa
GCF_013305765.1	GCF_000414035.1	4504	3199.63855	P. aeruginosa
GCF_000524595.1	GCF_001045685.1	4504	3199.63855	P. aeruginosa
GCF_009911735.1	GCF_009648875.1	4505	3200.34895	P. aeruginosa
GCF_900636735.1_43941_C01_genomic	GCF_900149285.1_PcyII-10_genomic	4505	3200.34895	P. aeruginosa
GCF_900070375.1_PAO1OR_genomic	GCF_009648875.1	4506	3201.05935	P. aeruginosa
GCF_013305765.1	GCF_009648875.1	4506	3201.05935	P. aeruginosa
GCF_004014755.1	GCF_000414035.1	4507	3201.76975	P. aeruginosa
GCF_003319235.1	GCF_001900195.1	4508	3202.48015	P. aeruginosa
GCF_004102665.1	GCF_001900195.1	4509	3203.19055	P. aeruginosa
GCF_004014755.1	GCF_009648875.1	4509	3203.19055	P. aeruginosa
GCF_001750705.1	GCF_001900195.1	4511	3204.61134	P. aeruginosa
GCF_001606045.1	GCF_009648875.1	4512	3205.32174	P. aeruginosa
GCF_900070375.1_PAO1OR_genomic	GCF_001045685.1	4512	3205.32174	P. aeruginosa
GCF_013305765.1	GCF_001045685.1	4512	3205.32174	P. aeruginosa
GCF_009911735.1	GCF_001606045.1	4514	3206.74254	P. aeruginosa
GCF_004102665.1	GCF_000414035.1	4514	3206.74254	P. aeruginosa
GCF_004014755.1	GCF_001045685.1	4515	3207.45294	P. aeruginosa
GCF_004102665.1	GCF_009648875.1	4516	3208.16334	P. aeruginosa
GCF_900243355.1_RW109_genomic	GCF_000026645.1	4518	3209.58414	P. aeruginosa
GCF_008033725.1	GCF_000414035.1	4521	3211.71534	P. aeruginosa
GCF_012935295.1	GCF_904866275.1_MINF_7A_genomic	4521	3211.71534	P. aeruginosa
GCF_004102665.1	GCF_001045685.1	4522	3212.42574	P. aeruginosa
GCF_014854655.1	GCF_001900195.1	4527	3215.97773	P. aeruginosa
GCF_003319235.1	GCF_000568855.2	4527	3215.97773	P. aeruginosa
GCF_001750705.1	GCF_000568855.2	4530	3218.10893	P. aeruginosa
GCF_008033725.1	GCF_012935295.1	4531	3218.81933	P. aeruginosa
GCF_014854655.1	GCF_001879525.1	4535	3221.66093	P. aeruginosa
GCF_002736085.1	GCF_016801475.1	4536	4473.37719	E. coli
GCF_900070375.1_PAO1OR_genomic	GCF_000524595.1	4536	3222.37133	P. aeruginosa
GCF_013305765.1	GCF_000524595.1	4536	3222.37133	P. aeruginosa

GCF_900070375.1_PAO1OR_genomic	GCF_018409365.1	4538	3223.79213	P. aeruginosa
GCF_013305765.1	GCF_018409365.1	4538	3223.79213	P. aeruginosa
GCF_004014755.1	GCF_000524595.1	4539	3224.50253	P. aeruginosa
GCF_004014755.1	GCF_018409365.1	4541	3225.92332	P. aeruginosa
GCF_001879525.1	GCF_012935295.1	4541	3225.92332	P. aeruginosa
GCF_003571505.1	GCF_000524595.1	4542	3226.63372	P. aeruginosa
GCF_003571505.1	GCF_009648875.1	4543	3227.34412	P. aeruginosa
GCF_900243355.1_RW109_genomic	GCF_016126955.1	4544	3228.05452	P. aeruginosa
GCF_004102665.1	GCF_000524595.1	4546	3229.47532	P. aeruginosa
GCF_900070375.1_PAO1OR_genomic	GCF_014854655.1	4547	3230.18572	P. aeruginosa
GCF_013305765.1	GCF_014854655.1	4547	3230.18572	P. aeruginosa
GCF_003571505.1	GCF_008033725.1	4547	3230.18572	P. aeruginosa
GCF_004102665.1	GCF_018409365.1	4548	3230.89612	P. aeruginosa
GCF_009648875.1	GCF_000414035.1	4548	3230.89612	P. aeruginosa
GCF_019466145.1	GCF_018409365.1	4549	3231.60652	P. aeruginosa
GCF_019466145.1	GCF_001879525.1	4549	3231.60652	P. aeruginosa
GCF_004014755.1	GCF_014854655.1	4550	3232.31692	P. aeruginosa
GCF_016126955.1	GCF_000271985.2	4555	3235.86891	P. aeruginosa
GCF_900243355.1_RW109_genomic	GCF_904866275.1_MINF_7A_genomic	4556	3236.57931	P. aeruginosa
GCF_004102665.1	GCF_014854655.1	4557	3237.28971	P. aeruginosa
GCF_016105505.1	GCF_900149285.1_PcyII-10_genomic	4557	3237.28971	P. aeruginosa
GCF_003319235.1	GCF_003571505.1	4558	3238.00011	P. aeruginosa
GCF_008033725.1	GCF_000271985.2	4558	3238.00011	P. aeruginosa
GCF_001750705.1	GCF_003571505.1	4561	3240.13131	P. aeruginosa
GCF_014854655.1	GCF_000524595.1	4562	3240.84171	P. aeruginosa
GCF_000271985.2	GCF_904866275.1_MINF_7A_genomic	4562	3240.84171	P. aeruginosa
GCF_000414035.1	GCF_012935295.1	4563	3241.55211	P. aeruginosa
GCF_014792125.1	GCF_904866275.1_MINF_7A_genomic	4563	3241.55211	P. aeruginosa
GCF_003571505.1	GCF_001606045.1	4567	3244.39371	P. aeruginosa
GCF_000414035.1	GCF_001045685.1	4567	3244.39371	P. aeruginosa
GCF_018409365.1	GCF_000271985.2	4570	3246.5249	P. aeruginosa
GCF_001879525.1	GCF_000414035.1	4571	3247.2353	P. aeruginosa
GCF_900243355.1_RW109_genomic	GCF_001879525.1	4577	3251.4977	P. aeruginosa
GCF_003571505.1	GCF_001879525.1	4577	3251.4977	P. aeruginosa
GCF_001606045.1	GCF_000568855.2	4577	3251.4977	P. aeruginosa
GCF_014792125.1	GCF_001606045.1	4578	3252.2081	P. aeruginosa
GCF_900243355.1_RW109_genomic	GCF_008033725.1	4582	3255.0497	P. aeruginosa
GCF_009911735.1	GCF_904866275.1_MINF_7A_genomic	4584	3256.47049	P. aeruginosa
GCF_014792125.1	GCF_001879525.1	4592	3262.15369	P. aeruginosa
GCF_009648875.1	GCF_000524595.1	4594	3263.57449	P. aeruginosa
GCF_000524595.1	GCF_012935295.1	4598	3266.41609	P. aeruginosa
GCF_018409365.1	GCF_012935295.1	4599	3267.12648	P. aeruginosa
GCF_900636735.1_43941_C01_genomic	GCF_000226155.1	4604	3270.67848	P. aeruginosa
GCF_003571505.1	GCF_014792125.1	4608	3273.52008	P. aeruginosa
GCF_003319235.1	GCF_000271985.2	4608	3273.52008	P. aeruginosa
GCF_003571505.1	GCF_000414035.1	4609	3274.23048	P. aeruginosa
GCF_016105505.1	GCF_000226155.1	4611	3275.65128	P. aeruginosa
GCF_018409365.1	GCF_001045685.1	4612	3276.36168	P. aeruginosa
GCF_001750705.1	GCF_000271985.2	4613	3277.07207	P. aeruginosa
GCF_003571505.1	GCF_000271985.2	4615	3278.49287	P. aeruginosa
GCF_001900195.1	GCF_000568855.2	4617	3279.91367	P. aeruginosa
GCF_016105505.1	GCF_000524595.1	4618	3280.62407	P. aeruginosa
GCF_900009115.1_BN2877_genomic	GCF_008432465.1	4620	6506.91395	A. xylosoxidans/A. insolitus
GCF_014854655.1	GCF_012935295.1	4620	3282.04487	P. aeruginosa
GCF_003319235.1	GCF_014792125.1	4623	3284.17607	P. aeruginosa
GCF_001900195.1	GCF_009911735.1	4623	3284.17607	P. aeruginosa
GCF_001750705.1	GCF_014792125.1	4626	3286.30727	P. aeruginosa
GCF_003319235.1	GCF_016105505.1	4630	3289.14886	P. aeruginosa
GCF_003571505.1	GCF_012935295.1	4631	3289.85926	P. aeruginosa
GCF_009648875.1	GCF_000271985.2	4634	3291.99046	P. aeruginosa
GCF_001750705.1	GCF_016105505.1	4635	3292.70086	P. aeruginosa
GCF_003204335.1	GCF_016126955.1	4636	3293.41126	P. aeruginosa
GCF_000524595.1	GCF_000271985.2	4636	3293.41126	P. aeruginosa
GCF_002968515.1	GCF_016126955.1	4637	3294.12166	P. aeruginosa
GCF_003571505.1	GCF_018409365.1	4638	3294.83206	P. aeruginosa
GCF_900070375.1_PAO1OR_genomic	GCF_003571505.1	4639	3295.54246	P. aeruginosa
GCF_013305765.1	GCF_003571505.1	4639	3295.54246	P. aeruginosa
GCF_014854655.1	GCF_000568855.2	4641	3296.96326	P. aeruginosa
GCF_004014755.1	GCF_003571505.1	4642	3297.67366	P. aeruginosa
GCF_900243355.1_RW109_genomic	GCF_001606045.1	4642	3297.67366	P. aeruginosa
GCF_900070375.1_PAO1OR_genomic	GCF_000271985.2	4642	3297.67366	P. aeruginosa
GCF_013305765.1	GCF_000271985.2	4642	3297.67366	P. aeruginosa
GCF_009648875.1	GCF_012935295.1	4642	3297.67366	P. aeruginosa
GCF_004014755.1	GCF_000271985.2	4645	3299.80485	P. aeruginosa
GCF_000524595.1	GCF_000414035.1	4645	3299.80485	P. aeruginosa
GCF_900636735.1_43941_C01_genomic	GCF_000026645.1	4645	3299.80485	P. aeruginosa
GCF_000568855.2	GCF_000414035.1	4646	3300.51525	P. aeruginosa
GCF_004102665.1	GCF_003571505.1	4649	3302.64645	P. aeruginosa
GCF_014792125.1	GCF_008033725.1	4650	3303.35685	P. aeruginosa
GCF_000568855.2	GCF_001045685.1	4650	3303.35685	P. aeruginosa
GCF_004102665.1	GCF_000271985.2	4652	3304.77765	P. aeruginosa
GCF_009648875.1	GCF_000568855.2	4654	3306.19845	P. aeruginosa
GCF_900636735.1_43941_C01_genomic	GCF_016126955.1	4657	3308.32965	P. aeruginosa
GCF_002968515.1	GCF_001606045.1	4657	3308.32965	P. aeruginosa
GCF_003204335.1	GCF_001606045.1	4658	3309.04004	P. aeruginosa
GCF_001879525.1	GCF_000271985.2	4660	3310.46084	P. aeruginosa
GCF_009911735.1	GCF_000414035.1	4660	3310.46084	P. aeruginosa
GCF_016105505.1	GCF_012935295.1	4662	3311.88164	P. aeruginosa
GCF_014854655.1	GCF_001045685.1	4668	3316.14404	P. aeruginosa
GCF_900243355.1_RW109_genomic	GCF_001900195.1	4672	3318.98564	P. aeruginosa
GCF_014854655.1	GCF_009911735.1	4681	3325.37923	P. aeruginosa
GCF_002968515.1	GCF_014854655.1	4683	3326.80003	P. aeruginosa
GCF_009911735.1	GCF_000524595.1	4683	3326.80003	P. aeruginosa
GCF_003204335.1	GCF_014854655.1	4684	3327.51043	P. aeruginosa

GCF_014792125.1	GCF_001900195.1	4684	3327.51043	P. aeruginosa
GCF_900243355.1_RW109_genomic	GCF_000414035.1	4684	3327.51043	P. aeruginosa
GCF_009911735.1	GCF_001045685.1	4693	3333.90402	P. aeruginosa
GCF_009911735.1	GCF_000568855.2	4695	3335.32482	P. aeruginosa
GCF_014792125.1	GCF_001045685.1	4695	3335.32482	P. aeruginosa
GCF_900243355.1_RW109_genomic	GCF_018409365.1	4696	3336.03522	P. aeruginosa
GCF_002968515.1	GCF_008033725.1	4697	3336.74562	P. aeruginosa
GCF_900070375.1_PAO1OR_genomic	GCF_009911735.1	4697	3336.74562	P. aeruginosa
GCF_013305765.1	GCF_009911735.1	4697	3336.74562	P. aeruginosa
GCF_003204335.1	GCF_008033725.1	4700	3338.87682	P. aeruginosa
GCF_004014755.1	GCF_009911735.1	4700	3338.87682	P. aeruginosa
GCF_019466145.1	GCF_000226155.1	4701	3339.58722	P. aeruginosa
GCF_016105505.1	GCF_000026645.1	4701	3339.58722	P. aeruginosa
GCF_019466145.1	GCF_000026645.1	4703	3341.00801	P. aeruginosa
GCF_014854655.1	GCF_000414035.1	4704	3341.71841	P. aeruginosa
GCF_000271985.2	GCF_012935295.1	4705	3342.42881	P. aeruginosa
GCF_004102665.1	GCF_009911735.1	4707	3343.84961	P. aeruginosa
GCF_019466145.1	GCF_001606045.1	4713	3348.11201	P. aeruginosa
GCF_003319235.1	GCF_900636735.1_43941_C01_genomic	4717	3350.9536	P. aeruginosa
GCF_900070375.1_PAO1OR_genomic	GCF_000568855.2	4718	3351.664	P. aeruginosa
GCF_013305765.1	GCF_000568855.2	4718	3351.664	P. aeruginosa
GCF_900636735.1_43941_C01_genomic	GCF_001879525.1	4719	3352.3744	P. aeruginosa
GCF_001750705.1	GCF_900636735.1_43941_C01_genomic	4720	3353.0848	P. aeruginosa
GCF_004014755.1	GCF_000568855.2	4721	3353.7952	P. aeruginosa
GCF_004102665.1	GCF_000568855.2	4728	3358.768	P. aeruginosa
GCF_900070375.1_PAO1OR_genomic	GCF_016105505.1	4731	3360.8992	P. aeruginosa
GCF_013305765.1	GCF_016105505.1	4731	3360.8992	P. aeruginosa
GCF_014792125.1	GCF_000524595.1	4733	3362.31999	P. aeruginosa
GCF_004014755.1	GCF_016105505.1	4734	3363.03039	P. aeruginosa
GCF_016105505.1	GCF_018409365.1	4736	3364.45119	P. aeruginosa
GCF_002968515.1	GCF_009648875.1	4736	3364.45119	P. aeruginosa
GCF_003204335.1	GCF_009648875.1	4737	3365.16159	P. aeruginosa
GCF_900243355.1_RW109_genomic	GCF_003319235.1	4738	3365.87199	P. aeruginosa
GCF_002968515.1	GCF_003319235.1	4738	3365.87199	P. aeruginosa
GCF_003204335.1	GCF_003319235.1	4739	3366.58239	P. aeruginosa
GCF_004102665.1	GCF_016105505.1	4741	3368.00319	P. aeruginosa
GCF_002968515.1	GCF_000568855.2	4741	3368.00319	P. aeruginosa
GCF_900243355.1_RW109_genomic	GCF_001750705.1	4743	3369.42399	P. aeruginosa
GCF_002968515.1	GCF_001750705.1	4743	3369.42399	P. aeruginosa
GCF_003204335.1	GCF_001750705.1	4744	3370.13439	P. aeruginosa
GCF_003204335.1	GCF_000568855.2	4744	3370.13439	P. aeruginosa
GCF_900497025.1	GCF_003571505.1	4746	3371.55518	P. aeruginosa
GCF_002634895.1	GCF_019614135.1	4747	4681.46418	E. coli
GCF_000981825.1	GCF_003571505.1	4747	3372.26558	P. aeruginosa
GCF_900243355.1_RW109_genomic	GCF_001045685.1	4748	3372.97598	P. aeruginosa
GCF_014854655.1	GCF_000271985.2	4749	3373.68638	P. aeruginosa
GCF_000568855.2	GCF_000271985.2	4751	3375.10718	P. aeruginosa
GCF_016105505.1	GCF_001606045.1	4755	3377.94878	P. aeruginosa
GCF_900243355.1_RW109_genomic	GCF_009911735.1	4756	3378.65918	P. aeruginosa
GCF_900243355.1_RW109_genomic	GCF_014792125.1	4758	3380.07998	P. aeruginosa
GCF_900070375.1_PAO1OR_genomic	GCF_014792125.1	4759	3380.79038	P. aeruginosa
GCF_013305765.1	GCF_014792125.1	4759	3380.79038	P. aeruginosa
GCF_004014755.1	GCF_014792125.1	4762	3382.92157	P. aeruginosa
GCF_016105505.1	GCF_008033725.1	4763	3383.63197	P. aeruginosa
GCF_900243355.1_RW109_genomic	GCF_009648875.1	4763	3383.63197	P. aeruginosa
GCF_003204335.1	GCF_904866275.1_MINF_7A_genomic	4766	3385.76317	P. aeruginosa
GCF_003571505.1	GCF_000568855.2	4767	3386.47357	P. aeruginosa
GCF_002968515.1	GCF_904866275.1_MINF_7A_genomic	4767	3386.47357	P. aeruginosa
GCF_002968515.1	GCF_001045685.1	4768	3387.18397	P. aeruginosa
GCF_004102665.1	GCF_014792125.1	4769	3387.89437	P. aeruginosa
GCF_003204335.1	GCF_001045685.1	4769	3387.89437	P. aeruginosa
GCF_016105505.1	GCF_016126955.1	4773	3390.73597	P. aeruginosa
GCF_014792125.1	GCF_012935295.1	4777	3393.57756	P. aeruginosa
GCF_900070375.1_PAO1OR_genomic	GCF_002968515.1	4778	3394.28796	P. aeruginosa
GCF_013305765.1	GCF_002968515.1	4778	3394.28796	P. aeruginosa
GCF_001293085.1	GCF_000026645.1	4778	3394.28796	P. aeruginosa
GCF_000496605.2	GCF_000026645.1	4778	3394.28796	P. aeruginosa
GCF_016105505.1	GCF_001879525.1	4779	3394.99836	P. aeruginosa
GCF_900070375.1_PAO1OR_genomic	GCF_003204335.1	4780	3395.70876	P. aeruginosa
GCF_013305765.1	GCF_003204335.1	4780	3395.70876	P. aeruginosa
GCF_004014755.1	GCF_002968515.1	4781	3396.41916	P. aeruginosa
GCF_004014755.1	GCF_003204335.1	4783	3397.83996	P. aeruginosa
GCF_014792125.1	GCF_000414035.1	4785	3399.26076	P. aeruginosa
GCF_004102665.1	GCF_002968515.1	4788	3401.39196	P. aeruginosa
GCF_004102665.1	GCF_003204335.1	4790	3402.81276	P. aeruginosa
GCF_019466145.1	GCF_014854655.1	4792	3404.23355	P. aeruginosa
GCF_000568855.2	GCF_012935295.1	4792	3404.23355	P. aeruginosa
GCF_900636735.1_43941_C01_genomic	GCF_008033725.1	4794	3405.65435	P. aeruginosa
GCF_016105505.1	GCF_000568855.2	4800	3409.91675	P. aeruginosa
GCF_019466145.1	GCF_016126955.1	4801	3410.62715	P. aeruginosa
GCF_900070375.1_PAO1OR_genomic	GCF_900636735.1_43941_C01_genomic	4804	3412.75835	P. aeruginosa
GCF_013305765.1	GCF_900636735.1_43941_C01_genomic	4804	3412.75835	P. aeruginosa
GCF_004014755.1	GCF_900636735.1_43941_C01_genomic	4807	3414.88954	P. aeruginosa
GCF_900070375.1_PAO1OR_genomic	GCF_900243355.1_RW109_genomic	4810	3417.02074	P. aeruginosa
GCF_013305765.1	GCF_900243355.1_RW109_genomic	4810	3417.02074	P. aeruginosa
GCF_004014755.1	GCF_900243355.1_RW109_genomic	4813	3419.15194	P. aeruginosa
GCF_004102665.1	GCF_900636735.1_43941_C01_genomic	4814	3419.86234	P. aeruginosa
GCF_900009115.1_BN2877_genomic	GCF_001457475.1_NCTC10807_genomic	4817	6784.37327	A. xylosoxidans/A. insolitus
GCF_001293085.1	GCF_000226155.1	4817	3421.99354	P. aeruginosa
GCF_000496605.2	GCF_000226155.1	4817	3421.99354	P. aeruginosa
GCF_019466145.1	GCF_003319235.1	4818	3422.70394	P. aeruginosa
GCF_004102665.1	GCF_900243355.1_RW109_genomic	4820	3424.12473	P. aeruginosa
GCF_002968515.1	GCF_000414035.1	4820	3424.12473	P. aeruginosa
GCF_900636735.1_43941_C01_genomic	GCF_904866275.1_MINF_7A_genomic	4820	3424.12473	P. aeruginosa

GCF_014792125.1	GCF_018409365.1	4822	3425.54553	P. aeruginosa
GCF_900636735.1_43941_C01_genomic	GCF_001606045.1	4822	3425.54553	P. aeruginosa
GCF_003204335.1	GCF_000414035.1	4822	3425.54553	P. aeruginosa
GCF_019466145.1	GCF_001750705.1	4823	3426.25593	P. aeruginosa
GCF_900243355.1_RW109_genomic	GCF_000524595.1	4824	3426.96633	P. aeruginosa
GCF_002968515.1	GCF_001900195.1	4826	3428.38713	P. aeruginosa
GCF_016105505.1	GCF_001045685.1	4826	3428.38713	P. aeruginosa
GCF_003204335.1	GCF_001900195.1	4827	3429.09753	P. aeruginosa
GCF_900636735.1_43941_C01_genomic	GCF_000524595.1	4827	3429.09753	P. aeruginosa
GCF_001293085.1	GCF_016126955.1	4828	3429.80793	P. aeruginosa
GCF_000496605.2	GCF_016126955.1	4828	3429.80793	P. aeruginosa
GCF_001293085.1	GCF_900149285.1_PcyII-10_genomic	4831	3431.93913	P. aeruginosa
GCF_000496605.2	GCF_900149285.1_PcyII-10_genomic	4831	3431.93913	P. aeruginosa
GCF_009911735.1	GCF_012935295.1	4836	3435.49112	P. aeruginosa
GCF_900636735.1_43941_C01_genomic	GCF_009648875.1	4840	3438.33272	P. aeruginosa
GCF_009911735.1	GCF_000271985.2	4842	3439.75352	P. aeruginosa
GCF_900243355.1_RW109_genomic	GCF_000568855.2	4843	3440.46392	P. aeruginosa
GCF_900636735.1_43941_C01_genomic	GCF_012935295.1	4845	3441.88472	P. aeruginosa
GCF_014792125.1	GCF_000568855.2	4848	3444.01592	P. aeruginosa
GCF_016105505.1	GCF_904866275.1_MINF_7A_genomic	4853	3447.56791	P. aeruginosa
GCF_019466145.1	GCF_008033725.1	4860	3452.54071	P. aeruginosa
GCF_900009115.1_BN2877_genomic	GCF_000508285.1	4861	6846.34387	A. xylosoxidans/A. insolitus
GCF_001293085.1	GCF_000524595.1	4862	3453.96151	P. aeruginosa
GCF_000496605.2	GCF_000524595.1	4862	3453.96151	P. aeruginosa
GCF_900636735.1_43941_C01_genomic	GCF_000414035.1	4863	3454.67191	P. aeruginosa
GCF_016105505.1	GCF_000414035.1	4871	3460.3551	P. aeruginosa
GCF_016105505.1	GCF_009911735.1	4873	3461.7759	P. aeruginosa
GCF_003571505.1	GCF_009911735.1	4874	3462.4863	P. aeruginosa
GCF_900243355.1_RW109_genomic	GCF_000271985.2	4877	3464.6175	P. aeruginosa
GCF_014854655.1	GCF_900636735.1_43941_C01_genomic	4879	3466.03829	P. aeruginosa
GCF_014854655.1	GCF_016105505.1	4885	3470.30069	P. aeruginosa
GCF_003571505.1	GCF_900636735.1_43941_C01_genomic	4887	3471.72149	P. aeruginosa
GCF_016105505.1	GCF_001900195.1	4889	3473.14229	P. aeruginosa
GCF_003017885.1	GCF_003072445.1	4896	4828.40713	E. coli
GCF_900070375.1_PAO1OR_genomic	GCF_019466145.1	4896	3478.11508	P. aeruginosa
GCF_013305765.1	GCF_019466145.1	4896	3478.11508	P. aeruginosa
GCF_900636735.1_43941_C01_genomic	GCF_001045685.1	4898	3479.53588	P. aeruginosa
GCF_004014755.1	GCF_019466145.1	4899	3480.24628	P. aeruginosa
GCF_019466145.1	GCF_001045685.1	4900	3480.95668	P. aeruginosa
GCF_900636735.1_43941_C01_genomic	GCF_001900195.1	4902	3482.37748	P. aeruginosa
GCF_001293085.1	GCF_001879525.1	4903	3483.08788	P. aeruginosa
GCF_000496605.2	GCF_001879525.1	4903	3483.08788	P. aeruginosa
GCF_004102665.1	GCF_019466145.1	4906	3485.21908	P. aeruginosa
GCF_004358945.1	GCF_019614135.1	4907	4839.25527	E. coli
GCF_002968515.1	GCF_012935295.1	4908	3486.63988	P. aeruginosa
GCF_003204335.1	GCF_012935295.1	4909	3487.35027	P. aeruginosa
GCF_002968515.1	GCF_009911735.1	4914	3490.90227	P. aeruginosa
GCF_003204335.1	GCF_009911735.1	4915	3491.61267	P. aeruginosa
GCF_019466145.1	GCF_009648875.1	4917	3493.03347	P. aeruginosa
GCF_900636735.1_43941_C01_genomic	GCF_000271985.2	4918	3493.74387	P. aeruginosa
GCF_019466145.1	GCF_000414035.1	4921	3495.87507	P. aeruginosa
GCF_900243355.1_RW109_genomic	GCF_012935295.1	4921	3495.87507	P. aeruginosa
GCF_016105505.1	GCF_000271985.2	4925	3498.71666	P. aeruginosa
GCF_016105505.1	GCF_009648875.1	4932	3503.68946	P. aeruginosa
GCF_003204335.1	GCF_003571505.1	4939	3508.66225	P. aeruginosa
GCF_002968515.1	GCF_003571505.1	4940	3509.37265	P. aeruginosa
GCF_019466145.1	GCF_904866275.1_MINF_7A_genomic	4942	3510.79345	P. aeruginosa
GCF_014792125.1	GCF_000271985.2	4943	3511.50385	P. aeruginosa
GCF_014792125.1	GCF_900636735.1_43941_C01_genomic	4946	3513.63505	P. aeruginosa
GCF_002968515.1	GCF_001293085.1	4948	3515.05585	P. aeruginosa
GCF_002968515.1	GCF_000496605.2	4948	3515.05585	P. aeruginosa
GCF_003204335.1	GCF_001293085.1	4951	3517.18705	P. aeruginosa
GCF_003204335.1	GCF_000496605.2	4951	3517.18705	P. aeruginosa
GCF_001293085.1	GCF_003319235.1	4951	3517.18705	P. aeruginosa
GCF_000496605.2	GCF_003319235.1	4951	3517.18705	P. aeruginosa
GCF_001293085.1	GCF_001750705.1	4954	3519.31824	P. aeruginosa
GCF_000496605.2	GCF_001750705.1	4954	3519.31824	P. aeruginosa
GCF_002968515.1	GCF_000271985.2	4957	3521.44944	P. aeruginosa
GCF_900243355.1_RW109_genomic	GCF_900636735.1_43941_C01_genomic	4958	3522.15984	P. aeruginosa
GCF_014792125.1	GCF_009911735.1	4960	3523.58064	P. aeruginosa
GCF_003204335.1	GCF_000271985.2	4960	3523.58064	P. aeruginosa
GCF_900497025.1	GCF_900149285.1_PcyII-10_genomic	4966	3527.84304	P. aeruginosa
GCF_900636735.1_43941_C01_genomic	GCF_016105505.1	4967	3528.55344	P. aeruginosa
GCF_900636735.1_43941_C01_genomic	GCF_018409365.1	4967	3528.55344	P. aeruginosa
GCF_000981825.1	GCF_900149285.1_PcyII-10_genomic	4967	3528.55344	P. aeruginosa
GCF_002968515.1	GCF_014792125.1	4969	3529.97423	P. aeruginosa
GCF_003571505.1	GCF_016105505.1	4969	3529.97423	P. aeruginosa
GCF_003204335.1	GCF_014792125.1	4970	3530.68463	P. aeruginosa
GCF_014792125.1	GCF_016105505.1	4979	3537.07823	P. aeruginosa
GCF_900243355.1_RW109_genomic	GCF_002968515.1	4981	3538.49903	P. aeruginosa
GCF_900243355.1_RW109_genomic	GCF_003204335.1	4984	3540.63022	P. aeruginosa
GCF_002968515.1	GCF_900636735.1_43941_C01_genomic	4984	3540.63022	P. aeruginosa
GCF_003204335.1	GCF_900636735.1_43941_C01_genomic	4986	3542.05102	P. aeruginosa
GCF_001293085.1	GCF_018409365.1	4992	3546.31342	P. aeruginosa
GCF_000496605.2	GCF_018409365.1	4992	3546.31342	P. aeruginosa
GCF_019466145.1	GCF_000568855.2	5001	3552.70701	P. aeruginosa
GCF_001293085.1	GCF_008033725.1	5003	3554.12781	P. aeruginosa
GCF_000496605.2	GCF_008033725.1	5003	3554.12781	P. aeruginosa
GCF_001293085.1	GCF_904866275.1_MINF_7A_genomic	5010	3559.10061	P. aeruginosa
GCF_000496605.2	GCF_904866275.1_MINF_7A_genomic	5010	3559.10061	P. aeruginosa
GCF_019466145.1	GCF_001900195.1	5011	3559.81101	P. aeruginosa
GCF_001293085.1	GCF_009648875.1	5017	3564.0734	P. aeruginosa
GCF_000496605.2	GCF_009648875.1	5017	3564.0734	P. aeruginosa
GCF_900497025.1	GCF_000226155.1	5026	3570.467	P. aeruginosa

GCF_000981825.1	GCF_000226155.1	5027	3571.17739	P. aeruginosa
GCF_001293085.1	GCF_001606045.1	5033	3575.43979	P. aeruginosa
GCF_000496605.2	GCF_001606045.1	5033	3575.43979	P. aeruginosa
GCF_900497025.1	GCF_014854655.1	5038	3578.99179	P. aeruginosa
GCF_000981825.1	GCF_014854655.1	5039	3579.70219	P. aeruginosa
GCF_019466145.1	GCF_012935295.1	5041	3581.12299	P. aeruginosa
GCF_001293085.1	GCF_009911735.1	5046	3584.67498	P. aeruginosa
GCF_000496605.2	GCF_009911735.1	5046	3584.67498	P. aeruginosa
GCF_019466145.1	GCF_009911735.1	5049	3586.80618	P. aeruginosa
GCF_900636735.1_43941_C01_genomic	GCF_000568855.2	5054	3590.35818	P. aeruginosa
GCF_900636735.1_43941_C01_genomic	GCF_009911735.1	5055	3591.06858	P. aeruginosa
GCF_001293085.1	GCF_001900195.1	5064	3597.46217	P. aeruginosa
GCF_000496605.2	GCF_001900195.1	5064	3597.46217	P. aeruginosa
GCF_900243355.1_RW109_genomic	GCF_016105505.1	5071	3602.43496	P. aeruginosa
GCF_019466145.1	GCF_003571505.1	5103	3625.16774	P. aeruginosa
GCF_019466145.1	GCF_014792125.1	5103	3625.16774	P. aeruginosa
GCF_002968515.1	GCF_016105505.1	5103	3625.16774	P. aeruginosa
GCF_003204335.1	GCF_016105505.1	5104	3625.87814	P. aeruginosa
GCF_019466145.1	GCF_900636735.1_43941_C01_genomic	5109	3629.43014	P. aeruginosa
GCF_900243355.1_RW109_genomic	GCF_019466145.1	5111	3630.85094	P. aeruginosa
GCF_900070375.1_PAO1OR_genomic	GCF_001293085.1	5117	3635.11333	P. aeruginosa
GCF_013305765.1	GCF_001293085.1	5117	3635.11333	P. aeruginosa
GCF_900070375.1_PAO1OR_genomic	GCF_000496605.2	5117	3635.11333	P. aeruginosa
GCF_013305765.1	GCF_000496605.2	5117	3635.11333	P. aeruginosa
GCF_004014755.1	GCF_001293085.1	5120	3637.24453	P. aeruginosa
GCF_004014755.1	GCF_000496605.2	5120	3637.24453	P. aeruginosa
GCF_004102665.1	GCF_001293085.1	5127	3642.21733	P. aeruginosa
GCF_004102665.1	GCF_000496605.2	5127	3642.21733	P. aeruginosa
GCF_900497025.1	GCF_904866275.1_MINF_7A_genomic	5127	3642.21733	P. aeruginosa
GCF_000981825.1	GCF_904866275.1_MINF_7A_genomic	5128	3642.92773	P. aeruginosa
GCF_001293085.1	GCF_012935295.1	5130	3644.34852	P. aeruginosa
GCF_000496605.2	GCF_012935295.1	5130	3644.34852	P. aeruginosa
GCF_900497025.1	GCF_000026645.1	5130	3644.34852	P. aeruginosa
GCF_000981825.1	GCF_000026645.1	5131	3645.05892	P. aeruginosa
GCF_001293085.1	GCF_003571505.1	5135	3647.90052	P. aeruginosa
GCF_000496605.2	GCF_003571505.1	5135	3647.90052	P. aeruginosa
GCF_001293085.1	GCF_000568855.2	5135	3647.90052	P. aeruginosa
GCF_000496605.2	GCF_000568855.2	5135	3647.90052	P. aeruginosa
GCF_900497025.1	GCF_008033725.1	5162	3667.0813	P. aeruginosa
GCF_000981825.1	GCF_008033725.1	5163	3667.7917	P. aeruginosa
GCF_900497025.1	GCF_016126955.1	5166	3669.9229	P. aeruginosa
GCF_000981825.1	GCF_016126955.1	5167	3670.6333	P. aeruginosa
GCF_019466145.1	GCF_000271985.2	5168	3671.3437	P. aeruginosa
GCF_019466145.1	GCF_001293085.1	5171	3673.4749	P. aeruginosa
GCF_019466145.1	GCF_000496605.2	5171	3673.4749	P. aeruginosa
GCF_001293085.1	GCF_900636735.1_43941_C01_genomic	5187	3684.84129	P. aeruginosa
GCF_000496605.2	GCF_900636735.1_43941_C01_genomic	5187	3684.84129	P. aeruginosa
GCF_900497025.1	GCF_001879525.1	5189	3686.26208	P. aeruginosa
GCF_000981825.1	GCF_001879525.1	5190	3686.97248	P. aeruginosa
GCF_001293085.1	GCF_014854655.1	5197	3691.94528	P. aeruginosa
GCF_000496605.2	GCF_014854655.1	5197	3691.94528	P. aeruginosa
GCF_001293085.1	GCF_000414035.1	5197	3691.94528	P. aeruginosa
GCF_000496605.2	GCF_000414035.1	5197	3691.94528	P. aeruginosa
GCF_013282235.1	GCF_008432465.1	5212	7340.70032	A. xylosoxidans/A. insolitus
GCF_001293085.1	GCF_001045685.1	5223	3710.41566	P. aeruginosa
GCF_000496605.2	GCF_001045685.1	5223	3710.41566	P. aeruginosa
GCF_900497025.1	GCF_001606045.1	5236	3719.65085	P. aeruginosa
GCF_000981825.1	GCF_001606045.1	5237	3720.36125	P. aeruginosa
GCF_019466145.1	GCF_016105505.1	5257	3734.56924	P. aeruginosa
GCF_001293085.1	GCF_000271985.2	5259	3735.99004	P. aeruginosa
GCF_000496605.2	GCF_000271985.2	5259	3735.99004	P. aeruginosa
GCF_900497025.1	GCF_001900195.1	5271	3744.51483	P. aeruginosa
GCF_000981825.1	GCF_001900195.1	5272	3745.22523	P. aeruginosa
GCF_900497025.1	GCF_018409365.1	5280	3750.90842	P. aeruginosa
GCF_000981825.1	GCF_018409365.1	5281	3751.61882	P. aeruginosa
GCF_900497025.1	GCF_001045685.1	5286	3755.17082	P. aeruginosa
GCF_000981825.1	GCF_001045685.1	5287	3755.88122	P. aeruginosa
GCF_900497025.1	GCF_000414035.1	5297	3762.98521	P. aeruginosa
GCF_013282235.1	GCF_001457475.1_NCTC10807_genomic	5298	7461.8247	A. xylosoxidans/A. insolitus
GCF_000981825.1	GCF_000414035.1	5298	3763.69561	P. aeruginosa
GCF_013282235.1	GCF_900009115.1_BN2877_genomic	5300	7464.64154	A. xylosoxidans/A. insolitus
GCF_900243355.1_RW109_genomic	GCF_001293085.1	5300	3765.11641	P. aeruginosa
GCF_900243355.1_RW109_genomic	GCF_000496605.2	5300	3765.11641	P. aeruginosa
GCF_001293085.1	GCF_014792125.1	5310	3772.2204	P. aeruginosa
GCF_000496605.2	GCF_014792125.1	5310	3772.2204	P. aeruginosa
GCF_900497025.1	GCF_003319235.1	5324	3782.16599	P. aeruginosa
GCF_000981825.1	GCF_003319235.1	5325	3782.87639	P. aeruginosa
GCF_900497025.1	GCF_001750705.1	5329	3785.71799	P. aeruginosa
GCF_000981825.1	GCF_001750705.1	5330	3786.42839	P. aeruginosa
GCF_001293085.1	GCF_016105505.1	5340	3793.53238	P. aeruginosa
GCF_000496605.2	GCF_016105505.1	5340	3793.53238	P. aeruginosa
GCF_013282235.1	GCF_000508285.1	5342	7523.79531	A. xylosoxidans/A. insolitus
GCF_900497025.1	GCF_014792125.1	5349	3799.92598	P. aeruginosa
GCF_000981825.1	GCF_014792125.1	5350	3800.63638	P. aeruginosa
GCF_900497025.1	GCF_009911735.1	5392	3830.47315	P. aeruginosa
GCF_000981825.1	GCF_009911735.1	5393	3831.18355	P. aeruginosa
GCF_900497025.1	GCF_009648875.1	5403	3838.28754	P. aeruginosa
GCF_000981825.1	GCF_009648875.1	5404	3838.99794	P. aeruginosa
GCF_000967095.2_A.x.NH44784-1996_genomic	GCF_900009115.1_BN2877_genomic	5406	7613.93437	A. xylosoxidans/A. insolitus
GCF_900070375.1_PAO1OR_genomic	GCF_900497025.1	5423	3852.49553	P. aeruginosa
GCF_013305765.1	GCF_900497025.1	5423	3852.49553	P. aeruginosa
GCF_900070375.1_PAO1OR_genomic	GCF_000981825.1	5424	3853.20593	P. aeruginosa
GCF_013305765.1	GCF_000981825.1	5424	3853.20593	P. aeruginosa
GCF_004014755.1	GCF_900497025.1	5426	3854.62672	P. aeruginosa

GCF_004014755.1	GCF_000981825.1	5427	3855.33712	P. aeruginosa
GCF_014216355.1	GCF_003017885.1	5431	5356.02106	E. coli
GCF_004102665.1	GCF_900497025.1	5433	3859.59952	P. aeruginosa
GCF_900497025.1	GCF_000524595.1	5433	3859.59952	P. aeruginosa
GCF_004102665.1	GCF_000981825.1	5434	3860.30992	P. aeruginosa
GCF_000981825.1	GCF_000524595.1	5434	3860.30992	P. aeruginosa
GCF_900497025.1	GCF_000271985.2	5460	3878.7803	P. aeruginosa
GCF_000981825.1	GCF_000271985.2	5461	3879.4907	P. aeruginosa
GCF_900497025.1	GCF_000568855.2	5468	3884.4635	P. aeruginosa
GCF_000981825.1	GCF_000568855.2	5469	3885.1739	P. aeruginosa
GCF_003367575.1	GCF_003017885.1	5484	5408.28936	E. coli
GCF_900497025.1	GCF_012935295.1	5519	3920.69386	P. aeruginosa
GCF_000981825.1	GCF_012935295.1	5520	3921.40426	P. aeruginosa
GCF_900497025.1	GCF_900636735.1_43941_C01_genomic	5575	3960.47622	P. aeruginosa
GCF_000981825.1	GCF_900636735.1_43941_C01_genomic	5576	3961.18662	P. aeruginosa
GCF_000967095.2_A.x.NH44784-1996_genomic	GCF_013282235.1	5580	7858.99996	A. xylosoxidans/A. insolitus
GCF_900497025.1	GCF_003204335.1	5617	3990.31299	P. aeruginosa
GCF_000981825.1	GCF_003204335.1	5622	3993.86499	P. aeruginosa
GCF_900497025.1	GCF_002968515.1	5623	3994.57539	P. aeruginosa
GCF_000981825.1	GCF_002968515.1	5624	3995.28579	P. aeruginosa
GCF_001722005.2	GCF_001874465.1	5637	4004.52098	P. aeruginosa
GCF_000967095.2_A.x.NH44784-1996_genomic	GCF_008432465.1	5645	7950.54745	A. xylosoxidans/A. insolitus
GCF_000284555.1	GCF_001874465.1	5661	4021.57057	P. aeruginosa
GCF_902164685.1_25426_7_120_genomic	GCF_012955465.1	5677	5598.62485	E. coli
GCF_900497025.1	GCF_016105505.1	5688	4040.75135	P. aeruginosa
GCF_000981825.1	GCF_016105505.1	5689	4041.46175	P. aeruginosa
GCF_000967095.2_A.x.NH44784-1996_genomic	GCF_001457475.1_NCTC10807_genomic	5722	8058.99602	A. xylosoxidans/A. insolitus
GCF_900497025.1	GCF_019466145.1	5745	4081.24411	P. aeruginosa
GCF_014216355.1	GCF_016801475.1	5746	5666.67226	E. coli
GCF_000981825.1	GCF_019466145.1	5746	4081.95451	P. aeruginosa
GCF_004358945.1	GCF_018208275.1	5753	5673.57561	E. coli
GCF_000967095.2_A.x.NH44784-1996_genomic	GCF_000508285.1	5766	8120.96663	A. xylosoxidans/A. insolitus
GCF_014216355.1	GCF_003666405.1	5775	5695.27189	E. coli
GCF_900095805.1_PA14Or_genomic	GCF_001874465.1	5778	4104.68729	P. aeruginosa
GCF_002223805.1	GCF_001874465.1	5786	4110.37048	P. aeruginosa
GCF_000014625.1	GCF_001874465.1	5789	4112.50168	P. aeruginosa
GCF_003367575.1	GCF_003666405.1	5799	5718.94055	E. coli
GCF_003367575.1	GCF_016801475.1	5802	5721.89913	E. coli
GCF_001722005.2	GCF_000284555.1	5829	4140.91765	P. aeruginosa
GCF_002287725.2	GCF_001874465.1	5836	4145.89045	P. aeruginosa
GCF_900095805.1_PA14Or_genomic	GCF_001722005.2	5856	4160.09843	P. aeruginosa
GCF_000014625.1	GCF_001722005.2	5867	4167.91283	P. aeruginosa
GCF_002223805.1	GCF_001722005.2	5881	4177.85842	P. aeruginosa
GCF_018986775.1	GCF_012955465.1	5888	5806.71184	E. coli
GCF_014216355.1	GCF_003112045.1	5905	5823.47714	E. coli
GCF_014216355.1	GCF_003072445.1	5925	5843.20103	E. coli
GCF_002634895.1	GCF_018208275.1	5931	5849.11819	E. coli
GCF_003367575.1	GCF_003072445.1	5943	5860.95253	E. coli
GCF_014216355.1	GCF_002736085.1	5951	5868.84208	E. coli
GCF_900095805.1_PA14Or_genomic	GCF_002287725.2	5951	4227.58637	P. aeruginosa
GCF_900497025.1	GCF_001293085.1	5952	4228.29677	P. aeruginosa
GCF_900497025.1	GCF_000496605.2	5952	4228.29677	P. aeruginosa
GCF_000981825.1	GCF_001293085.1	5953	4229.00717	P. aeruginosa
GCF_000981825.1	GCF_000496605.2	5953	4229.00717	P. aeruginosa
GCF_000166535.2	GCF_018208275.1	5960	5877.71783	E. coli
GCF_000014625.1	GCF_002287725.2	5962	4235.40076	P. aeruginosa
GCF_003367575.1	GCF_003112045.1	5965	5882.6488	E. coli
GCF_002223805.1	GCF_002287725.2	5976	4245.34635	P. aeruginosa
GCF_000166535.2	GCF_019614135.1	5998	5915.19321	E. coli
GCF_003367575.1	GCF_002736085.1	6008	5925.05515	E. coli
GCF_002287725.2	GCF_000284555.1	6023	4278.73512	P. aeruginosa
GCF_900095805.1_PA14Or_genomic	GCF_000284555.1	6111	4341.25026	P. aeruginosa
GCF_000014625.1	GCF_000284555.1	6122	4349.06465	P. aeruginosa
GCF_002223805.1	GCF_000284555.1	6135	4358.29984	P. aeruginosa
GCF_902164685.1_25426_7_120_genomic	GCF_018986775.1	6268	6181.46566	E. coli
GCF_004358945.1	GCF_002879735.1	6335	6247.54068	E. coli
GCF_018517125.1	GCF_902164645.1_25426_7_121_genomic	6431	4014.71041	E. faecalis
GCF_002945235.1	GCF_018336495.1	6532	2769.97856	P. mirabilis
GCF_002879735.1	GCF_018208275.1	6566	6475.35155	E. coli
GCF_002634895.1	GCF_002879735.1	6668	6575.94337	E. coli
GCF_010442675.1	GCF_009429045.2	6710	2845.46174	P. mirabilis
GCF_003112045.1	GCF_019614135.1	6748	6654.83891	E. coli
GCF_002125925.1	GCF_002634895.1	6751	6657.79749	E. coli
GCF_003666405.1	GCF_019614135.1	6752	6658.78369	E. coli
GCF_002125925.1	GCF_004358945.1	6811	6716.96915	E. coli
GCF_000166535.2	GCF_002879735.1	6867	6772.19603	E. coli
GCF_016801475.1	GCF_019614135.1	6869	6774.16842	E. coli
GCF_003072445.1	GCF_019614135.1	6888	6792.90611	E. coli
GCF_003017885.1	GCF_019614135.1	6947	6851.09157	E. coli
GCF_000550745.1	GCF_902164645.1_25426_7_121_genomic	6975	4354.31583	E. faecalis
GCF_002736085.1	GCF_019614135.1	6981	6884.62217	E. coli
GCF_014216355.1	GCF_019614135.1	7137	7038.46848	E. coli
GCF_003367575.1	GCF_019614135.1	7193	7093.69536	E. coli
GCF_019614135.1	GCF_002879735.1	7270	7169.63232	E. coli
GCF_019614135.1	GCF_018208275.1	7322	7220.91442	E. coli
GCF_000444425.1	GCF_010442675.1	7410	3142.30574	P. mirabilis
GCF_003028695.1	GCF_012955465.1	7456	7353.06445	E. coli
GCF_003999775.1	GCF_012955465.1	7463	7359.96781	E. coli
GCF_013344585.1	GCF_012955465.1	7471	7367.85736	E. coli
GCF_002125925.1	GCF_019614135.1	7503	7399.41558	E. coli
GCF_003028695.1	GCF_018986775.1	7736	7629.19885	E. coli
GCF_003999775.1	GCF_018986775.1	7737	7630.18504	E. coli
GCF_013344585.1	GCF_018986775.1	7751	7643.99176	E. coli
GCF_003028695.1	GCF_902164685.1_25426_7_120_genomic	7779	7671.6052	E. coli

GCF_003999775.1	GCF_902164685.1_25426_7_120_genomic	7784	7676.53617	E. coli
GCF_002208945.1	GCF_018517125.1	7790	4863.09969	E. faecalis
GCF_013344585.1	GCF_902164685.1_25426_7_120_genomic	7794	7686.39811	E. coli
GCF_002125925.1	GCF_000166535.2	7816	7708.09439	E. coli
GCF_000444425.1	GCF_009429045.2	7819	3315.74745	P. mirabilis
GCF_000017205.1	GCF_016743035.1	7862	5585.15947	P. aeruginosa
GCF_000017205.1	GCF_006971785.1	7894	5607.89225	P. aeruginosa
GCF_000017205.1	GCF_003025345.2	7983	5671.11779	P. aeruginosa
GCF_014216355.1	GCF_004358945.1	8011	7900.40227	E. coli
GCF_003367575.1	GCF_004358945.1	8019	7908.29182	E. coli
GCF_008195605.1	GCF_010442675.1	8034	3406.92096	P. mirabilis
GCF_003666405.1	GCF_002879735.1	8124	8011.84222	E. coli
GCF_003112045.1	GCF_002879735.1	8166	8053.26238	E. coli
GCF_003112045.1	GCF_004358945.1	8200	8086.79299	E. coli
GCF_003112045.1	GCF_018208275.1	8285	8170.6195	E. coli
GCF_014216355.1	GCF_018208275.1	8288	8173.57808	E. coli
GCF_008195605.1	GCF_000444425.1	8293	3516.75324	P. mirabilis
GCF_003367575.1	GCF_018208275.1	8318	8203.16391	E. coli
GCF_003666405.1	GCF_004358945.1	8334	8218.94302	E. coli
GCF_003666405.1	GCF_018208275.1	8351	8235.70832	E. coli
GCF_003072445.1	GCF_002879735.1	8355	8239.6531	E. coli
GCF_014216355.1	GCF_002634895.1	8389	8273.1837	E. coli
GCF_008195605.1	GCF_009429045.2	8391	3558.3114	P. mirabilis
GCF_014216355.1	GCF_002879735.1	8405	8288.96281	E. coli
GCF_007632055.1	GCF_018986755.2	8419	5255.76845	E. faecalis
GCF_003367575.1	GCF_002634895.1	8438	8321.50722	E. coli
GCF_003072445.1	GCF_018208275.1	8448	8331.36916	E. coli
GCF_003367575.1	GCF_002879735.1	8463	8346.16208	E. coli
GCF_000550745.1	GCF_018517125.1	8475	5290.72784	E. faecalis
GCF_002736085.1	GCF_002879735.1	8477	8359.9688	E. coli
GCF_003112045.1	GCF_000166535.2	8485	8367.85835	E. coli
GCF_000391485.2	GCF_018986755.2	8489	5299.46768	E. faecalis
GCF_014216355.1	GCF_000166535.2	8490	8372.78932	E. coli
GCF_003017885.1	GCF_002879735.1	8503	8385.60985	E. coli
GCF_002736085.1	GCF_018208275.1	8509	8391.52701	E. coli
GCF_003367575.1	GCF_000166535.2	8512	8394.4856	E. coli
GCF_003017885.1	GCF_004358945.1	8528	8410.2647	E. coli
GCF_003072445.1	GCF_004358945.1	8535	8417.16806	E. coli
GCF_002736085.1	GCF_004358945.1	8584	8465.49158	E. coli
GCF_003666405.1	GCF_000166535.2	8596	8477.32591	E. coli
GCF_003017885.1	GCF_018208275.1	8614	8495.07741	E. coli
GCF_002736085.1	GCF_000166535.2	8629	8509.87033	E. coli
GCF_016801475.1	GCF_002879735.1	8636	8516.77369	E. coli
GCF_001874465.1	GCF_019466145.1	8673	6161.29332	P. aeruginosa
GCF_003666405.1	GCF_002634895.1	8682	8562.13862	E. coli
GCF_003112045.1	GCF_002634895.1	8683	8563.12482	E. coli
GCF_013407495.1	GCF_019711255.1	8696	2900.72038	K. pneumoniae
GCF_000284555.1	GCF_001293085.1	8736	6206.04848	P. aeruginosa
GCF_000284555.1	GCF_000496605.2	8736	6206.04848	P. aeruginosa
GCF_003017885.1	GCF_000166535.2	8752	8631.17222	E. coli
GCF_001874465.1	GCF_001293085.1	8753	6218.12527	P. aeruginosa
GCF_001874465.1	GCF_000496605.2	8753	6218.12527	P. aeruginosa
GCF_000284555.1	GCF_019466145.1	8760	6223.09807	P. aeruginosa
GCF_003072445.1	GCF_000166535.2	8762	8641.03416	E. coli
GCF_016801475.1	GCF_018208275.1	8817	8695.27485	E. coli
GCF_001874465.1	GCF_002968515.1	8825	6269.27402	P. aeruginosa
GCF_003072445.1	GCF_002634895.1	8826	8704.1506	E. coli
GCF_001874465.1	GCF_003204335.1	8826	6269.98442	P. aeruginosa
GCF_001874465.1	GCF_900243355.1_RW109_genomic	8833	6274.95722	P. aeruginosa
GCF_000284555.1	GCF_001045685.1	8833	6274.95722	P. aeruginosa
GCF_001874465.1	GCF_001045685.1	8849	6286.32361	P. aeruginosa
GCF_001874465.1	GCF_009648875.1	8869	6300.53159	P. aeruginosa
GCF_019711255.1	GCF_019284495.1	8880	2962.09716	K. pneumoniae
GCF_016801475.1	GCF_004358945.1	8906	8783.04614	E. coli
GCF_001722005.2	GCF_019466145.1	8908	6328.23717	P. aeruginosa
GCF_001874465.1	GCF_000568855.2	8909	6328.94756	P. aeruginosa
GCF_000284555.1	GCF_009648875.1	8919	6336.05156	P. aeruginosa
GCF_001722005.2	GCF_001293085.1	8924	6339.60355	P. aeruginosa
GCF_001722005.2	GCF_000496605.2	8924	6339.60355	P. aeruginosa
GCF_014893535.1	GCF_000550745.1	8930	5574.77281	E. faecalis
GCF_001874465.1	GCF_000414035.1	8931	6344.57635	P. aeruginosa
GCF_000284555.1	GCF_002968515.1	8933	6345.99715	P. aeruginosa
GCF_000284555.1	GCF_003204335.1	8933	6345.99715	P. aeruginosa
GCF_002736085.1	GCF_002634895.1	8935	8811.64577	E. coli
GCF_001874465.1	GCF_018409365.1	8937	6348.83875	P. aeruginosa
GCF_001874465.1	GCF_000271985.2	8948	6356.65314	P. aeruginosa
GCF_003017885.1	GCF_002634895.1	8953	8829.39727	E. coli
GCF_000284555.1	GCF_016105505.1	8958	6363.75713	P. aeruginosa
GCF_000284555.1	GCF_900243355.1_RW109_genomic	8960	6365.17793	P. aeruginosa
GCF_001722005.2	GCF_001045685.1	8960	6365.17793	P. aeruginosa
GCF_000284555.1	GCF_000524595.1	8963	6367.30913	P. aeruginosa
GCF_001874465.1	GCF_016105505.1	8967	6370.15073	P. aeruginosa
GCF_001874465.1	GCF_003571505.1	8971	6372.99232	P. aeruginosa
GCF_000284555.1	GCF_014792125.1	8972	6373.70272	P. aeruginosa
GCF_001874465.1	GCF_012935295.1	8981	6380.09632	P. aeruginosa
GCF_000284555.1	GCF_000271985.2	8985	6382.93791	P. aeruginosa
GCF_000284555.1	GCF_000414035.1	8992	6387.91071	P. aeruginosa
GCF_000284555.1	GCF_016126955.1	8996	6390.75231	P. aeruginosa
GCF_000284555.1	GCF_000568855.2	9000	6393.5939	P. aeruginosa
GCF_000284555.1	GCF_003571505.1	9005	6397.1459	P. aeruginosa
GCF_001874465.1	GCF_000524595.1	9005	6397.1459	P. aeruginosa
GCF_001722005.2	GCF_000271985.2	9008	6399.2771	P. aeruginosa
GCF_000284555.1	GCF_001606045.1	9009	6399.9875	P. aeruginosa
GCF_001874465.1	GCF_014792125.1	9011	6401.4083	P. aeruginosa

GCF_001874465.1	GCF_016126955.1	9012	6402.11869	P. aeruginosa
GCF_001874465.1	GCF_014854655.1	9015	6404.24989	P. aeruginosa
GCF_001874465.1	GCF_001606045.1	9024	6410.64349	P. aeruginosa
GCF_000284555.1	GCF_900070375.1_PAO1OR_genomic	9025	6411.35389	P. aeruginosa
GCF_000284555.1	GCF_013305765.1	9025	6411.35389	P. aeruginosa
GCF_001722005.2	GCF_002968515.1	9027	6412.77468	P. aeruginosa
GCF_000284555.1	GCF_004014755.1	9028	6413.48508	P. aeruginosa
GCF_001722005.2	GCF_003204335.1	9029	6414.19548	P. aeruginosa
GCF_001874465.1	GCF_009911735.1	9032	6416.32668	P. aeruginosa
GCF_000284555.1	GCF_004102665.1	9035	6418.45788	P. aeruginosa
GCF_000284555.1	GCF_018409365.1	9037	6419.87868	P. aeruginosa
GCF_000284555.1	GCF_900636735.1_43941_C01_genomic	9040	6422.00988	P. aeruginosa
GCF_001874465.1	GCF_008033725.1	9046	6426.27227	P. aeruginosa
GCF_000284555.1	GCF_014854655.1	9048	6427.69307	P. aeruginosa
GCF_000284555.1	GCF_900149285.1_PcyII-10_genomic	9048	6427.69307	P. aeruginosa
GCF_001874465.1	GCF_001879525.1	9049	6428.40347	P. aeruginosa
GCF_000284555.1	GCF_904866275.1_MINF_7A_genomic	9049	6428.40347	P. aeruginosa
GCF_900095805.1_PA14Or_genomic	GCF_001293085.1	9051	6429.82427	P. aeruginosa
GCF_900095805.1_PA14Or_genomic	GCF_000496605.2	9051	6429.82427	P. aeruginosa
GCF_000284555.1	GCF_009911735.1	9056	6433.37627	P. aeruginosa
GCF_000240185.1	GCF_019711255.1	9058	3021.47254	K. pneumoniae
GCF_000014625.1	GCF_001293085.1	9062	6437.63866	P. aeruginosa
GCF_000014625.1	GCF_000496605.2	9062	6437.63866	P. aeruginosa
GCF_000284555.1	GCF_012935295.1	9063	6438.34906	P. aeruginosa
GCF_001874465.1	GCF_900149285.1_PcyII-10_genomic	9064	6439.05946	P. aeruginosa
GCF_001874465.1	GCF_003319235.1	9066	6440.48026	P. aeruginosa
GCF_001874465.1	GCF_001750705.1	9069	6442.61146	P. aeruginosa
GCF_002223805.1	GCF_001293085.1	9076	6447.58425	P. aeruginosa
GCF_002223805.1	GCF_000496605.2	9076	6447.58425	P. aeruginosa
GCF_000284555.1	GCF_008033725.1	9080	6450.42585	P. aeruginosa
GCF_001722005.2	GCF_009648875.1	9085	6453.97785	P. aeruginosa
GCF_001874465.1	GCF_904866275.1_MINF_7A_genomic	9085	6453.97785	P. aeruginosa
GCF_001874465.1	GCF_900070375.1_PAO1OR_genomic	9091	6458.24024	P. aeruginosa
GCF_001874465.1	GCF_013305765.1	9091	6458.24024	P. aeruginosa
GCF_001874465.1	GCF_004014755.1	9094	6460.37144	P. aeruginosa
GCF_001874465.1	GCF_000226155.1	9094	6460.37144	P. aeruginosa
GCF_000284555.1	GCF_003319235.1	9097	6462.50264	P. aeruginosa
GCF_000284555.1	GCF_001750705.1	9100	6464.63384	P. aeruginosa
GCF_001874465.1	GCF_004102665.1	9101	6465.34423	P. aeruginosa
GCF_001874465.1	GCF_001900195.1	9105	6468.18583	P. aeruginosa
GCF_001874465.1	GCF_900636735.1_43941_C01_genomic	9108	6470.31703	P. aeruginosa
GCF_000284555.1	GCF_001879525.1	9124	6481.68342	P. aeruginosa
GCF_001722005.2	GCF_012935295.1	9124	6481.68342	P. aeruginosa
GCF_001722005.2	GCF_900243355.1_RW109_genomic	9128	6484.52502	P. aeruginosa
GCF_001722005.2	GCF_001606045.1	9128	6484.52502	P. aeruginosa
GCF_900095805.1_PA14Or_genomic	GCF_019466145.1	9129	6485.23542	P. aeruginosa
GCF_000284555.1	GCF_001900195.1	9132	6487.36661	P. aeruginosa
GCF_016801475.1	GCF_000166535.2	9135	9008.88462	E. coli
GCF_001722005.2	GCF_003571505.1	9136	6490.20821	P. aeruginosa
GCF_000014625.1	GCF_019466145.1	9140	6493.04981	P. aeruginosa
GCF_001722005.2	GCF_016126955.1	9140	6493.04981	P. aeruginosa
GCF_001722005.2	GCF_016105505.1	9149	6499.4434	P. aeruginosa
GCF_001722005.2	GCF_008033725.1	9151	6500.8642	P. aeruginosa
GCF_001874465.1	GCF_000026645.1	9153	6502.285	P. aeruginosa
GCF_003194125.1	GCF_004138625.1	9154	9027.62232	E. coli
GCF_002223805.1	GCF_019466145.1	9154	6502.9954	P. aeruginosa
GCF_000284555.1	GCF_000226155.1	9154	6502.9954	P. aeruginosa
GCF_002287725.2	GCF_001293085.1	9165	6510.80979	P. aeruginosa
GCF_002287725.2	GCF_000496605.2	9165	6510.80979	P. aeruginosa
GCF_001722005.2	GCF_014792125.1	9165	6510.80979	P. aeruginosa
GCF_002287725.2	GCF_019466145.1	9170	6514.36179	P. aeruginosa
GCF_001722005.2	GCF_018409365.1	9170	6514.36179	P. aeruginosa
GCF_010103655.1	GCF_014893535.1	9171	5725.22301	E. faecalis
GCF_001722005.2	GCF_000414035.1	9172	6515.78259	P. aeruginosa
GCF_016801475.1	GCF_002634895.1	9175	9048.33239	E. coli
GCF_001722005.2	GCF_000568855.2	9175	6517.91378	P. aeruginosa
GCF_006349345.1	GCF_014893535.1	9184	5733.33858	E. faecalis
GCF_012594215.1	GCF_014893535.1	9185	5733.96285	E. faecalis
GCF_001722005.2	GCF_014854655.1	9186	6525.72818	P. aeruginosa
GCF_000284555.1	GCF_000026645.1	9186	6525.72818	P. aeruginosa
GCF_009684615.1	GCF_019711255.1	9197	3067.8387	K. pneumoniae
GCF_018138945.1	GCF_003030945.1	9200	3901.37825	P. mirabilis
GCF_001006265.1	GCF_019711255.1	9220	3075.51079	K. pneumoniae
GCF_001722005.2	GCF_904866275.1_MINF_7A_genomic	9226	6554.14415	P. aeruginosa
GCF_001722005.2	GCF_003319235.1	9231	6557.69615	P. aeruginosa
GCF_001722005.2	GCF_000026645.1	9231	6557.69615	P. aeruginosa
GCF_002287725.2	GCF_001045685.1	9233	6559.11695	P. aeruginosa
GCF_001722005.2	GCF_001750705.1	9234	6559.82734	P. aeruginosa
GCF_001722005.2	GCF_009911735.1	9246	6568.35214	P. aeruginosa
GCF_002125925.1	GCF_018208275.1	9249	9121.31077	E. coli
GCF_001722005.2	GCF_900636735.1_43941_C01_genomic	9250	6571.19373	P. aeruginosa
GCF_001722005.2	GCF_001879525.1	9253	6573.32493	P. aeruginosa
GCF_002287725.2	GCF_000271985.2	9253	6573.32493	P. aeruginosa
GCF_001722005.2	GCF_900070375.1_PAO1OR_genomic	9259	6577.58733	P. aeruginosa
GCF_001722005.2	GCF_013305765.1	9259	6577.58733	P. aeruginosa
GCF_900095805.1_PA14Or_genomic	GCF_002968515.1	9260	6578.29773	P. aeruginosa
GCF_003815075.1	GCF_019711255.1	9261	3089.18714	K. pneumoniae
GCF_900095805.1_PA14Or_genomic	GCF_003204335.1	9261	6579.00813	P. aeruginosa
GCF_001722005.2	GCF_004014755.1	9262	6579.71853	P. aeruginosa
GCF_001722005.2	GCF_000524595.1	9264	6581.13932	P. aeruginosa
GCF_001722005.2	GCF_004102665.1	9269	6584.69132	P. aeruginosa
GCF_000014625.1	GCF_002968515.1	9271	6586.11212	P. aeruginosa
GCF_001722005.2	GCF_000226155.1	9271	6586.11212	P. aeruginosa
GCF_000014625.1	GCF_003204335.1	9272	6586.82252	P. aeruginosa

GCF_001722005.2	GCF_900149285.1_PcyII-10_genomic	9282	6593.92651	P. aeruginosa
GCF_900095805.1_PA14Or_genomic	GCF_001045685.1	9283	6594.63691	P. aeruginosa
GCF_002223805.1	GCF_002968515.1	9285	6596.05771	P. aeruginosa
GCF_002223805.1	GCF_003204335.1	9286	6596.76811	P. aeruginosa
GCF_002287725.2	GCF_002968515.1	9287	6597.47851	P. aeruginosa
GCF_002287725.2	GCF_003204335.1	9287	6597.47851	P. aeruginosa
GCF_000014625.1	GCF_001045685.1	9294	6602.4513	P. aeruginosa
GCF_002211665.1	GCF_019711255.1	9301	3102.52992	K. pneumoniae
GCF_002223805.1	GCF_001045685.1	9308	6612.39689	P. aeruginosa
GCF_001722005.2	GCF_001900195.1	9326	6625.18408	P. aeruginosa
GCF_900095805.1_PA14Or_genomic	GCF_000271985.2	9331	6628.73608	P. aeruginosa
GCF_002287725.2	GCF_009648875.1	9335	6631.57768	P. aeruginosa
GCF_000014625.1	GCF_000271985.2	9342	6636.55047	P. aeruginosa
GCF_002223805.1	GCF_000271985.2	9355	6645.78566	P. aeruginosa
GCF_002287725.2	GCF_003571505.1	9368	6655.02085	P. aeruginosa
GCF_002287725.2	GCF_016105505.1	9378	6662.12485	P. aeruginosa
GCF_002287725.2	GCF_900243355.1_RW109_genomic	9384	6666.38724	P. aeruginosa
GCF_002208945.1	GCF_902164645.1_25426_7_121_genomic	9387	5860.06634	E. faecalis
GCF_002287725.2	GCF_001606045.1	9392	6672.07044	P. aeruginosa
GCF_900095805.1_PA14Or_genomic	GCF_016105505.1	9394	6673.49124	P. aeruginosa
GCF_900095805.1_PA14Or_genomic	GCF_012935295.1	9403	6679.88483	P. aeruginosa
GCF_000014625.1	GCF_016105505.1	9405	6681.30563	P. aeruginosa
GCF_002287725.2	GCF_012935295.1	9405	6681.30563	P. aeruginosa
GCF_001874465.1	GCF_900497025.1	9409	6684.14723	P. aeruginosa
GCF_001874465.1	GCF_000981825.1	9410	6684.85763	P. aeruginosa
GCF_900095805.1_PA14Or_genomic	GCF_009648875.1	9410	6684.85763	P. aeruginosa
GCF_002287725.2	GCF_016126955.1	9414	6687.69922	P. aeruginosa
GCF_000014625.1	GCF_012935295.1	9414	6687.69922	P. aeruginosa
GCF_002223805.1	GCF_016105505.1	9419	6691.25122	P. aeruginosa
GCF_000014625.1	GCF_009648875.1	9421	6692.67202	P. aeruginosa
GCF_002287725.2	GCF_000414035.1	9424	6694.80322	P. aeruginosa
GCF_002287725.2	GCF_014792125.1	9426	6696.22401	P. aeruginosa
GCF_002223805.1	GCF_012935295.1	9428	6697.64481	P. aeruginosa
GCF_002287725.2	GCF_000568855.2	9432	6700.48641	P. aeruginosa
GCF_002223805.1	GCF_009648875.1	9435	6702.61761	P. aeruginosa
GCF_002287725.2	GCF_014854655.1	9437	6704.03841	P. aeruginosa
GCF_002287725.2	GCF_018409365.1	9442	6707.5904	P. aeruginosa
GCF_900095805.1_PA14Or_genomic	GCF_000414035.1	9443	6708.3008	P. aeruginosa
GCF_900095805.1_PA14Or_genomic	GCF_000568855.2	9446	6710.432	P. aeruginosa
GCF_002287725.2	GCF_008033725.1	9447	6711.1424	P. aeruginosa
GCF_900095805.1_PA14Or_genomic	GCF_000524595.1	9448	6711.8528	P. aeruginosa
GCF_000014625.1	GCF_000414035.1	9454	6716.1152	P. aeruginosa
GCF_900095805.1_PA14Or_genomic	GCF_018409365.1	9457	6718.24639	P. aeruginosa
GCF_000014625.1	GCF_000568855.2	9457	6718.24639	P. aeruginosa
GCF_000014625.1	GCF_000524595.1	9459	6719.66719	P. aeruginosa
GCF_002208945.1	GCF_000550745.1	9464	5908.13549	E. faecalis
GCF_900095805.1_PA14Or_genomic	GCF_900636735.1_43941_C01_genomic	9465	6723.92959	P. aeruginosa
GCF_900095805.1_PA14Or_genomic	GCF_900243355.1_RW109_genomic	9467	6725.35039	P. aeruginosa
GCF_000014625.1	GCF_018409365.1	9468	6726.06079	P. aeruginosa
GCF_002223805.1	GCF_000414035.1	9468	6726.06079	P. aeruginosa
GCF_002223805.1	GCF_000568855.2	9471	6728.19198	P. aeruginosa
GCF_900069965.1_KPN_RH201207_genomic	GCF_019711255.1	9472	3159.57031	K. pneumoniae
GCF_002223805.1	GCF_000524595.1	9473	6729.61278	P. aeruginosa
GCF_002223805.1	GCF_900243355.1_RW109_genomic	9475	6731.03358	P. aeruginosa
GCF_000014625.1	GCF_900636735.1_43941_C01_genomic	9476	6731.74398	P. aeruginosa
GCF_000014625.1	GCF_900243355.1_RW109_genomic	9478	6733.16478	P. aeruginosa
GCF_002287725.2	GCF_904866275.1_MINF_7A_genomic	9481	6735.29598	P. aeruginosa
GCF_002223805.1	GCF_018409365.1	9482	6736.00638	P. aeruginosa
GCF_900095805.1_PA14Or_genomic	GCF_016126955.1	9483	6736.71678	P. aeruginosa
GCF_900095805.1_PA14Or_genomic	GCF_001606045.1	9485	6738.13757	P. aeruginosa
GCF_002287725.2	GCF_900636735.1_43941_C01_genomic	9486	6738.84797	P. aeruginosa
GCF_002223805.1	GCF_900636735.1_43941_C01_genomic	9490	6741.68957	P. aeruginosa
GCF_900095805.1_PA14Or_genomic	GCF_009911735.1	9492	6743.11037	P. aeruginosa
GCF_002287725.2	GCF_009911735.1	9493	6743.82077	P. aeruginosa
GCF_000014625.1	GCF_016126955.1	9494	6744.53117	P. aeruginosa
GCF_000014625.1	GCF_001606045.1	9496	6745.95197	P. aeruginosa
GCF_002287725.2	GCF_003319235.1	9499	6748.08317	P. aeruginosa
GCF_002287725.2	GCF_001750705.1	9502	6750.21436	P. aeruginosa
GCF_000014625.1	GCF_009911735.1	9503	6750.92476	P. aeruginosa
GCF_002287725.2	GCF_900070375.1_PAO1OR_genomic	9508	6754.47676	P. aeruginosa
GCF_002287725.2	GCF_013305765.1	9508	6754.47676	P. aeruginosa
GCF_002223805.1	GCF_016126955.1	9508	6754.47676	P. aeruginosa
GCF_002223805.1	GCF_001606045.1	9510	6755.89756	P. aeruginosa
GCF_002287725.2	GCF_004014755.1	9511	6756.60796	P. aeruginosa
GCF_900095805.1_PA14Or_genomic	GCF_008033725.1	9515	6759.44955	P. aeruginosa
GCF_002223805.1	GCF_009911735.1	9517	6760.87035	P. aeruginosa
GCF_002287725.2	GCF_004102665.1	9518	6761.58075	P. aeruginosa
GCF_000284555.1	GCF_900497025.1	9519	6762.29115	P. aeruginosa
GCF_000284555.1	GCF_000981825.1	9520	6763.00155	P. aeruginosa
GCF_001938625.2	GCF_007107705.1	9524	9392.51419	E. coli
GCF_900095805.1_PA14Or_genomic	GCF_003571505.1	9525	6766.55355	P. aeruginosa
GCF_000014625.1	GCF_008033725.1	9526	6767.26395	P. aeruginosa
GCF_002287725.2	GCF_000226155.1	9527	6767.97435	P. aeruginosa
GCF_002287725.2	GCF_000524595.1	9533	6772.23674	P. aeruginosa
GCF_900095805.1_PA14Or_genomic	GCF_014854655.1	9535	6773.65754	P. aeruginosa
GCF_000014625.1	GCF_003571505.1	9536	6774.36794	P. aeruginosa
GCF_900095805.1_PA14Or_genomic	GCF_003319235.1	9537	6775.07834	P. aeruginosa
GCF_900095805.1_PA14Or_genomic	GCF_001750705.1	9540	6777.20954	P. aeruginosa
GCF_002223805.1	GCF_008033725.1	9540	6777.20954	P. aeruginosa
GCF_900095805.1_PA14Or_genomic	GCF_904866275.1_MINF_7A_genomic	9540	6777.20954	P. aeruginosa
GCF_002287725.2	GCF_000026645.1	9541	6777.91994	P. aeruginosa
GCF_002287725.2	GCF_900149285.1_PcyII-10_genomic	9542	6778.63034	P. aeruginosa
GCF_000014625.1	GCF_014854655.1	9546	6781.47193	P. aeruginosa
GCF_000014625.1	GCF_003319235.1	9548	6782.89273	P. aeruginosa

GCF_002223805.1	GCF_003571505.1	9550	6784.31353	P. aeruginosa
GCF_000014625.1	GCF_001750705.1	9551	6785.02393	P. aeruginosa
GCF_900095805.1_PA14Or_genomic	GCF_900149285.1_PcyII-10_genomic	9551	6785.02393	P. aeruginosa
GCF_000014625.1	GCF_904866275.1_MINF_7A_genomic	9551	6785.02393	P. aeruginosa
GCF_002287725.2	GCF_001879525.1	9553	6786.44473	P. aeruginosa
GCF_900095805.1_PA14Or_genomic	GCF_900070375.1_PAO1OR_genomic	9558	6789.99673	P. aeruginosa
GCF_900095805.1_PA14Or_genomic	GCF_013305765.1	9558	6789.99673	P. aeruginosa
GCF_014893535.1	GCF_902164645.1_25426_7_121_genomic	9560	5968.06585	E. faecalis
GCF_002223805.1	GCF_014854655.1	9560	6791.41752	P. aeruginosa
GCF_900095805.1_PA14Or_genomic	GCF_004014755.1	9561	6792.12792	P. aeruginosa
GCF_002223805.1	GCF_003319235.1	9562	6792.83832	P. aeruginosa
GCF_000014625.1	GCF_900149285.1_PcyII-10_genomic	9562	6792.83832	P. aeruginosa
GCF_002223805.1	GCF_001750705.1	9565	6794.96952	P. aeruginosa
GCF_002223805.1	GCF_904866275.1_MINF_7A_genomic	9565	6794.96952	P. aeruginosa
GCF_900095805.1_PA14Or_genomic	GCF_004102665.1	9568	6797.10072	P. aeruginosa
GCF_000014625.1	GCF_900070375.1_PAO1OR_genomic	9569	6797.81112	P. aeruginosa
GCF_000014625.1	GCF_013305765.1	9569	6797.81112	P. aeruginosa
GCF_900095805.1_PA14Or_genomic	GCF_014792125.1	9569	6797.81112	P. aeruginosa
GCF_900095805.1_PA14Or_genomic	GCF_001879525.1	9569	6797.81112	P. aeruginosa
GCF_000014625.1	GCF_004014755.1	9572	6799.94232	P. aeruginosa
GCF_002223805.1	GCF_900149285.1_PcyII-10_genomic	9576	6802.78391	P. aeruginosa
GCF_900095805.1_PA14Or_genomic	GCF_000026645.1	9577	6803.49431	P. aeruginosa
GCF_000014625.1	GCF_004102665.1	9579	6804.91511	P. aeruginosa
GCF_000014625.1	GCF_014792125.1	9580	6805.62551	P. aeruginosa
GCF_000014625.1	GCF_001879525.1	9580	6805.62551	P. aeruginosa
GCF_002223805.1	GCF_900070375.1_PAO1OR_genomic	9583	6807.75671	P. aeruginosa
GCF_002223805.1	GCF_013305765.1	9583	6807.75671	P. aeruginosa
GCF_002223805.1	GCF_004014755.1	9586	6809.88791	P. aeruginosa
GCF_000014625.1	GCF_000026645.1	9588	6811.3087	P. aeruginosa
GCF_002223805.1	GCF_004102665.1	9593	6814.8607	P. aeruginosa
GCF_002223805.1	GCF_014792125.1	9594	6815.5711	P. aeruginosa
GCF_002223805.1	GCF_001879525.1	9594	6815.5711	P. aeruginosa
GCF_002223805.1	GCF_000026645.1	9602	6821.2543	P. aeruginosa
GCF_002287725.2	GCF_001900195.1	9612	6828.35829	P. aeruginosa
GCF_900095805.1_PA14Or_genomic	GCF_000226155.1	9615	6830.48949	P. aeruginosa
GCF_000014625.1	GCF_000226155.1	9626	6838.30388	P. aeruginosa
GCF_002223805.1	GCF_000226155.1	9640	6848.24947	P. aeruginosa
GCF_900095805.1_PA14Or_genomic	GCF_001900195.1	9645	6851.80147	P. aeruginosa
GCF_002125925.1	GCF_002879735.1	9652	9518.74706	E. coli
GCF_010103655.1	GCF_000550745.1	9653	6026.1234	E. faecalis
GCF_000014625.1	GCF_001900195.1	9656	6859.61586	P. aeruginosa
GCF_006349345.1	GCF_000550745.1	9662	6031.74187	E. faecalis
GCF_000240185.1	GCF_003815075.1	9664	3223.61565	K. pneumoniae
GCF_012594215.1	GCF_000550745.1	9667	6034.86324	E. faecalis
GCF_002223805.1	GCF_001900195.1	9669	6868.85105	P. aeruginosa
GCF_002211665.1	GCF_003815075.1	9681	3229.28633	K. pneumoniae
GCF_001722005.2	GCF_000981825.1	9685	6880.21744	P. aeruginosa
GCF_001722005.2	GCF_900497025.1	9690	6883.76944	P. aeruginosa
GCF_009684615.1	GCF_003815075.1	9713	3239.96056	K. pneumoniae
GCF_016859025.1	GCF_019711255.1	9768	3258.30688	K. pneumoniae
GCF_014893535.1	GCF_003966385.1	9799	6117.2675	E. faecalis
GCF_900069965.1_KPN_RH201207_genomic	GCF_003815075.1	9878	3294.99953	K. pneumoniae
GCF_014931585.1	GCF_011383025.1	9902	4199.07037	P. mirabilis
GCF_000069965.1	GCF_011383025.1	9905	4200.34256	P. mirabilis
GCF_002287725.2	GCF_900497025.1	9937	7059.23807	P. aeruginosa
GCF_002287725.2	GCF_000981825.1	9938	7059.94847	P. aeruginosa
GCF_010103655.1	GCF_902164645.1_25426_7_121_genomic	9945	6208.4116	E. faecalis
GCF_006349345.1	GCF_902164645.1_25426_7_121_genomic	9955	6214.65435	E. faecalis
GCF_012594215.1	GCF_902164645.1_25426_7_121_genomic	9959	6217.15145	E. faecalis
GCF_014931585.1	GCF_000069965.1	9971	4228.33071	P. mirabilis
GCF_900095805.1_PA14Or_genomic	GCF_900497025.1	10021	7118.91161	P. aeruginosa
GCF_900095805.1_PA14Or_genomic	GCF_000981825.1	10022	7119.62201	P. aeruginosa
GCF_000014625.1	GCF_900497025.1	10032	7126.726	P. aeruginosa
GCF_000014625.1	GCF_000981825.1	10033	7127.4364	P. aeruginosa
GCF_014898635.1	GCF_007107705.1	10043	9904.34902	E. coli
GCF_002223805.1	GCF_900497025.1	10046	7136.67159	P. aeruginosa
GCF_002223805.1	GCF_000981825.1	10047	7137.38199	P. aeruginosa
GCF_001640985.1	GCF_014931585.1	10094	4280.49044	P. mirabilis
GCF_003963495.1	GCF_019711255.1	10099	3368.71839	K. pneumoniae
GCF_013343255.1	GCF_000069965.1	10101	4283.45888	P. mirabilis
GCF_002208945.1	GCF_014893535.1	10108	6310.16837	E. faecalis
GCF_002310875.1	GCF_000069965.1	10117	4290.24388	P. mirabilis
GCF_001640985.1	GCF_000069965.1	10118	4290.66795	P. mirabilis
GCF_012594215.1	GCF_003966385.1	10119	6317.03539	E. faecalis
GCF_010103655.1	GCF_003966385.1	10130	6323.90242	E. faecalis
GCF_006349345.1	GCF_003966385.1	10133	6325.77524	E. faecalis
GCF_014898635.1	GCF_001938625.2	10151	10010.858	E. coli
GCF_014893535.1	GCF_018517125.1	10232	6387.57843	E. faecalis
GCF_013343255.1	GCF_014931585.1	10237	4341.13143	P. mirabilis
GCF_002310875.1	GCF_014931585.1	10253	4347.91643	P. mirabilis
GCF_007632055.1	GCF_010103655.1	10254	6401.31248	E. faecalis
GCF_007632055.1	GCF_006349345.1	10263	6406.93095	E. faecalis
GCF_007632055.1	GCF_012594215.1	10268	6410.05232	E. faecalis
GCF_010103655.1	GCF_001989555.1	10297	6428.15629	E. faecalis
GCF_013343255.1	GCF_011383025.1	10298	4366.99926	P. mirabilis
GCF_006349345.1	GCF_001989555.1	10308	6435.02331	E. faecalis
GCF_012594215.1	GCF_001989555.1	10309	6435.64758	E. faecalis
GCF_002310875.1	GCF_011383025.1	10314	4373.78426	P. mirabilis
GCF_000391485.2	GCF_010103655.1	10321	6443.13888	E. faecalis
GCF_000391485.2	GCF_006349345.1	10331	6449.38162	E. faecalis
GCF_000391485.2	GCF_012594215.1	10335	6451.87872	E. faecalis
GCF_007632055.1	GCF_014893535.1	10342	6456.24865	E. faecalis
GCF_010103655.1	GCF_018986755.2	10360	6467.48559	E. faecalis
GCF_006349345.1	GCF_018986755.2	10371	6474.35261	E. faecalis

GCF_012594215.1	GCF_018986755.2	10372	6474.97689	E. faecalis
GCF_000391485.2	GCF_014893535.1	10421	6505.56635	E. faecalis
GCF_001989555.1	GCF_014893535.1	10424	6507.43917	E. faecalis
GCF_001640985.1	GCF_011383025.1	10472	4440.7862	P. mirabilis
GCF_010103655.1	GCF_018517125.1	10477	6540.52573	E. faecalis
GCF_006349345.1	GCF_018517125.1	10489	6548.01702	E. faecalis
GCF_012594215.1	GCF_018517125.1	10491	6549.26557	E. faecalis
GCF_010103655.1	GCF_002208945.1	10498	6553.63549	E. faecalis
GCF_902164645.1_25426_7_121_genomic	GCF_003966385.1	10503	6556.75687	E. faecalis
GCF_006349345.1	GCF_002208945.1	10504	6557.38114	E. faecalis
GCF_012594215.1	GCF_002208945.1	10512	6562.37534	E. faecalis
GCF_018986755.2	GCF_014893535.1	10530	6573.61228	E. faecalis
GCF_000391485.2	GCF_000550745.1	10565	6595.4619	E. faecalis
GCF_007632055.1	GCF_001989555.1	10572	6599.83182	E. faecalis
GCF_002055685.1	GCF_008630655.1	10576	4484.88873	P. mirabilis
GCF_018138945.1	GCF_003073935.1	10589	4490.40155	P. mirabilis
GCF_000550745.1	GCF_003966385.1	10597	6615.43869	E. faecalis
GCF_001989555.1	GCF_902164645.1_25426_7_121_genomic	10629	6635.41548	E. faecalis
GCF_011383025.1	GCF_008630655.1	10629	4507.36406	P. mirabilis
GCF_000391485.2	GCF_001989555.1	10654	6651.02234	E. faecalis
GCF_014843115.1	GCF_008630655.1	10666	4523.05439	P. mirabilis
GCF_001989555.1	GCF_000550745.1	10668	6659.76219	E. faecalis
GCF_014931585.1	GCF_002055685.1	10711	4542.13722	P. mirabilis
GCF_014931585.1	GCF_008630655.1	10732	4551.04254	P. mirabilis
GCF_019443785.1	GCF_003073935.1	10734	4551.89066	P. mirabilis
GCF_007632055.1	GCF_902164645.1_25426_7_121_genomic	10759	6716.57118	E. faecalis
GCF_007632055.1	GCF_000550745.1	10805	6745.28782	E. faecalis
GCF_000391485.2	GCF_902164645.1_25426_7_121_genomic	10815	6751.53057	E. faecalis
GCF_018972025.1	GCF_008630655.1	10823	4589.63226	P. mirabilis
GCF_001989555.1	GCF_003966385.1	10824	6757.14904	E. faecalis
GCF_019443785.1	GCF_018138945.1	10839	4596.41726	P. mirabilis
GCF_018986755.2	GCF_902164645.1_25426_7_121_genomic	10870	6785.86567	E. faecalis
GCF_018986755.2	GCF_000550745.1	10872	6787.11422	E. faecalis
GCF_000069965.1	GCF_008630655.1	10872	4610.41134	P. mirabilis
GCF_000069965.1	GCF_002055685.1	10882	4614.65197	P. mirabilis
GCF_003112045.1	GCF_002125925.1	10895	10744.5865	E. coli
GCF_007632055.1	GCF_003966385.1	10895	6801.47254	E. faecalis
GCF_018336495.1	GCF_019443785.1	10914	4628.22198	P. mirabilis
GCF_001640985.1	GCF_019443785.1	10918	4629.91823	P. mirabilis
GCF_014843115.1	GCF_002055685.1	10939	4638.82355	P. mirabilis
GCF_019443785.1	GCF_003030945.1	10940	4639.24761	P. mirabilis
GCF_000391485.2	GCF_003966385.1	10951	6836.43192	E. faecalis
GCF_001989555.1	GCF_018517125.1	10974	6850.79024	E. faecalis
GCF_018517125.1	GCF_003966385.1	10988	6859.53008	E. faecalis
GCF_018986755.2	GCF_003966385.1	11034	6888.24672	E. faecalis
GCF_018336495.1	GCF_003073935.1	11051	4686.31859	P. mirabilis
GCF_003666405.1	GCF_002125925.1	11055	10902.3776	E. coli
GCF_003072445.1	GCF_002125925.1	11062	10909.281	E. coli
GCF_016864435.1	GCF_004358945.1	11066	10913.2258	E. coli
GCF_000391485.2	GCF_018517125.1	11067	6908.84778	E. faecalis
GCF_016864435.1	GCF_002879735.1	11083	10929.9911	E. coli
GCF_011383025.1	GCF_002055685.1	11091	4703.2811	P. mirabilis
GCF_014843115.1	GCF_014931585.1	11095	4704.97735	P. mirabilis
GCF_011149675.1	GCF_008041895.1	11102	4707.94579	P. mirabilis
GCF_003017885.1	GCF_002125925.1	11119	10965.494	E. coli
GCF_014216355.1	GCF_002125925.1	11121	10967.4664	E. coli
GCF_002736085.1	GCF_002125925.1	11132	10978.3146	E. coli
GCF_011149675.1	GCF_000783575.2	11136	4722.36393	P. mirabilis
GCF_018986755.2	GCF_018517125.1	11139	6953.79556	E. faecalis
GCF_014843115.1	GCF_000069965.1	11150	4728.30081	P. mirabilis
GCF_002310875.1	GCF_008630655.1	11155	4730.42112	P. mirabilis
GCF_003367575.1	GCF_002125925.1	11156	11001.9832	E. coli
GCF_018336495.1	GCF_018138945.1	11161	4732.9655	P. mirabilis
GCF_014843115.1	GCF_011383025.1	11180	4741.0227	P. mirabilis
GCF_000391485.2	GCF_009662495.1	11181	6980.01509	E. faecalis
GCF_013343255.1	GCF_008630655.1	11185	4743.14301	P. mirabilis
GCF_003815075.1	GCF_019284495.1	11195	3734.31056	K. pneumoniae
GCF_006514375.1	GCF_004358945.1	11196	11041.431	E. coli
GCF_018972025.1	GCF_002055685.1	11202	4750.35208	P. mirabilis
GCF_006514375.1	GCF_002879735.1	11216	11061.1549	E. coli
GCF_002310875.1	GCF_002055685.1	11222	4758.83334	P. mirabilis
GCF_014931585.1	GCF_011045575.1	11227	4760.95365	P. mirabilis
GCF_003030945.1	GCF_003073935.1	11244	4768.16272	P. mirabilis
GCF_016864435.1	GCF_003666405.1	11249	11093.6993	E. coli
GCF_013343255.1	GCF_002055685.1	11253	4771.97928	P. mirabilis
GCF_001640985.1	GCF_008630655.1	11265	4777.06804	P. mirabilis
GCF_011149675.1	GCF_019192645.1	11267	4777.91616	P. mirabilis
GCF_013343255.1	GCF_014843115.1	11278	4782.58086	P. mirabilis
GCF_016864435.1	GCF_003112045.1	11280	11124.2713	E. coli
GCF_000391485.2	GCF_002208945.1	11285	7044.93966	E. faecalis
GCF_001640985.1	GCF_002055685.1	11287	4786.39742	P. mirabilis
GCF_002310875.1	GCF_014843115.1	11294	4789.36586	P. mirabilis
GCF_016864435.1	GCF_018208275.1	11297	11141.0366	E. coli
GCF_002208945.1	GCF_001989555.1	11308	7059.29798	E. faecalis
GCF_018972025.1	GCF_014931585.1	11313	4797.42305	P. mirabilis
GCF_008041895.1	GCF_000783575.2	11322	4801.23962	P. mirabilis
GCF_007632055.1	GCF_018517125.1	11323	7068.6621	E. faecalis
GCF_018972025.1	GCF_011383025.1	11366	4819.89839	P. mirabilis
GCF_002208945.1	GCF_003966385.1	11380	7104.24575	E. faecalis
GCF_006514375.1	GCF_003666405.1	11381	11223.8769	E. coli
GCF_016864435.1	GCF_002634895.1	11381	11223.8769	E. coli
GCF_016864435.1	GCF_000166535.2	11392	11234.7251	E. coli
GCF_018972025.1	GCF_000069965.1	11396	4832.62027	P. mirabilis
GCF_016801475.1	GCF_002125925.1	11403	11245.5732	E. coli

GCF_013343255.1	GCF_018972025.1	11409	4838.13309	P. mirabilis
GCF_006514375.1	GCF_003112045.1	11412	11254.449	E. coli
GCF_002310875.1	GCF_018972025.1	11425	4844.91809	P. mirabilis
GCF_007632055.1	GCF_009662495.1	11427	7133.58666	E. faecalis
GCF_006514375.1	GCF_018208275.1	11431	11273.1867	E. coli
GCF_000240185.1	GCF_019284495.1	11451	3819.70435	K. pneumoniae
GCF_016864435.1	GCF_003017885.1	11452	11293.8967	E. coli
GCF_016864435.1	GCF_002736085.1	11454	11295.8691	E. coli
GCF_016864435.1	GCF_003072445.1	11461	11302.7725	E. coli
GCF_001640985.1	GCF_018972025.1	11475	4866.12124	P. mirabilis
GCF_009684615.1	GCF_019284495.1	11486	3831.37928	K. pneumoniae
GCF_003073935.1	GCF_000783575.2	11490	4872.48218	P. mirabilis
GCF_002208945.1	GCF_018986755.2	11491	7173.54024	E. faecalis
GCF_013407495.1	GCF_019284495.1	11492	3833.3807	K. pneumoniae
GCF_016864435.1	GCF_003367575.1	11497	11338.2755	E. coli
GCF_019192645.1	GCF_000783575.2	11512	4881.81156	P. mirabilis
GCF_006514375.1	GCF_002634895.1	11513	11354.0546	E. coli
GCF_016864435.1	GCF_014216355.1	11522	11362.9303	E. coli
GCF_006514375.1	GCF_000166535.2	11522	11362.9303	E. coli
GCF_001640985.1	GCF_014843115.1	11527	4888.17251	P. mirabilis
GCF_007632055.1	GCF_002208945.1	11540	7204.1297	E. faecalis
GCF_018336495.1	GCF_003030945.1	11541	4894.10939	P. mirabilis
GCF_013407495.1	GCF_000240185.1	11554	3854.06201	K. pneumoniae
GCF_002211665.1	GCF_019284495.1	11554	3854.06201	K. pneumoniae
GCF_002945235.1	GCF_019443785.1	11578	4909.79971	P. mirabilis
GCF_019443785.1	GCF_011149675.1	11578	4909.79971	P. mirabilis
GCF_013343255.1	GCF_019443785.1	11581	4911.0719	P. mirabilis
GCF_006514375.1	GCF_003017885.1	11582	11422.102	E. coli
GCF_006514375.1	GCF_002736085.1	11584	11424.0744	E. coli
GCF_018336495.1	GCF_000783575.2	11585	4912.76815	P. mirabilis
GCF_006514375.1	GCF_003072445.1	11593	11432.9501	E. coli
GCF_000069965.1	GCF_011045575.1	11593	4916.16065	P. mirabilis
GCF_002310875.1	GCF_019443785.1	11598	4918.28097	P. mirabilis
GCF_019192645.1	GCF_008041895.1	11603	4920.40128	P. mirabilis
GCF_013407495.1	GCF_009684615.1	11621	3876.41117	K. pneumoniae
GCF_006514375.1	GCF_003367575.1	11627	11466.4807	E. coli
GCF_006514375.1	GCF_014216355.1	11652	11491.1356	E. coli
GCF_016864435.1	GCF_019614135.1	11654	11493.108	E. coli
GCF_018138945.1	GCF_019192645.1	11661	4944.99693	P. mirabilis
GCF_003073935.1	GCF_008041895.1	11673	4950.08568	P. mirabilis
GCF_013407495.1	GCF_002211665.1	11681	3896.42534	K. pneumoniae
GCF_018336495.1	GCF_008041895.1	11699	4961.11132	P. mirabilis
GCF_016864435.1	GCF_016801475.1	11743	11580.8793	E. coli
GCF_013343255.1	GCF_011045575.1	11769	4990.79572	P. mirabilis
GCF_003073935.1	GCF_011149675.1	11776	4993.76416	P. mirabilis
GCF_002310875.1	GCF_011045575.1	11785	4997.58072	P. mirabilis
GCF_006514375.1	GCF_019614135.1	11786	11623.2856	E. coli
GCF_019443785.1	GCF_008041895.1	11788	4998.85291	P. mirabilis
GCF_900069965.1_KPN_RH201207_genomic	GCF_019284495.1	11793	3933.78512	K. pneumoniae
GCF_019443785.1	GCF_000783575.2	11806	5006.48604	P. mirabilis
GCF_002055685.1	GCF_011045575.1	11813	5009.45448	P. mirabilis
GCF_001640985.1	GCF_011149675.1	11836	5019.20793	P. mirabilis
GCF_001006265.1	GCF_013407495.1	11841	3949.79646	K. pneumoniae
GCF_011383025.1	GCF_011045575.1	11841	5021.32824	P. mirabilis
GCF_001640985.1	GCF_011045575.1	11859	5028.96137	P. mirabilis
GCF_008630655.1	GCF_011045575.1	11862	5030.23356	P. mirabilis
GCF_006514375.1	GCF_016801475.1	11873	11709.0845	E. coli
GCF_013407495.1	GCF_900069965.1_KPN_RH201207_genomic	11875	3961.13782	K. pneumoniae
GCF_002945235.1	GCF_018138945.1	11893	5043.37951	P. mirabilis
GCF_013343255.1	GCF_011149675.1	11914	5052.28483	P. mirabilis
GCF_018336495.1	GCF_011149675.1	11924	5056.52546	P. mirabilis
GCF_001006265.1	GCF_019284495.1	11931	3979.81771	K. pneumoniae
GCF_002310875.1	GCF_011149675.1	11931	5059.4939	P. mirabilis
GCF_011149675.1	GCF_011045575.1	11933	5060.34202	P. mirabilis
GCF_002945235.1	GCF_003073935.1	11941	5063.73453	P. mirabilis
GCF_018336495.1	GCF_019192645.1	11942	5064.15859	P. mirabilis
GCF_003073935.1	GCF_019192645.1	11963	5073.06391	P. mirabilis
GCF_000783575.2	GCF_011045575.1	11969	5075.60829	P. mirabilis
GCF_018138945.1	GCF_011149675.1	11973	5077.30454	P. mirabilis
GCF_003030945.1	GCF_000783575.2	11978	5079.42485	P. mirabilis
GCF_003030945.1	GCF_011149675.1	11981	5080.69704	P. mirabilis
GCF_018138945.1	GCF_000783575.2	11985	5082.39329	P. mirabilis
GCF_013407495.1	GCF_003815075.1	12003	4003.83471	K. pneumoniae
GCF_010103655.1	GCF_009662495.1	12010	7497.5388	E. faecalis
GCF_008630655.1	GCF_008041895.1	12018	5096.38737	P. mirabilis
GCF_012594215.1	GCF_009662495.1	12023	7505.65437	E. faecalis
GCF_006349345.1	GCF_009662495.1	12024	7506.27864	E. faecalis
GCF_002945235.1	GCF_000783575.2	12041	5106.14081	P. mirabilis
GCF_008630655.1	GCF_000783575.2	12041	5106.14081	P. mirabilis
GCF_003030945.1	GCF_019192645.1	12055	5112.07769	P. mirabilis
GCF_010103655.1	GCF_017639585.1	12099	7553.09924	E. faecalis
GCF_006349345.1	GCF_017639585.1	12109	7559.34199	E. faecalis
GCF_001006265.1	GCF_000240185.1	12109	4039.19308	K. pneumoniae
GCF_012594215.1	GCF_017639585.1	12112	7561.21481	E. faecalis
GCF_017639585.1	GCF_000550745.1	12112	7561.21481	E. faecalis
GCF_001006265.1	GCF_002211665.1	12117	4041.86164	K. pneumoniae
GCF_019443785.1	GCF_019192645.1	12133	5145.15459	P. mirabilis
GCF_001006265.1	GCF_009684615.1	12137	4048.53303	K. pneumoniae
GCF_001006265.1	GCF_003815075.1	12143	4050.53445	K. pneumoniae
GCF_009662495.1	GCF_000550745.1	12147	7583.06443	E. faecalis
GCF_002055685.1	GCF_008041895.1	12151	5152.78773	P. mirabilis
GCF_008041895.1	GCF_011045575.1	12151	5152.78773	P. mirabilis
GCF_014931585.1	GCF_019192645.1	12153	5153.63585	P. mirabilis
GCF_017639585.1	GCF_014893535.1	12167	7595.54992	E. faecalis

GCF_018138945.1	GCF_008041895.1	12167	5159.57273	P. mirabilis
GCF_002945235.1	GCF_019192645.1	12171	5161.26898	P. mirabilis
GCF_000069965.1	GCF_011149675.1	12174	5162.54117	P. mirabilis
GCF_000391485.2	GCF_017639585.1	12181	7604.28977	E. faecalis
GCF_002055685.1	GCF_000783575.2	12198	5172.71868	P. mirabilis
GCF_009662495.1	GCF_018986755.2	12199	7615.52671	E. faecalis
GCF_000069965.1	GCF_000783575.2	12214	5179.50369	P. mirabilis
GCF_002055685.1	GCF_019443785.1	12215	5179.92775	P. mirabilis
GCF_002055685.1	GCF_019192645.1	12216	5180.35181	P. mirabilis
GCF_014843115.1	GCF_011045575.1	12220	5182.04806	P. mirabilis
GCF_014931585.1	GCF_011149675.1	12225	5184.16838	P. mirabilis
GCF_009662495.1	GCF_014893535.1	12229	7634.25495	E. faecalis
GCF_008630655.1	GCF_011149675.1	12230	5186.28869	P. mirabilis
GCF_002310875.1	GCF_000783575.2	12234	5187.98494	P. mirabilis
GCF_011383025.1	GCF_011149675.1	12237	5189.25713	P. mirabilis
GCF_016859025.1	GCF_013407495.1	12247	4085.22567	K. pneumoniae
GCF_008630655.1	GCF_019192645.1	12252	5195.61807	P. mirabilis
GCF_002055685.1	GCF_011149675.1	12260	5199.01058	P. mirabilis
GCF_013343255.1	GCF_000783575.2	12265	5201.13089	P. mirabilis
GCF_014931585.1	GCF_019443785.1	12272	5204.09933	P. mirabilis
GCF_009662495.1	GCF_001989555.1	12282	7667.34151	E. faecalis
GCF_003030945.1	GCF_008041895.1	12282	5208.33996	P. mirabilis
GCF_017639585.1	GCF_018517125.1	12283	7667.96578	E. faecalis
GCF_013343255.1	GCF_008041895.1	12284	5209.18809	P. mirabilis
GCF_000069965.1	GCF_008041895.1	12286	5210.03621	P. mirabilis
GCF_016859025.1	GCF_019284495.1	12296	4101.57058	K. pneumoniae
GCF_009662495.1	GCF_018517125.1	12298	7677.3299	E. faecalis
GCF_002310875.1	GCF_008041895.1	12301	5216.39715	P. mirabilis
GCF_011383025.1	GCF_019192645.1	12307	5218.94153	P. mirabilis
GCF_001640985.1	GCF_000783575.2	12314	5221.90997	P. mirabilis
GCF_019192645.1	GCF_011045575.1	12324	5226.1506	P. mirabilis
GCF_002945235.1	GCF_003030945.1	12329	5228.27091	P. mirabilis
GCF_001640985.1	GCF_008041895.1	12336	5231.23935	P. mirabilis
GCF_001006265.1	GCF_900069965.1_KPN_RH201207_genomic	12344	4117.58191	K. pneumoniae
GCF_014931585.1	GCF_000783575.2	12353	5238.44842	P. mirabilis
GCF_002945235.1	GCF_011149675.1	12357	5240.14467	P. mirabilis
GCF_018972025.1	GCF_011045575.1	12364	5243.11311	P. mirabilis
GCF_003073935.1	GCF_011045575.1	12394	5255.835	P. mirabilis
GCF_014931585.1	GCF_008041895.1	12395	5256.25906	P. mirabilis
GCF_009662495.1	GCF_902164645.1_25426_7_121_genomic	12404	7743.50302	E. faecalis
GCF_017639585.1	GCF_001989555.1	12417	7751.61859	E. faecalis
GCF_007632055.1	GCF_017639585.1	12418	7752.24286	E. faecalis
GCF_002310875.1	GCF_019192645.1	12422	5267.70876	P. mirabilis
GCF_017639585.1	GCF_018986755.2	12427	7757.86133	E. faecalis
GCF_000069965.1	GCF_019443785.1	12429	5270.6772	P. mirabilis
GCF_017639585.1	GCF_902164645.1_25426_7_121_genomic	12434	7762.23126	E. faecalis
GCF_016859025.1	GCF_001006265.1	12439	4149.27102	K. pneumoniae
GCF_002945235.1	GCF_008041895.1	12442	5276.19002	P. mirabilis
GCF_013706045.1	GCF_019711255.1	12445	4151.27243	K. pneumoniae
GCF_008630655.1	GCF_018138945.1	12447	5278.31033	P. mirabilis
GCF_017639585.1	GCF_009662495.1	12448	7770.9711	E. faecalis
GCF_013343255.1	GCF_019192645.1	12453	5280.85471	P. mirabilis
GCF_008630655.1	GCF_003073935.1	12473	5289.33596	P. mirabilis
GCF_019443785.1	GCF_011045575.1	12485	5294.42472	P. mirabilis
GCF_018138945.1	GCF_011045575.1	12486	5294.84878	P. mirabilis
GCF_000069965.1	GCF_003073935.1	12493	5297.81722	P. mirabilis
GCF_002055685.1	GCF_003073935.1	12496	5299.08941	P. mirabilis
GCF_018336495.1	GCF_008630655.1	12507	5303.7541	P. mirabilis
GCF_011383025.1	GCF_008041895.1	12515	5307.1466	P. mirabilis
GCF_016859025.1	GCF_002211665.1	12518	4175.62301	K. pneumoniae
GCF_016859025.1	GCF_000240185.1	12524	4177.62442	K. pneumoniae
GCF_002310875.1	GCF_018138945.1	12525	5311.38723	P. mirabilis
GCF_013343255.1	GCF_003073935.1	12543	5319.02036	P. mirabilis
GCF_016859025.1	GCF_003815075.1	12545	4184.62938	K. pneumoniae
GCF_018336495.1	GCF_014931585.1	12548	5321.14068	P. mirabilis
GCF_000069965.1	GCF_019192645.1	12551	5322.41287	P. mirabilis
GCF_013343255.1	GCF_018138945.1	12555	5324.10912	P. mirabilis
GCF_002055685.1	GCF_018138945.1	12555	5324.10912	P. mirabilis
GCF_011383025.1	GCF_003073935.1	12557	5324.95724	P. mirabilis
GCF_014931585.1	GCF_018138945.1	12559	5325.80537	P. mirabilis
GCF_001640985.1	GCF_018138945.1	12560	5326.22943	P. mirabilis
GCF_002310875.1	GCF_003073935.1	12560	5326.22943	P. mirabilis
GCF_009662495.1	GCF_003966385.1	12563	7842.76269	E. faecalis
GCF_011383025.1	GCF_000783575.2	12569	5330.046	P. mirabilis
GCF_001640985.1	GCF_019192645.1	12570	5330.47006	P. mirabilis
GCF_014931585.1	GCF_003073935.1	12579	5334.28663	P. mirabilis
GCF_008630655.1	GCF_019443785.1	12585	5336.831	P. mirabilis
GCF_011383025.1	GCF_019443785.1	12591	5339.37538	P. mirabilis
GCF_017639585.1	GCF_003966385.1	12594	7862.1152	E. faecalis
GCF_011383025.1	GCF_018138945.1	12607	5346.16039	P. mirabilis
GCF_018336495.1	GCF_011045575.1	12620	5351.6732	P. mirabilis
GCF_003963495.1	GCF_019284495.1	12624	4210.98137	K. pneumoniae
GCF_003963495.1	GCF_013407495.1	12626	4211.64851	K. pneumoniae
GCF_008630655.1	GCF_003030945.1	12631	5356.33789	P. mirabilis
GCF_018336495.1	GCF_002055685.1	12633	5357.18602	P. mirabilis
GCF_018336495.1	GCF_000069965.1	12634	5357.61008	P. mirabilis
GCF_018972025.1	GCF_011149675.1	12647	5363.1229	P. mirabilis
GCF_002208945.1	GCF_009662495.1	12649	7896.45031	E. faecalis
GCF_016859025.1	GCF_009684615.1	12653	4220.65489	K. pneumoniae
GCF_002208945.1	GCF_017639585.1	12657	7901.44451	E. faecalis
GCF_014843115.1	GCF_008041895.1	12676	5375.42072	P. mirabilis
GCF_000069965.1	GCF_018138945.1	12701	5386.02229	P. mirabilis
GCF_001640985.1	GCF_003073935.1	12702	5386.44636	P. mirabilis
GCF_014843115.1	GCF_011149675.1	12710	5389.83886	P. mirabilis

GCF_014843115.1	GCF_019192645.1	12712	5390.68699	P. mirabilis
GCF_014843115.1	GCF_000783575.2	12719	5393.65543	P. mirabilis
GCF_014843115.1	GCF_003073935.1	12748	5405.95325	P. mirabilis
GCF_003963495.1	GCF_001006265.1	12787	4265.3532	K. pneumoniae
GCF_013343255.1	GCF_018336495.1	12820	5436.48577	P. mirabilis
GCF_002310875.1	GCF_018336495.1	12836	5443.27078	P. mirabilis
GCF_003030945.1	GCF_011045575.1	12842	5445.81516	P. mirabilis
GCF_016859025.1	GCF_900069965.1_KPN_RH201207_genomic	12849	4286.03451	K. pneumoniae
GCF_003963495.1	GCF_002211665.1	12855	4288.03593	K. pneumoniae
GCF_018336495.1	GCF_011383025.1	12855	5451.32797	P. mirabilis
GCF_003963495.1	GCF_016859025.1	12874	4294.37375	K. pneumoniae
GCF_003963495.1	GCF_000240185.1	12877	4295.37446	K. pneumoniae
GCF_018972025.1	GCF_008041895.1	12888	5465.32205	P. mirabilis
GCF_003963495.1	GCF_009684615.1	12914	4307.71653	K. pneumoniae
GCF_002055685.1	GCF_003030945.1	12918	5478.04393	P. mirabilis
GCF_018972025.1	GCF_019192645.1	12929	5482.70863	P. mirabilis
GCF_001640985.1	GCF_018336495.1	12938	5486.52519	P. mirabilis
GCF_018972025.1	GCF_000783575.2	12945	5489.49363	P. mirabilis
GCF_014843115.1	GCF_019443785.1	12957	5494.58239	P. mirabilis
GCF_016864435.1	GCF_002125925.1	12960	12781.0777	E. coli
GCF_002945235.1	GCF_002055685.1	12963	5497.12676	P. mirabilis
GCF_003963495.1	GCF_003815075.1	12974	4327.7307	K. pneumoniae
GCF_000069965.1	GCF_003030945.1	12984	5506.03208	P. mirabilis
GCF_002310875.1	GCF_003030945.1	12986	5506.88021	P. mirabilis
GCF_018972025.1	GCF_019443785.1	12992	5509.42458	P. mirabilis
GCF_011383025.1	GCF_003030945.1	13012	5517.90584	P. mirabilis
GCF_013343255.1	GCF_003030945.1	13015	5519.17803	P. mirabilis
GCF_000967845.1	GCF_019711255.1	13021	4343.40847	K. pneumoniae
GCF_002945235.1	GCF_008630655.1	13081	5547.16618	P. mirabilis
GCF_018972025.1	GCF_003073935.1	13087	5549.71056	P. mirabilis
GCF_006514375.1	GCF_002125925.1	13092	12911.2553	E. coli
GCF_003963495.1	GCF_900069965.1_KPN_RH201207_genomic	13096	4368.42618	K. pneumoniae
GCF_002125925.1	GCF_014898635.1	13097	12916.1863	E. coli
GCF_002125925.1	GCF_001938625.2	13107	12926.0483	E. coli
GCF_002945235.1	GCF_000069965.1	13131	5568.36932	P. mirabilis
GCF_014931585.1	GCF_003030945.1	13142	5573.03401	P. mirabilis
GCF_014843115.1	GCF_018138945.1	13160	5580.66714	P. mirabilis
GCF_001640985.1	GCF_003030945.1	13161	5581.09121	P. mirabilis
GCF_014843115.1	GCF_003030945.1	13187	5592.11684	P. mirabilis
GCF_018336495.1	GCF_014843115.1	13189	5592.96497	P. mirabilis
GCF_002945235.1	GCF_014931585.1	13212	5602.71841	P. mirabilis
GCF_002945235.1	GCF_011045575.1	13256	5621.37718	P. mirabilis
GCF_002945235.1	GCF_011383025.1	13324	5650.21345	P. mirabilis
GCF_018336495.1	GCF_018972025.1	13377	5672.68878	P. mirabilis
GCF_018972025.1	GCF_018138945.1	13386	5676.50535	P. mirabilis
GCF_002310875.1	GCF_002945235.1	13393	5679.47379	P. mirabilis
GCF_001640985.1	GCF_002945235.1	13399	5682.01817	P. mirabilis
GCF_013343255.1	GCF_002945235.1	13423	5692.19567	P. mirabilis
GCF_002125925.1	GCF_007107705.1	13441	13255.4371	E. coli
GCF_018972025.1	GCF_003030945.1	13458	5707.03787	P. mirabilis
GCF_004138625.1	GCF_001938625.2	13718	13528.6129	E. coli
GCF_003194125.1	GCF_014898635.1	13739	13549.323	E. coli
GCF_002945235.1	GCF_014843115.1	13785	5845.70643	P. mirabilis
GCF_004138625.1	GCF_007107705.1	13792	13601.5913	E. coli
GCF_002945235.1	GCF_018972025.1	13852	5874.11864	P. mirabilis
GCF_003194125.1	GCF_007107705.1	13859	13667.6663	E. coli
GCF_004138625.1	GCF_014898635.1	13970	13777.1339	E. coli
GCF_017900915.1	GCF_001045685.1	14050	9981.11048	P. aeruginosa
GCF_008195605.1	GCF_002945235.1	14070	5966.56434	P. mirabilis
GCF_003194125.1	GCF_001938625.2	14092	13897.4496	E. coli
GCF_017900915.1	GCF_900149285.1_PcyII-10_genomic	14099	10015.92	P. aeruginosa
GCF_017900915.1	GCF_002968515.1	14145	10048.5984	P. aeruginosa
GCF_017900915.1	GCF_003204335.1	14145	10048.5984	P. aeruginosa
GCF_017900915.1	GCF_016126955.1	14147	10050.0192	P. aeruginosa
GCF_017900915.1	GCF_000226155.1	14162	10060.6752	P. aeruginosa
GCF_017900915.1	GCF_000026645.1	14169	10065.648	P. aeruginosa
GCF_017900915.1	GCF_019466145.1	14201	10088.3808	P. aeruginosa
GCF_017900915.1	GCF_000271985.2	14215	10098.3264	P. aeruginosa
GCF_017900915.1	GCF_012935295.1	14222	10103.2992	P. aeruginosa
GCF_017900915.1	GCF_900070375.1_PAO1OR_genomic	14224	10104.72	P. aeruginosa
GCF_017900915.1	GCF_013305765.1	14224	10104.72	P. aeruginosa
GCF_017900915.1	GCF_004014755.1	14227	10106.8512	P. aeruginosa
GCF_017900915.1	GCF_001293085.1	14227	10106.8512	P. aeruginosa
GCF_017900915.1	GCF_000496605.2	14227	10106.8512	P. aeruginosa
GCF_017900915.1	GCF_009648875.1	14231	10109.6928	P. aeruginosa
GCF_017900915.1	GCF_004102665.1	14234	10111.824	P. aeruginosa
GCF_017900915.1	GCF_014854655.1	14235	10112.5344	P. aeruginosa
GCF_017900915.1	GCF_000414035.1	14235	10112.5344	P. aeruginosa
GCF_017900915.1	GCF_003571505.1	14240	10116.0864	P. aeruginosa
GCF_017900915.1	GCF_003319235.1	14242	10117.5072	P. aeruginosa
GCF_017900915.1	GCF_001750705.1	14245	10119.6383	P. aeruginosa
GCF_017900915.1	GCF_018409365.1	14249	10122.4799	P. aeruginosa
GCF_017900915.1	GCF_000524595.1	14253	10125.3215	P. aeruginosa
GCF_017900915.1	GCF_014792125.1	14254	10126.0319	P. aeruginosa
GCF_017900915.1	GCF_001879525.1	14263	10132.4255	P. aeruginosa
GCF_017900915.1	GCF_008033725.1	14267	10135.2671	P. aeruginosa
GCF_017900915.1	GCF_904866275.1_MINF_7A_genomic	14286	10148.7647	P. aeruginosa
GCF_017900915.1	GCF_001606045.1	14296	10155.8687	P. aeruginosa
GCF_009429045.2	GCF_002945235.1	14301	6064.52286	P. mirabilis
GCF_010442675.1	GCF_002945235.1	14316	6070.8838	P. mirabilis
GCF_017900915.1	GCF_001900195.1	14327	10177.8911	P. aeruginosa
GCF_017900915.1	GCF_009911735.1	14327	10177.8911	P. aeruginosa
GCF_017900915.1	GCF_900243355.1_RW109_genomic	14328	10178.6015	P. aeruginosa
GCF_017900915.1	GCF_016105505.1	14332	10181.4431	P. aeruginosa

GCF_017900915.1	GCF_000568855.2	14333	10182.1535	P. aeruginosa
GCF_000444425.1	GCF_002945235.1	14342	6081.90944	P. mirabilis
GCF_017900915.1	GCF_900636735.1_43941_C01_genomic	14416	10241.1166	P. aeruginosa
GCF_002634895.1	GCF_014898635.1	14420	14220.9213	E. coli
GCF_017900915.1	GCF_900497025.1	14814	10523.8556	P. aeruginosa
GCF_017900915.1	GCF_000981825.1	14815	10524.566	P. aeruginosa
GCF_013706045.1	GCF_019284495.1	14947	4985.86332	K. pneumoniae
GCF_003112045.1	GCF_001938625.2	14962	14755.4386	E. coli
GCF_003666405.1	GCF_001938625.2	14998	14790.9416	E. coli
GCF_017900915.1	GCF_001874465.1	15005	10659.5418	P. aeruginosa
GCF_002736085.1	GCF_001938625.2	15085	14876.7405	E. coli
GCF_002736085.1	GCF_014898635.1	15112	14903.3678	E. coli
GCF_003666405.1	GCF_014898635.1	15119	14910.2711	E. coli
GCF_014216355.1	GCF_001938625.2	15128	14919.1469	E. coli
GCF_017900915.1	GCF_001722005.2	15144	10758.2873	P. aeruginosa
GCF_003367575.1	GCF_001938625.2	15147	14937.8846	E. coli
GCF_010442675.1	GCF_018138945.1	15147	6423.28003	P. mirabilis
GCF_017900915.1	GCF_000284555.1	15166	10773.9161	P. aeruginosa
GCF_003112045.1	GCF_014898635.1	15168	14958.5946	E. coli
GCF_013706045.1	GCF_013407495.1	15168	5059.58218	K. pneumoniae
GCF_009429045.2	GCF_018138945.1	15171	6433.45754	P. mirabilis
GCF_008195605.1	GCF_018138945.1	15172	6433.8816	P. mirabilis
GCF_003666405.1	GCF_007107705.1	15173	14963.5256	E. coli
GCF_000444425.1	GCF_018138945.1	15174	6434.72973	P. mirabilis
GCF_000166535.2	GCF_001938625.2	15182	14972.4014	E. coli
GCF_013706045.1	GCF_001006265.1	15182	5064.25216	K. pneumoniae
GCF_017900915.1	GCF_002287725.2	15205	10801.6217	P. aeruginosa
GCF_003112045.1	GCF_007107705.1	15210	15000.0148	E. coli
GCF_002634895.1	GCF_007107705.1	15211	15001.001	E. coli
GCF_008195605.1	GCF_011149675.1	15223	6455.50881	P. mirabilis
GCF_002634895.1	GCF_001938625.2	15228	15017.7663	E. coli
GCF_017900915.1	GCF_900095805.1_PA14Or_genomic	15239	10825.7753	P. aeruginosa
GCF_003072445.1	GCF_001938625.2	15240	15029.6006	E. coli
GCF_004358945.1	GCF_014898635.1	15242	15031.573	E. coli
GCF_003072445.1	GCF_014898635.1	15244	15033.5454	E. coli
GCF_017900915.1	GCF_000014625.1	15250	10833.5897	P. aeruginosa
GCF_010442675.1	GCF_011149675.1	15253	6468.2307	P. mirabilis
GCF_004358945.1	GCF_001938625.2	15263	15052.2831	E. coli
GCF_017900915.1	GCF_002223805.1	15264	10843.5353	P. aeruginosa
GCF_000166535.2	GCF_014898635.1	15283	15072.007	E. coli
GCF_002879735.1	GCF_014898635.1	15287	15075.9518	E. coli
GCF_002879735.1	GCF_001938625.2	15293	15081.8689	E. coli
GCF_003017885.1	GCF_001938625.2	15295	15083.8413	E. coli
GCF_019614135.1	GCF_001938625.2	15295	15083.8413	E. coli
GCF_016801475.1	GCF_001938625.2	15302	15090.7447	E. coli
GCF_002736085.1	GCF_007107705.1	15303	15091.7309	E. coli
GCF_008195605.1	GCF_019192645.1	15307	6491.13009	P. mirabilis
GCF_003367575.1	GCF_014898635.1	15311	15099.6204	E. coli
GCF_000166535.2	GCF_007107705.1	15327	15115.3995	E. coli
GCF_014216355.1	GCF_014898635.1	15332	15120.3305	E. coli
GCF_009429045.2	GCF_011149675.1	15342	6505.97229	P. mirabilis
GCF_010442675.1	GCF_003073935.1	15343	6506.39635	P. mirabilis
GCF_000444425.1	GCF_019192645.1	15349	6508.94073	P. mirabilis
GCF_000444425.1	GCF_011149675.1	15355	6511.48511	P. mirabilis
GCF_003017885.1	GCF_014898635.1	15363	15150.9025	E. coli
GCF_013706045.1	GCF_003815075.1	15365	5125.29538	K. pneumoniae
GCF_010442675.1	GCF_019192645.1	15373	6519.11824	P. mirabilis
GCF_013706045.1	GCF_000240185.1	15377	5129.29821	K. pneumoniae
GCF_019614135.1	GCF_014898635.1	15379	15166.6816	E. coli
GCF_003072445.1	GCF_007107705.1	15381	15168.654	E. coli
GCF_010442675.1	GCF_019443785.1	15386	6524.63105	P. mirabilis
GCF_009429045.2	GCF_018336495.1	15391	6526.75137	P. mirabilis
GCF_016801475.1	GCF_014898635.1	15400	15187.3917	E. coli
GCF_018208275.1	GCF_001938625.2	15410	15197.2536	E. coli
GCF_009429045.2	GCF_019192645.1	15418	6538.20107	P. mirabilis
GCF_009429045.2	GCF_019443785.1	15427	6542.01763	P. mirabilis
GCF_014216355.1	GCF_007107705.1	15431	15217.9637	E. coli
GCF_003367575.1	GCF_007107705.1	15438	15224.8671	E. coli
GCF_004358945.1	GCF_007107705.1	15445	15231.7704	E. coli
GCF_012955465.1	GCF_003194125.1	15452	15238.6738	E. coli
GCF_008195605.1	GCF_019443785.1	15454	6553.46733	P. mirabilis
GCF_013706045.1	GCF_002211665.1	15461	5157.31805	K. pneumoniae
GCF_013706045.1	GCF_009684615.1	15462	5157.65162	K. pneumoniae
GCF_008195605.1	GCF_018336495.1	15463	6557.28389	P. mirabilis
GCF_002125925.1	GCF_004138625.1	15464	15250.5081	E. coli
GCF_008195605.1	GCF_003073935.1	15477	6563.22077	P. mirabilis
GCF_000444425.1	GCF_003073935.1	15482	6565.34109	P. mirabilis
GCF_002879735.1	GCF_007107705.1	15496	15282.0664	E. coli
GCF_003017885.1	GCF_007107705.1	15497	15283.0525	E. coli
GCF_902164685.1_25426_7_120_genomic	GCF_003194125.1	15503	15288.9697	E. coli
GCF_018986775.1	GCF_003194125.1	15509	15294.8869	E. coli
GCF_016801475.1	GCF_007107705.1	15511	15296.8593	E. coli
GCF_000444425.1	GCF_019443785.1	15511	6577.63891	P. mirabilis
GCF_018208275.1	GCF_014898635.1	15517	15302.7764	E. coli
GCF_018208275.1	GCF_007107705.1	15526	15311.6522	E. coli
GCF_010442675.1	GCF_018336495.1	15530	6585.69611	P. mirabilis
GCF_012955465.1	GCF_004138625.1	15546	15331.3761	E. coli
GCF_009429045.2	GCF_000783575.2	15546	6592.48111	P. mirabilis
GCF_008195605.1	GCF_000783575.2	15555	6596.29768	P. mirabilis
GCF_002125925.1	GCF_003194125.1	15557	15342.2242	E. coli
GCF_000444425.1	GCF_000783575.2	15559	6597.99393	P. mirabilis
GCF_009429045.2	GCF_003073935.1	15569	6602.23456	P. mirabilis
GCF_010442675.1	GCF_000783575.2	15587	6609.86769	P. mirabilis
GCF_016864435.1	GCF_001938625.2	15617	15401.3959	E. coli

GCF_019614135.1	GCF_007107705.1	15626	15410.2716	E. coli
GCF_000967845.1	GCF_013407495.1	15630	5213.69129	K. pneumoniae
GCF_013706045.1	GCF_900069965.1_KPN_RH201207_genomic	15636	5215.69271	K. pneumoniae
GCF_003999775.1	GCF_004138625.1	15639	15423.0921	E. coli
GCF_000444425.1	GCF_018336495.1	15640	6632.34302	P. mirabilis
GCF_003999775.1	GCF_003194125.1	15657	15440.8436	E. coli
GCF_003028695.1	GCF_004138625.1	15660	15443.8022	E. coli
GCF_013706045.1	GCF_016859025.1	15667	5226.03336	K. pneumoniae
GCF_013344585.1	GCF_004138625.1	15675	15458.5951	E. coli
GCF_018986775.1	GCF_004138625.1	15677	15460.5675	E. coli
GCF_003028695.1	GCF_003194125.1	15680	15463.5261	E. coli
GCF_013344585.1	GCF_003194125.1	15695	15478.319	E. coli
GCF_902164685.1_25426_7_120_genomic	GCF_004138625.1	15696	15479.3052	E. coli
GCF_006514375.1	GCF_001938625.2	15739	15521.7116	E. coli
GCF_000444425.1	GCF_011383025.1	15743	6676.02149	P. mirabilis
GCF_008195605.1	GCF_011383025.1	15751	6679.414	P. mirabilis
GCF_010442675.1	GCF_011383025.1	15772	6688.31932	P. mirabilis
GCF_000967845.1	GCF_001006265.1	15775	5262.05887	K. pneumoniae
GCF_000967845.1	GCF_016859025.1	15804	5271.73239	K. pneumoniae
GCF_000967845.1	GCF_019284495.1	15806	5272.39953	K. pneumoniae
GCF_010442675.1	GCF_008041895.1	15830	6712.91496	P. mirabilis
GCF_009429045.2	GCF_008041895.1	15830	6712.91496	P. mirabilis
GCF_008195605.1	GCF_008041895.1	15832	6713.76309	P. mirabilis
GCF_010442675.1	GCF_003030945.1	15853	6722.66841	P. mirabilis
GCF_016864435.1	GCF_014898635.1	15863	15643.9996	E. coli
GCF_009429045.2	GCF_014931585.1	15864	6727.3331	P. mirabilis
GCF_010442675.1	GCF_002055685.1	15868	6729.02935	P. mirabilis
GCF_010442675.1	GCF_014931585.1	15870	6729.87748	P. mirabilis
GCF_010442675.1	GCF_000069965.1	15873	6731.14966	P. mirabilis
GCF_000444425.1	GCF_002055685.1	15878	6733.26998	P. mirabilis
GCF_010442675.1	GCF_002310875.1	15886	6736.66248	P. mirabilis
GCF_000444425.1	GCF_003030945.1	15898	6741.75124	P. mirabilis
GCF_008195605.1	GCF_000069965.1	15901	6743.02342	P. mirabilis
GCF_000967845.1	GCF_002211665.1	15909	5306.75718	K. pneumoniae
GCF_016864435.1	GCF_007107705.1	15910	15690.3508	E. coli
GCF_000444425.1	GCF_000069965.1	15911	6747.26405	P. mirabilis
GCF_008195605.1	GCF_014931585.1	15912	6747.68812	P. mirabilis
GCF_009429045.2	GCF_011383025.1	15914	6748.53624	P. mirabilis
GCF_000967845.1	GCF_000240185.1	15915	5308.7586	K. pneumoniae
GCF_010442675.1	GCF_013343255.1	15916	6749.38437	P. mirabilis
GCF_008195605.1	GCF_003030945.1	15928	6754.47312	P. mirabilis
GCF_000444425.1	GCF_008041895.1	15935	6757.44156	P. mirabilis
GCF_009429045.2	GCF_002055685.1	15940	6759.56188	P. mirabilis
GCF_000444425.1	GCF_014931585.1	15945	6761.68219	P. mirabilis
GCF_010442675.1	GCF_008630655.1	15945	6761.68219	P. mirabilis
GCF_000444425.1	GCF_011045575.1	15946	6762.10625	P. mirabilis
GCF_010442675.1	GCF_011045575.1	15946	6762.10625	P. mirabilis
GCF_009429045.2	GCF_008630655.1	15953	6765.07469	P. mirabilis
GCF_010442675.1	GCF_001640985.1	15955	6765.92282	P. mirabilis
GCF_008195605.1	GCF_011045575.1	15956	6766.34688	P. mirabilis
GCF_000967845.1	GCF_003815075.1	15958	5323.10209	K. pneumoniae
GCF_008195605.1	GCF_002310875.1	15971	6772.70782	P. mirabilis
GCF_008195605.1	GCF_002055685.1	15971	6772.70782	P. mirabilis
GCF_000444425.1	GCF_002310875.1	15980	6776.52439	P. mirabilis
GCF_000967845.1	GCF_009684615.1	15984	5331.7749	K. pneumoniae
GCF_009429045.2	GCF_003030945.1	15986	6779.06877	P. mirabilis
GCF_006514375.1	GCF_014898635.1	15988	15767.2739	E. coli
GCF_009429045.2	GCF_000069965.1	15988	6779.91689	P. mirabilis
GCF_008195605.1	GCF_013343255.1	16001	6785.42971	P. mirabilis
GCF_000444425.1	GCF_013343255.1	16010	6789.24627	P. mirabilis
GCF_009429045.2	GCF_002310875.1	16025	6795.60722	P. mirabilis
GCF_006514375.1	GCF_007107705.1	16036	15814.6113	E. coli
GCF_013706045.1	GCF_003963495.1	16042	5351.12193	K. pneumoniae
GCF_008195605.1	GCF_001640985.1	16043	6803.24035	P. mirabilis
GCF_000444425.1	GCF_001640985.1	16049	6805.78473	P. mirabilis
GCF_000444425.1	GCF_008630655.1	16052	6807.05691	P. mirabilis
GCF_009429045.2	GCF_013343255.1	16055	6808.3291	P. mirabilis
GCF_008195605.1	GCF_008630655.1	16071	6815.11411	P. mirabilis
GCF_009429045.2	GCF_001640985.1	16075	6816.81036	P. mirabilis
GCF_009429045.2	GCF_011045575.1	16080	6818.93067	P. mirabilis
GCF_000967845.1	GCF_003963495.1	16120	5377.14035	K. pneumoniae
GCF_000967845.1	GCF_900069965.1_KPN_RH201207_genomic	16213	5408.16231	K. pneumoniae
GCF_002634895.1	GCF_004138625.1	16222	15998.0434	E. coli
GCF_003666405.1	GCF_004138625.1	16244	16019.7397	E. coli
GCF_003112045.1	GCF_004138625.1	16266	16041.4359	E. coli
GCF_002634895.1	GCF_003194125.1	16276	16051.2979	E. coli
GCF_010442675.1	GCF_014843115.1	16283	6905.01543	P. mirabilis
GCF_002736085.1	GCF_004138625.1	16287	16062.146	E. coli
GCF_003666405.1	GCF_003194125.1	16288	16063.1322	E. coli
GCF_000166535.2	GCF_004138625.1	16292	16067.077	E. coli
GCF_000444425.1	GCF_014843115.1	16304	6913.92075	P. mirabilis
GCF_003112045.1	GCF_003194125.1	16353	16127.2348	E. coli
GCF_002736085.1	GCF_003194125.1	16355	16129.2072	E. coli
GCF_004358945.1	GCF_003194125.1	16367	16141.0416	E. coli
GCF_008195605.1	GCF_014843115.1	16376	6944.45328	P. mirabilis
GCF_003072445.1	GCF_004138625.1	16410	16183.4479	E. coli
GCF_000166535.2	GCF_003194125.1	16415	16188.3789	E. coli
GCF_010442675.1	GCF_018972025.1	16423	6964.38423	P. mirabilis
GCF_002879735.1	GCF_004138625.1	16457	16229.799	E. coli
GCF_000444425.1	GCF_018972025.1	16459	6979.6505	P. mirabilis
GCF_014216355.1	GCF_004138625.1	16461	16233.7438	E. coli
GCF_016801475.1	GCF_004138625.1	16471	16243.6058	E. coli
GCF_009429045.2	GCF_014843115.1	16473	6985.58738	P. mirabilis
GCF_003367575.1	GCF_004138625.1	16481	16253.4677	E. coli

GCF_008195605.1	GCF_018972025.1	16482	6989.40394	P. mirabilis
GCF_016801475.1	GCF_003194125.1	16487	16259.3849	E. coli
GCF_003017885.1	GCF_004138625.1	16490	16262.3435	E. coli
GCF_004358945.1	GCF_004138625.1	16495	16267.2744	E. coli
GCF_003017885.1	GCF_003194125.1	16500	16272.2054	E. coli
GCF_014216355.1	GCF_003194125.1	16510	16282.0673	E. coli
GCF_002879735.1	GCF_003194125.1	16523	16294.8879	E. coli
GCF_019614135.1	GCF_004138625.1	16532	16303.7636	E. coli
GCF_003367575.1	GCF_003194125.1	16535	16306.7222	E. coli
GCF_018208275.1	GCF_004138625.1	16545	16316.5841	E. coli
GCF_019614135.1	GCF_003194125.1	16601	16371.811	E. coli
GCF_009429045.2	GCF_018972025.1	16612	7044.53211	P. mirabilis
GCF_003072445.1	GCF_003194125.1	16630	16400.4107	E. coli
GCF_016864435.1	GCF_004138625.1	16790	16558.2017	E. coli
GCF_018208275.1	GCF_003194125.1	16816	16583.8428	E. coli
GCF_016864435.1	GCF_003194125.1	16873	16640.0559	E. coli
GCF_006514375.1	GCF_004138625.1	16920	16686.407	E. coli
GCF_006514375.1	GCF_003194125.1	17002	16767.2749	E. coli
GCF_003194305.1	GCF_011045575.1	17031	7222.21445	P. mirabilis
GCF_003194305.1	GCF_000069965.1	17283	7329.07829	P. mirabilis
GCF_003194305.1	GCF_011149675.1	17305	7338.40767	P. mirabilis
GCF_003194305.1	GCF_011383025.1	17347	7356.21831	P. mirabilis
GCF_003194305.1	GCF_014931585.1	17423	7388.44709	P. mirabilis
GCF_003194305.1	GCF_008630655.1	17427	7390.14334	P. mirabilis
GCF_003194305.1	GCF_000783575.2	17466	7406.68179	P. mirabilis
GCF_003194305.1	GCF_002310875.1	17467	7407.10585	P. mirabilis
GCF_003194305.1	GCF_002055685.1	17472	7409.22617	P. mirabilis
GCF_003194305.1	GCF_013343255.1	17495	7418.97961	P. mirabilis
GCF_012955465.1	GCF_014898635.1	17498	17256.4273	E. coli
GCF_003194305.1	GCF_001640985.1	17535	7435.94213	P. mirabilis
GCF_003194305.1	GCF_018138945.1	17538	7437.21431	P. mirabilis
GCF_003999775.1	GCF_014898635.1	17556	17313.6265	E. coli
GCF_003028695.1	GCF_014898635.1	17583	17340.2538	E. coli
GCF_013344585.1	GCF_014898635.1	17598	17355.0467	E. coli
GCF_003194305.1	GCF_019443785.1	17622	7472.83559	P. mirabilis
GCF_003194305.1	GCF_008041895.1	17637	7479.19654	P. mirabilis
GCF_003194305.1	GCF_018336495.1	17644	7482.16498	P. mirabilis
GCF_018986775.1	GCF_014898635.1	17653	17409.2874	E. coli
GCF_003194305.1	GCF_014843115.1	17687	7500.39968	P. mirabilis
GCF_003194305.1	GCF_019192645.1	17730	7518.63438	P. mirabilis
GCF_902164685.1_25426_7_120_genomic	GCF_014898635.1	17759	17513.824	E. coli
GCF_003194305.1	GCF_002945235.1	17778	7538.9894	P. mirabilis
GCF_003194305.1	GCF_003073935.1	17805	7550.4391	P. mirabilis
GCF_003194305.1	GCF_018972025.1	17840	7565.2813	P. mirabilis
GCF_012955465.1	GCF_001938625.2	17873	17626.2501	E. coli
GCF_018986775.1	GCF_001938625.2	17884	17637.0983	E. coli
GCF_003999775.1	GCF_001938625.2	17913	17665.6979	E. coli
GCF_003028695.1	GCF_001938625.2	17939	17691.3389	E. coli
GCF_902164685.1_25426_7_120_genomic	GCF_001938625.2	17942	17694.2975	E. coli
GCF_003999775.1	GCF_002125925.1	17945	17697.2561	E. coli
GCF_013344585.1	GCF_001938625.2	17956	17708.1042	E. coli
GCF_003028695.1	GCF_002125925.1	17972	17723.8834	E. coli
GCF_013344585.1	GCF_002125925.1	17987	17738.6763	E. coli
GCF_003999775.1	GCF_002634895.1	17999	17750.5106	E. coli
GCF_012955465.1	GCF_007107705.1	18022	17773.1931	E. coli
GCF_003028695.1	GCF_002634895.1	18026	17777.1378	E. coli
GCF_003999775.1	GCF_019614135.1	18038	17788.9722	E. coli
GCF_013344585.1	GCF_002634895.1	18041	17791.9308	E. coli
GCF_003194305.1	GCF_003030945.1	18041	7650.51793	P. mirabilis
GCF_018986775.1	GCF_002125925.1	18061	17811.6546	E. coli
GCF_003028695.1	GCF_019614135.1	18063	17813.627	E. coli
GCF_013344585.1	GCF_019614135.1	18078	17828.4199	E. coli
GCF_018986775.1	GCF_007107705.1	18083	17833.3509	E. coli
GCF_012955465.1	GCF_002125925.1	18096	17846.1714	E. coli
GCF_902164685.1_25426_7_120_genomic	GCF_007107705.1	18176	17925.067	E. coli
GCF_003999775.1	GCF_000166535.2	18181	17929.998	E. coli
GCF_003999775.1	GCF_007107705.1	18184	17932.9565	E. coli
GCF_003999775.1	GCF_014216355.1	18200	17948.7356	E. coli
GCF_003999775.1	GCF_003666405.1	18203	17951.6942	E. coli
GCF_003999775.1	GCF_003367575.1	18206	17954.6528	E. coli
GCF_003028695.1	GCF_000166535.2	18208	17956.6252	E. coli
GCF_018986775.1	GCF_019614135.1	18208	17956.6252	E. coli
GCF_003028695.1	GCF_007107705.1	18210	17958.5976	E. coli
GCF_012955465.1	GCF_019614135.1	18212	17960.57	E. coli
GCF_012955465.1	GCF_002634895.1	18213	17961.5562	E. coli
GCF_003999775.1	GCF_003112045.1	18215	17963.5286	E. coli
GCF_018986775.1	GCF_002634895.1	18217	17965.501	E. coli
GCF_013344585.1	GCF_000166535.2	18223	17971.4181	E. coli
GCF_003028695.1	GCF_014216355.1	18225	17973.3905	E. coli
GCF_003999775.1	GCF_004358945.1	18225	17973.3905	E. coli
GCF_013344585.1	GCF_007107705.1	18226	17974.3767	E. coli
GCF_003028695.1	GCF_003666405.1	18227	17975.3629	E. coli
GCF_003028695.1	GCF_003367575.1	18231	17979.3077	E. coli
GCF_013344585.1	GCF_014216355.1	18239	17987.1972	E. coli
GCF_003028695.1	GCF_003112045.1	18239	17987.1972	E. coli
GCF_003999775.1	GCF_003072445.1	18242	17990.1558	E. coli
GCF_013344585.1	GCF_003666405.1	18243	17991.142	E. coli
GCF_013344585.1	GCF_003367575.1	18245	17993.1144	E. coli
GCF_003999775.1	GCF_018208275.1	18250	17998.0454	E. coli
GCF_003028695.1	GCF_004358945.1	18252	18000.0178	E. coli
GCF_013344585.1	GCF_003112045.1	18255	18002.9763	E. coli
GCF_902164685.1_25426_7_120_genomic	GCF_002125925.1	18258	18005.9349	E. coli
GCF_003028695.1	GCF_003072445.1	18267	18014.8107	E. coli
GCF_013344585.1	GCF_004358945.1	18267	18014.8107	E. coli

GCF_003999775.1	GCF_016801475.1	18272	18019.7416	E. coli
GCF_003028695.1	GCF_018208275.1	18275	18022.7002	E. coli
GCF_003999775.1	GCF_002879735.1	18276	18023.6864	E. coli
GCF_013344585.1	GCF_003072445.1	18282	18029.6036	E. coli
GCF_013344585.1	GCF_018208275.1	18290	18037.4931	E. coli
GCF_902164685.1_25426_7_120_genomic	GCF_019614135.1	18291	18038.4793	E. coli
GCF_003999775.1	GCF_002736085.1	18296	18043.4103	E. coli
GCF_003028695.1	GCF_016801475.1	18297	18044.3965	E. coli
GCF_003028695.1	GCF_002879735.1	18301	18048.3413	E. coli
GCF_013344585.1	GCF_016801475.1	18312	18059.1894	E. coli
GCF_013344585.1	GCF_002879735.1	18316	18063.1342	E. coli
GCF_003028695.1	GCF_002736085.1	18320	18067.079	E. coli
GCF_018986775.1	GCF_004358945.1	18327	18073.9823	E. coli
GCF_018986775.1	GCF_000166535.2	18334	18080.8857	E. coli
GCF_013344585.1	GCF_002736085.1	18336	18082.8581	E. coli
GCF_902164685.1_25426_7_120_genomic	GCF_002634895.1	18345	18091.7338	E. coli
GCF_012955465.1	GCF_004358945.1	18349	18095.6786	E. coli
GCF_018986775.1	GCF_014216355.1	18356	18102.582	E. coli
GCF_012955465.1	GCF_014216355.1	18358	18104.5543	E. coli
GCF_012955465.1	GCF_003367575.1	18361	18107.5129	E. coli
GCF_003999775.1	GCF_003017885.1	18362	18108.4991	E. coli
GCF_018986775.1	GCF_003367575.1	18363	18109.4853	E. coli
GCF_012955465.1	GCF_000166535.2	18365	18111.4577	E. coli
GCF_012955465.1	GCF_003666405.1	18378	18124.2782	E. coli
GCF_018986775.1	GCF_003666405.1	18381	18127.2368	E. coli
GCF_003028695.1	GCF_003017885.1	18387	18133.154	E. coli
GCF_018986775.1	GCF_003112045.1	18390	18136.1126	E. coli
GCF_003999775.1	GCF_016864435.1	18401	18146.9607	E. coli
GCF_013344585.1	GCF_003017885.1	18402	18147.9469	E. coli
GCF_012955465.1	GCF_016801475.1	18403	18148.9331	E. coli
GCF_012955465.1	GCF_003112045.1	18408	18153.8641	E. coli
GCF_018986775.1	GCF_002879735.1	18423	18168.657	E. coli
GCF_003028695.1	GCF_016864435.1	18424	18169.6432	E. coli
GCF_012955465.1	GCF_002879735.1	18426	18171.6156	E. coli
GCF_012955465.1	GCF_002736085.1	18427	18172.6017	E. coli
GCF_013344585.1	GCF_016864435.1	18440	18185.4223	E. coli
GCF_902164685.1_25426_7_120_genomic	GCF_014216355.1	18440	18185.4223	E. coli
GCF_902164685.1_25426_7_120_genomic	GCF_003367575.1	18449	18194.298	E. coli
GCF_018986775.1	GCF_002736085.1	18457	18202.1876	E. coli
GCF_902164685.1_25426_7_120_genomic	GCF_004358945.1	18461	18206.1324	E. coli
GCF_018986775.1	GCF_018208275.1	18464	18209.0909	E. coli
GCF_902164685.1_25426_7_120_genomic	GCF_016801475.1	18466	18211.0633	E. coli
GCF_012955465.1	GCF_018208275.1	18467	18212.0495	E. coli
GCF_902164685.1_25426_7_120_genomic	GCF_018208275.1	18469	18214.0219	E. coli
GCF_012955465.1	GCF_003072445.1	18470	18215.0081	E. coli
GCF_902164685.1_25426_7_120_genomic	GCF_003666405.1	18474	18218.9529	E. coli
GCF_902164685.1_25426_7_120_genomic	GCF_000166535.2	18484	18228.8148	E. coli
GCF_902164685.1_25426_7_120_genomic	GCF_003112045.1	18491	18235.7182	E. coli
GCF_018986775.1	GCF_003072445.1	18491	18235.7182	E. coli
GCF_012955465.1	GCF_016864435.1	18499	18243.6077	E. coli
GCF_018986775.1	GCF_016801475.1	18499	18243.6077	E. coli
GCF_902164685.1_25426_7_120_genomic	GCF_002879735.1	18519	18263.3316	E. coli
GCF_003999775.1	GCF_006514375.1	18532	18276.1521	E. coli
GCF_003028695.1	GCF_006514375.1	18555	18298.8346	E. coli
GCF_012955465.1	GCF_003017885.1	18560	18303.7656	E. coli
GCF_902164685.1_25426_7_120_genomic	GCF_002736085.1	18561	18304.7518	E. coli
GCF_018986775.1	GCF_003017885.1	18566	18309.6828	E. coli
GCF_013344585.1	GCF_006514375.1	18571	18314.6137	E. coli
GCF_018986775.1	GCF_016864435.1	18583	18326.4481	E. coli
GCF_902164685.1_25426_7_120_genomic	GCF_003072445.1	18591	18334.3376	E. coli
GCF_012955465.1	GCF_006514375.1	18630	18372.7992	E. coli
GCF_902164685.1_25426_7_120_genomic	GCF_003017885.1	18654	18396.4678	E. coli
GCF_003194305.1	GCF_000444425.1	18657	7911.74065	P. mirabilis
GCF_902164685.1_25426_7_120_genomic	GCF_016864435.1	18684	18426.0537	E. coli
GCF_018986775.1	GCF_006514375.1	18716	18457.6119	E. coli
GCF_003194305.1	GCF_008195605.1	18739	7946.5138	P. mirabilis
GCF_003194305.1	GCF_010442675.1	18751	7951.60255	P. mirabilis
GCF_902164685.1_25426_7_120_genomic	GCF_006514375.1	18813	18553.2727	E. coli
GCF_003194305.1	GCF_009429045.2	18843	7990.61634	P. mirabilis
GCF_000967845.1	GCF_013706045.1	19248	6420.54575	K. pneumoniae
GCF_013282235.1	GCF_001051055.1	21661	30507.8491	A. xylosoxidans/A. insolitus
GCF_001457475.1_NCTC10807_genomic	GCF_001051055.1	21666	30514.8913	A. xylosoxidans/A. insolitus
GCF_000967095.2_A.x.NH44784-1996_genomic	GCF_001051055.1	21681	30536.0176	A. xylosoxidans/A. insolitus
GCF_900009115.1_BN2877_genomic	GCF_001051055.1	21681	30536.0176	A. xylosoxidans/A. insolitus
GCF_000508285.1	GCF_001051055.1	21706	30571.2282	A. xylosoxidans/A. insolitus
GCF_008432465.1	GCF_001051055.1	21777	30671.2262	A. xylosoxidans/A. insolitus
GCF_000165835.1	GCF_014490035.1	28431	40042.8724	A. xylosoxidans/A. insolitus
GCF_009363015.1	GCF_904423855.1_G1433_genomic	35766	50373.6546	A. xylosoxidans/A. insolitus
GCF_001639685.1	GCF_014490035.1	39279	55321.4444	A. xylosoxidans/A. insolitus
GCF_022325125.1	GCF_014490035.1	39285	55329.8949	A. xylosoxidans/A. insolitus
GCF_003293535.1	GCF_014490035.1	39286	55331.3033	A. xylosoxidans/A. insolitus
GCF_008245125.1	GCF_014490035.1	39323	55383.415	A. xylosoxidans/A. insolitus
GCF_011063385.1	GCF_014490035.1	39373	55453.8361	A. xylosoxidans/A. insolitus
GCF_001641515.1	GCF_014490035.1	39381	55465.1035	A. xylosoxidans/A. insolitus
GCF_002209555.1	GCF_014490035.1	39389	55476.3709	A. xylosoxidans/A. insolitus
GCF_000783435.2	GCF_014490035.1	39392	55480.5962	A. xylosoxidans/A. insolitus
GCF_001971645.1	GCF_014490035.1	39395	55484.8214	A. xylosoxidans/A. insolitus
GCF_902860205.1_LMG_6001_genomic	GCF_014490035.1	39397	55487.6383	A. xylosoxidans/A. insolitus
GCF_003293535.1	GCF_000165835.1	40227	56656.6293	A. xylosoxidans/A. insolitus
GCF_001639685.1	GCF_000165835.1	40247	56684.7978	A. xylosoxidans/A. insolitus
GCF_008245125.1	GCF_000165835.1	40295	56752.4021	A. xylosoxidans/A. insolitus
GCF_022325125.1	GCF_000165835.1	40296	56753.8105	A. xylosoxidans/A. insolitus
GCF_002209555.1	GCF_000165835.1	40339	56814.3727	A. xylosoxidans/A. insolitus
GCF_000783435.2	GCF_000165835.1	40340	56815.7811	A. xylosoxidans/A. insolitus

GCF_001971645.1	GCF_000165835.1	40345	56822.8232	A. xylosoxidans/A. insolitus
GCF_011063385.1	GCF_000165835.1	40347	56825.6401	A. xylosoxidans/A. insolitus
GCF_001641515.1	GCF_000165835.1	40350	56829.8653	A. xylosoxidans/A. insolitus
GCF_902860205.1_LMG_6001_genomic	GCF_000165835.1	40379	56870.7096	A. xylosoxidans/A. insolitus
GCF_001457475.1_NCTC10807_genomic	GCF_014490035.1	53264	75018.2391	A. xylosoxidans/A. insolitus
GCF_008432465.1	GCF_014490035.1	53275	75033.7317	A. xylosoxidans/A. insolitus
GCF_000508285.1	GCF_014490035.1	53304	75074.576	A. xylosoxidans/A. insolitus
GCF_900009115.1_BN2877_genomic	GCF_014490035.1	53340	75125.2792	A. xylosoxidans/A. insolitus
GCF_013282235.1	GCF_014490035.1	53432	75254.8541	A. xylosoxidans/A. insolitus
GCF_000967095.2_A.x.NH44784-1996_genomic	GCF_014490035.1	53560	75435.1323	A. xylosoxidans/A. insolitus
GCF_000017205.1	GCF_000981825.1	53716	38159.81	P. aeruginosa
GCF_000017205.1	GCF_900497025.1	53719	38161.9412	P. aeruginosa
GCF_001051055.1	GCF_014490035.1	53770	75730.9011	A. xylosoxidans/A. insolitus
GCF_000017205.1	GCF_001874465.1	53943	38321.0707	P. aeruginosa
GCF_000017205.1	GCF_000284555.1	54068	38409.8706	P. aeruginosa
GCF_000017205.1	GCF_001722005.2	54073	38413.4226	P. aeruginosa
GCF_001457475.1_NCTC10807_genomic	GCF_000165835.1	54090	76181.5964	A. xylosoxidans/A. insolitus
GCF_008432465.1	GCF_000165835.1	54091	76183.0048	A. xylosoxidans/A. insolitus
GCF_000017205.1	GCF_002287725.2	54126	38451.0737	P. aeruginosa
GCF_000508285.1	GCF_000165835.1	54133	76242.1586	A. xylosoxidans/A. insolitus
GCF_900009115.1_BN2877_genomic	GCF_000165835.1	54201	76337.9314	A. xylosoxidans/A. insolitus
GCF_000017205.1	GCF_001293085.1	54250	38539.1632	P. aeruginosa
GCF_000017205.1	GCF_000496605.2	54250	38539.1632	P. aeruginosa
GCF_000017205.1	GCF_900095805.1_PA14Or_genomic	54262	38547.688	P. aeruginosa
GCF_000017205.1	GCF_000014625.1	54272	38554.792	P. aeruginosa
GCF_000017205.1	GCF_009648875.1	54278	38559.0544	P. aeruginosa
GCF_000017205.1	GCF_002223805.1	54287	38565.448	P. aeruginosa
GCF_000017205.1	GCF_016126955.1	54288	38566.1584	P. aeruginosa
GCF_013282235.1	GCF_000165835.1	54302	76480.1821	A. xylosoxidans/A. insolitus
GCF_000017205.1	GCF_900070375.1_PAO1OR_genomic	54307	38579.656	P. aeruginosa
GCF_000017205.1	GCF_013305765.1	54307	38579.656	P. aeruginosa
GCF_000017205.1	GCF_004014755.1	54310	38581.7872	P. aeruginosa
GCF_000017205.1	GCF_014792125.1	54312	38583.208	P. aeruginosa
GCF_000017205.1	GCF_004102665.1	54317	38586.76	P. aeruginosa
GCF_000017205.1	GCF_900149285.1_PcyII-10_genomic	54318	38587.4704	P. aeruginosa
GCF_000017205.1	GCF_019466145.1	54335	38599.5472	P. aeruginosa
GCF_000017205.1	GCF_001045685.1	54337	38600.968	P. aeruginosa
GCF_000017205.1	GCF_003204335.1	54338	38601.6784	P. aeruginosa
GCF_000017205.1	GCF_018409365.1	54341	38603.8096	P. aeruginosa
GCF_000017205.1	GCF_002968515.1	54343	38605.2304	P. aeruginosa
GCF_000017205.1	GCF_014854655.1	54348	38608.7824	P. aeruginosa
GCF_000017205.1	GCF_009911735.1	54353	38612.3344	P. aeruginosa
GCF_000017205.1	GCF_000226155.1	54355	38613.7552	P. aeruginosa
GCF_000017205.1	GCF_000271985.2	54361	38618.0176	P. aeruginosa
GCF_000017205.1	GCF_000414035.1	54364	38620.1488	P. aeruginosa
GCF_000017205.1	GCF_000568855.2	54369	38623.7008	P. aeruginosa
GCF_000017205.1	GCF_008033725.1	54378	38630.0944	P. aeruginosa
GCF_000017205.1	GCF_012935295.1	54382	38632.936	P. aeruginosa
GCF_000967095.2_A.x.NH44784-1996_genomic	GCF_000165835.1	54384	76595.6728	A. xylosoxidans/A. insolitus
GCF_000017205.1	GCF_003571505.1	54384	38634.3568	P. aeruginosa
GCF_000017205.1	GCF_000524595.1	54391	38639.3296	P. aeruginosa
GCF_000017205.1	GCF_900243355.1_RW109_genomic	54395	38642.1712	P. aeruginosa
GCF_000017205.1	GCF_904866275.1_MINF_7A_genomic	54407	38650.6959	P. aeruginosa
GCF_000017205.1	GCF_016105505.1	54410	38652.8271	P. aeruginosa
GCF_000017205.1	GCF_001606045.1	54421	38660.6415	P. aeruginosa
GCF_000017205.1	GCF_001879525.1	54440	38674.1391	P. aeruginosa
GCF_000017205.1	GCF_000026645.1	54441	38674.8495	P. aeruginosa
GCF_000017205.1	GCF_001900195.1	54442	38675.5599	P. aeruginosa
GCF_000017205.1	GCF_003319235.1	54444	38676.9807	P. aeruginosa
GCF_000017205.1	GCF_900636735.1_43941_C01_genomic	54445	38677.6911	P. aeruginosa
GCF_000017205.1	GCF_001750705.1	54447	38679.1119	P. aeruginosa
GCF_001051055.1	GCF_000165835.1	54468	76713.9803	A. xylosoxidans/A. insolitus
GCF_016743035.1	GCF_900497025.1	54590	38780.699	P. aeruginosa
GCF_016743035.1	GCF_000981825.1	54593	38782.8302	P. aeruginosa
GCF_016743035.1	GCF_001874465.1	54623	38804.1422	P. aeruginosa
GCF_003025345.2	GCF_900497025.1	54676	38841.7934	P. aeruginosa
GCF_003025345.2	GCF_000981825.1	54679	38843.9246	P. aeruginosa
GCF_006971785.1	GCF_900497025.1	54742	38888.6797	P. aeruginosa
GCF_006971785.1	GCF_000981825.1	54745	38890.8109	P. aeruginosa
GCF_006971785.1	GCF_001874465.1	54752	38895.7837	P. aeruginosa
GCF_016743035.1	GCF_000284555.1	54777	38913.5437	P. aeruginosa
GCF_016743035.1	GCF_001722005.2	54788	38921.3581	P. aeruginosa
GCF_003025345.2	GCF_001874465.1	54794	38925.6205	P. aeruginosa
GCF_016743035.1	GCF_002287725.2	54835	38954.7469	P. aeruginosa
GCF_016743035.1	GCF_900095805.1_PA14Or_genomic	54869	38978.9004	P. aeruginosa
GCF_016743035.1	GCF_000014625.1	54879	38986.0044	P. aeruginosa
GCF_016743035.1	GCF_002223805.1	54894	38996.6604	P. aeruginosa
GCF_006971785.1	GCF_001722005.2	54901	39001.6332	P. aeruginosa
GCF_003025345.2	GCF_001722005.2	54919	39014.4204	P. aeruginosa
GCF_003025345.2	GCF_000284555.1	54920	39015.1308	P. aeruginosa
GCF_006971785.1	GCF_000284555.1	54931	39022.9452	P. aeruginosa
GCF_006971785.1	GCF_002287725.2	54946	39033.6012	P. aeruginosa
GCF_016743035.1	GCF_001293085.1	54946	39033.6012	P. aeruginosa
GCF_016743035.1	GCF_000496605.2	54946	39033.6012	P. aeruginosa
GCF_016743035.1	GCF_016126955.1	54964	39046.3884	P. aeruginosa
GCF_003025345.2	GCF_002287725.2	54966	39047.8092	P. aeruginosa
GCF_016743035.1	GCF_900070375.1_PAO1OR_genomic	54993	39066.9899	P. aeruginosa
GCF_016743035.1	GCF_013305765.1	54993	39066.9899	P. aeruginosa
GCF_016743035.1	GCF_004014755.1	54996	39069.1211	P. aeruginosa
GCF_016743035.1	GCF_004102665.1	55003	39074.0939	P. aeruginosa
GCF_016743035.1	GCF_019466145.1	55007	39076.9355	P. aeruginosa
GCF_016743035.1	GCF_900149285.1_PcyII-10_genomic	55009	39078.3563	P. aeruginosa
GCF_016743035.1	GCF_001045685.1	55010	39079.0667	P. aeruginosa
GCF_016743035.1	GCF_003204335.1	55024	39089.0123	P. aeruginosa

GCF_016743035.1	GCF_009648875.1	55026	39090.4331	P. aeruginosa
GCF_016743035.1	GCF_002968515.1	55027	39091.1435	P. aeruginosa
GCF_016743035.1	GCF_018409365.1	55041	39101.0891	P. aeruginosa
GCF_016743035.1	GCF_000271985.2	55043	39102.5099	P. aeruginosa
GCF_016743035.1	GCF_000226155.1	55044	39103.2203	P. aeruginosa
GCF_006971785.1	GCF_900095805.1_PA14Or_genomic	55045	39103.9307	P. aeruginosa
GCF_016743035.1	GCF_014792125.1	55047	39105.3515	P. aeruginosa
GCF_016743035.1	GCF_000414035.1	55053	39109.6139	P. aeruginosa
GCF_006971785.1	GCF_000014625.1	55055	39111.0347	P. aeruginosa
GCF_016743035.1	GCF_009911735.1	55060	39114.5867	P. aeruginosa
GCF_006971785.1	GCF_002223805.1	55070	39121.6907	P. aeruginosa
GCF_003025345.2	GCF_900095805.1_PA14Or_genomic	55075	39125.2427	P. aeruginosa
GCF_016743035.1	GCF_012935295.1	55075	39125.2427	P. aeruginosa
GCF_016743035.1	GCF_016105505.1	55084	39131.6363	P. aeruginosa
GCF_003025345.2	GCF_000014625.1	55085	39132.3467	P. aeruginosa
GCF_016743035.1	GCF_008033725.1	55085	39132.3467	P. aeruginosa
GCF_016743035.1	GCF_000524595.1	55087	39133.7675	P. aeruginosa
GCF_016743035.1	GCF_000568855.2	55089	39135.1883	P. aeruginosa
GCF_016743035.1	GCF_014854655.1	55090	39135.8987	P. aeruginosa
GCF_016743035.1	GCF_001900195.1	55092	39137.3195	P. aeruginosa
GCF_006971785.1	GCF_001293085.1	55098	39141.5819	P. aeruginosa
GCF_006971785.1	GCF_000496605.2	55098	39141.5819	P. aeruginosa
GCF_003025345.2	GCF_002223805.1	55100	39143.0027	P. aeruginosa
GCF_016743035.1	GCF_904866275.1_MINF_7A_genomic	55101	39143.7131	P. aeruginosa
GCF_016743035.1	GCF_003319235.1	55121	39157.9211	P. aeruginosa
GCF_016743035.1	GCF_001606045.1	55122	39158.6315	P. aeruginosa
GCF_016743035.1	GCF_900636735.1_43941_C01_genomic	55123	39159.3419	P. aeruginosa
GCF_016743035.1	GCF_001750705.1	55124	39160.0523	P. aeruginosa
GCF_006971785.1	GCF_014792125.1	55134	39167.1563	P. aeruginosa
GCF_003025345.2	GCF_001293085.1	55136	39168.577	P. aeruginosa
GCF_003025345.2	GCF_000496605.2	55136	39168.577	P. aeruginosa
GCF_016743035.1	GCF_001879525.1	55138	39169.9978	P. aeruginosa
GCF_016743035.1	GCF_003571505.1	55143	39173.5498	P. aeruginosa
GCF_016743035.1	GCF_000026645.1	55144	39174.2602	P. aeruginosa
GCF_006971785.1	GCF_016126955.1	55145	39174.9706	P. aeruginosa
GCF_003025345.2	GCF_001045685.1	55146	39175.681	P. aeruginosa
GCF_003025345.2	GCF_016126955.1	55154	39181.3642	P. aeruginosa
GCF_003025345.2	GCF_014792125.1	55155	39182.0746	P. aeruginosa
GCF_006971785.1	GCF_001045685.1	55156	39182.785	P. aeruginosa
GCF_016743035.1	GCF_900243355.1_RW109_genomic	55159	39184.9162	P. aeruginosa
GCF_006971785.1	GCF_019466145.1	55180	39199.8346	P. aeruginosa
GCF_006971785.1	GCF_900070375.1_PAO1OR_genomic	55182	39201.2554	P. aeruginosa
GCF_006971785.1	GCF_013305765.1	55182	39201.2554	P. aeruginosa
GCF_006971785.1	GCF_014854655.1	55183	39201.9658	P. aeruginosa
GCF_006971785.1	GCF_004014755.1	55185	39203.3866	P. aeruginosa
GCF_003025345.2	GCF_014854655.1	55188	39205.5178	P. aeruginosa
GCF_003025345.2	GCF_900070375.1_PAO1OR_genomic	55189	39206.2282	P. aeruginosa
GCF_003025345.2	GCF_013305765.1	55189	39206.2282	P. aeruginosa
GCF_006971785.1	GCF_009648875.1	55189	39206.2282	P. aeruginosa
GCF_003025345.2	GCF_004014755.1	55192	39208.3594	P. aeruginosa
GCF_006971785.1	GCF_004102665.1	55192	39208.3594	P. aeruginosa
GCF_006971785.1	GCF_003204335.1	55196	39211.201	P. aeruginosa
GCF_003025345.2	GCF_009648875.1	55197	39211.9114	P. aeruginosa
GCF_003025345.2	GCF_004102665.1	55199	39213.3322	P. aeruginosa
GCF_003025345.2	GCF_019466145.1	55199	39213.3322	P. aeruginosa
GCF_006971785.1	GCF_002968515.1	55199	39213.3322	P. aeruginosa
GCF_006971785.1	GCF_000226155.1	55206	39218.305	P. aeruginosa
GCF_003025345.2	GCF_900149285.1_PcyII-10_genomic	55206	39218.305	P. aeruginosa
GCF_003025345.2	GCF_000226155.1	55207	39219.0154	P. aeruginosa
GCF_006971785.1	GCF_900149285.1_PcyII-10_genomic	55207	39219.0154	P. aeruginosa
GCF_006971785.1	GCF_018409365.1	55212	39222.5674	P. aeruginosa
GCF_003025345.2	GCF_003204335.1	55217	39226.1194	P. aeruginosa
GCF_003025345.2	GCF_002968515.1	55220	39228.2506	P. aeruginosa
GCF_003025345.2	GCF_018409365.1	55221	39228.961	P. aeruginosa
GCF_006971785.1	GCF_009911735.1	55227	39233.2234	P. aeruginosa
GCF_006971785.1	GCF_000271985.2	55230	39235.3546	P. aeruginosa
GCF_003025345.2	GCF_003571505.1	55234	39238.1962	P. aeruginosa
GCF_006971785.1	GCF_003571505.1	55236	39239.617	P. aeruginosa
GCF_006971785.1	GCF_000414035.1	55237	39240.3274	P. aeruginosa
GCF_003025345.2	GCF_009911735.1	55239	39241.7482	P. aeruginosa
GCF_003025345.2	GCF_000271985.2	55240	39242.4586	P. aeruginosa
GCF_003025345.2	GCF_012935295.1	55240	39242.4586	P. aeruginosa
GCF_006971785.1	GCF_012935295.1	55240	39242.4586	P. aeruginosa
GCF_006971785.1	GCF_000524595.1	55243	39244.5898	P. aeruginosa
GCF_003025345.2	GCF_016105505.1	55252	39250.9834	P. aeruginosa
GCF_006971785.1	GCF_008033725.1	55253	39251.6938	P. aeruginosa
GCF_003025345.2	GCF_900243355.1_RW109_genomic	55255	39253.1146	P. aeruginosa
GCF_003025345.2	GCF_000414035.1	55258	39255.2458	P. aeruginosa
GCF_003025345.2	GCF_000568855.2	55268	39262.3498	P. aeruginosa
GCF_006971785.1	GCF_016105505.1	55272	39265.1914	P. aeruginosa
GCF_003025345.2	GCF_008033725.1	55273	39265.9018	P. aeruginosa
GCF_006971785.1	GCF_904866275.1_MINF_7A_genomic	55278	39269.4538	P. aeruginosa
GCF_006971785.1	GCF_000568855.2	55280	39270.8746	P. aeruginosa
GCF_006971785.1	GCF_900243355.1_RW109_genomic	55281	39271.585	P. aeruginosa
GCF_003025345.2	GCF_000524595.1	55282	39272.2953	P. aeruginosa
GCF_006971785.1	GCF_001900195.1	55288	39276.5577	P. aeruginosa
GCF_006971785.1	GCF_000026645.1	55290	39277.9785	P. aeruginosa
GCF_003025345.2	GCF_904866275.1_MINF_7A_genomic	55294	39280.8201	P. aeruginosa
GCF_003025345.2	GCF_001900195.1	55297	39282.9513	P. aeruginosa
GCF_003025345.2	GCF_000026645.1	55297	39282.9513	P. aeruginosa
GCF_006971785.1	GCF_001879525.1	55299	39284.3721	P. aeruginosa
GCF_006971785.1	GCF_001606045.1	55302	39286.5033	P. aeruginosa
GCF_006971785.1	GCF_003319235.1	55310	39292.1865	P. aeruginosa
GCF_006971785.1	GCF_001750705.1	55313	39294.3177	P. aeruginosa

GCF_003025345.2	GCF_001606045.1	55315	39295.7385	P. aeruginosa
GCF_006971785.1	GCF_900636735.1_43941_C01_genomic	55321	39300.0009	P. aeruginosa
GCF_003025345.2	GCF_001879525.1	55329	39305.6841	P. aeruginosa
GCF_000017205.1	GCF_017900915.1	55330	39306.3945	P. aeruginosa
GCF_003025345.2	GCF_003319235.1	55330	39306.3945	P. aeruginosa
GCF_003025345.2	GCF_001750705.1	55333	39308.5257	P. aeruginosa
GCF_003025345.2	GCF_900636735.1_43941_C01_genomic	55352	39322.0233	P. aeruginosa
GCF_016743035.1	GCF_017900915.1	55911	39719.1365	P. aeruginosa
GCF_006971785.1	GCF_017900915.1	56059	39824.2756	P. aeruginosa
GCF_003025345.2	GCF_017900915.1	56128	39873.2932	P. aeruginosa
GCF_014490035.1	GCF_009363015.1	58288	82094.1559	A. xylosoxidans/A. insolitus
GCF_000165835.1	GCF_009363015.1	58914	82975.8286	A. xylosoxidans/A. insolitus
GCF_014490035.1	GCF_904423855.1_G1433_genomic	59223	83411.0313	A. xylosoxidans/A. insolitus
GCF_003855615.1	GCF_011149675.1	59667	25302.5582	P. mirabilis
GCF_003855615.1	GCF_018972025.1	59708	25319.9448	P. mirabilis
GCF_003855615.1	GCF_000069965.1	59783	25351.7495	P. mirabilis
GCF_003855615.1	GCF_011045575.1	59802	25359.8067	P. mirabilis
GCF_000165835.1	GCF_904423855.1_G1433_genomic	59805	84230.7335	A. xylosoxidans/A. insolitus
GCF_003855615.1	GCF_000783575.2	59821	25367.8639	P. mirabilis
GCF_003855615.1	GCF_000444425.1	59864	25386.0986	P. mirabilis
GCF_003855615.1	GCF_011383025.1	59875	25390.7633	P. mirabilis
GCF_003855615.1	GCF_013343255.1	59890	25397.1243	P. mirabilis
GCF_003855615.1	GCF_001640985.1	59901	25401.789	P. mirabilis
GCF_003855615.1	GCF_008195605.1	59904	25403.0611	P. mirabilis
GCF_003855615.1	GCF_002310875.1	59905	25403.4852	P. mirabilis
GCF_003855615.1	GCF_010442675.1	59907	25404.3333	P. mirabilis
GCF_003855615.1	GCF_014931585.1	59959	25426.3846	P. mirabilis
GCF_003855615.1	GCF_014843115.1	59961	25427.2327	P. mirabilis
GCF_003855615.1	GCF_019443785.1	59967	25429.7771	P. mirabilis
GCF_003855615.1	GCF_008041895.1	59967	25429.7771	P. mirabilis
GCF_003855615.1	GCF_002055685.1	59978	25434.4418	P. mirabilis
GCF_003855615.1	GCF_018138945.1	59991	25439.9546	P. mirabilis
GCF_003855615.1	GCF_003073935.1	60002	25444.6193	P. mirabilis
GCF_003855615.1	GCF_019192645.1	60005	25445.8915	P. mirabilis
GCF_003855615.1	GCF_008630655.1	60037	25459.4615	P. mirabilis
GCF_003855615.1	GCF_009429045.2	60050	25464.9743	P. mirabilis
GCF_003855615.1	GCF_018336495.1	60098	25485.3293	P. mirabilis
GCF_003855615.1	GCF_002945235.1	60122	25495.5068	P. mirabilis
GCF_003855615.1	GCF_003030945.1	60278	25561.6606	P. mirabilis
GCF_001457475.1_NCTC10807_genomic	GCF_009363015.1	60295	84920.8607	A. xylosoxidans/A. insolitus
GCF_000508285.1	GCF_009363015.1	60333	84974.3808	A. xylosoxidans/A. insolitus
GCF_008432465.1	GCF_009363015.1	60356	85006.7745	A. xylosoxidans/A. insolitus
GCF_900009115.1_BN2877_genomic	GCF_009363015.1	60366	85020.8587	A. xylosoxidans/A. insolitus
GCF_003293535.1	GCF_001457475.1_NCTC10807_genomic	60425	85103.9557	A. xylosoxidans/A. insolitus
GCF_003293535.1	GCF_008432465.1	60446	85133.5326	A. xylosoxidans/A. insolitus
GCF_001639685.1	GCF_001457475.1_NCTC10807_genomic	60448	85136.3494	A. xylosoxidans/A. insolitus
GCF_013282235.1	GCF_009363015.1	60457	85149.0252	A. xylosoxidans/A. insolitus
GCF_001051055.1	GCF_009363015.1	60462	85156.0673	A. xylosoxidans/A. insolitus
GCF_003293535.1	GCF_000508285.1	60466	85161.701	A. xylosoxidans/A. insolitus
GCF_001639685.1	GCF_008432465.1	60468	85164.5179	A. xylosoxidans/A. insolitus
GCF_008245125.1	GCF_001457475.1_NCTC10807_genomic	60472	85170.1516	A. xylosoxidans/A. insolitus
GCF_001639685.1	GCF_000508285.1	60489	85194.0948	A. xylosoxidans/A. insolitus
GCF_003293535.1	GCF_900009115.1_BN2877_genomic	60500	85209.5874	A. xylosoxidans/A. insolitus
GCF_022325125.1	GCF_001457475.1_NCTC10807_genomic	60507	85219.4464	A. xylosoxidans/A. insolitus
GCF_001971645.1	GCF_001457475.1_NCTC10807_genomic	60510	85223.6716	A. xylosoxidans/A. insolitus
GCF_002209555.1	GCF_001457475.1_NCTC10807_genomic	60510	85223.6716	A. xylosoxidans/A. insolitus
GCF_001641515.1	GCF_001457475.1_NCTC10807_genomic	60510	85223.6716	A. xylosoxidans/A. insolitus
GCF_000967095.2_A.x.NH44784-1996_genomic	GCF_009363015.1	60511	85225.0801	A. xylosoxidans/A. insolitus
GCF_001639685.1	GCF_900009115.1_BN2877_genomic	60513	85227.8969	A. xylosoxidans/A. insolitus
GCF_008245125.1	GCF_000508285.1	60513	85227.8969	A. xylosoxidans/A. insolitus
GCF_011063385.1	GCF_001457475.1_NCTC10807_genomic	60515	85230.7138	A. xylosoxidans/A. insolitus
GCF_008245125.1	GCF_008432465.1	60515	85230.7138	A. xylosoxidans/A. insolitus
GCF_003855615.1	GCF_003194305.1	60516	25662.5876	P. mirabilis
GCF_022325125.1	GCF_008432465.1	60519	85236.3475	A. xylosoxidans/A. insolitus
GCF_000783435.2	GCF_001457475.1_NCTC10807_genomic	60525	85244.798	A. xylosoxidans/A. insolitus
GCF_011063385.1	GCF_008432465.1	60534	85257.4738	A. xylosoxidans/A. insolitus
GCF_002209555.1	GCF_008432465.1	60536	85260.2906	A. xylosoxidans/A. insolitus
GCF_001971645.1	GCF_008432465.1	60537	85261.6991	A. xylosoxidans/A. insolitus
GCF_001641515.1	GCF_008432465.1	60539	85264.5159	A. xylosoxidans/A. insolitus
GCF_902860205.1_LMG_6001_genomic	GCF_001457475.1_NCTC10807_genomic	60540	85265.9243	A. xylosoxidans/A. insolitus
GCF_022325125.1	GCF_000508285.1	60548	85277.1917	A. xylosoxidans/A. insolitus
GCF_003293535.1	GCF_013282235.1	60550	85280.0086	A. xylosoxidans/A. insolitus
GCF_000783435.2	GCF_008432465.1	60550	85280.0086	A. xylosoxidans/A. insolitus
GCF_001971645.1	GCF_000508285.1	60551	85281.417	A. xylosoxidans/A. insolitus
GCF_002209555.1	GCF_000508285.1	60551	85281.417	A. xylosoxidans/A. insolitus
GCF_001641515.1	GCF_000508285.1	60551	85281.417	A. xylosoxidans/A. insolitus
GCF_008245125.1	GCF_900009115.1_BN2877_genomic	60556	85288.4591	A. xylosoxidans/A. insolitus
GCF_011063385.1	GCF_000508285.1	60556	85288.4591	A. xylosoxidans/A. insolitus
GCF_022325125.1	GCF_900009115.1_BN2877_genomic	60559	85292.6844	A. xylosoxidans/A. insolitus
GCF_002209555.1	GCF_900009115.1_BN2877_genomic	60563	85298.3181	A. xylosoxidans/A. insolitus
GCF_001641515.1	GCF_900009115.1_BN2877_genomic	60565	85301.1349	A. xylosoxidans/A. insolitus
GCF_000783435.2	GCF_000508285.1	60566	85302.5433	A. xylosoxidans/A. insolitus
GCF_902860205.1_LMG_6001_genomic	GCF_008432465.1	60566	85302.5433	A. xylosoxidans/A. insolitus
GCF_001971645.1	GCF_900009115.1_BN2877_genomic	60568	85305.3602	A. xylosoxidans/A. insolitus
GCF_001639685.1	GCF_013282235.1	60572	85310.9939	A. xylosoxidans/A. insolitus
GCF_011063385.1	GCF_900009115.1_BN2877_genomic	60576	85316.6276	A. xylosoxidans/A. insolitus
GCF_902860205.1_LMG_6001_genomic	GCF_000508285.1	60581	85323.6697	A. xylosoxidans/A. insolitus
GCF_000783435.2	GCF_900009115.1_BN2877_genomic	60585	85329.3034	A. xylosoxidans/A. insolitus
GCF_902860205.1_LMG_6001_genomic	GCF_900009115.1_BN2877_genomic	60599	85349.0213	A. xylosoxidans/A. insolitus
GCF_008245125.1	GCF_013282235.1	60611	85365.9224	A. xylosoxidans/A. insolitus
GCF_022325125.1	GCF_013282235.1	60617	85374.3729	A. xylosoxidans/A. insolitus
GCF_002209555.1	GCF_013282235.1	60622	85381.415	A. xylosoxidans/A. insolitus
GCF_001971645.1	GCF_013282235.1	60627	85388.4571	A. xylosoxidans/A. insolitus
GCF_011063385.1	GCF_013282235.1	60635	85399.7245	A. xylosoxidans/A. insolitus

GCF_001641515.1	GCF_013282235.1	60637	85402.5414	A. xylosoxidans/A. insolitus
GCF_000783435.2	GCF_013282235.1	60660	85434.9351	A. xylosoxidans/A. insolitus
GCF_902860205.1_LMG_6001_genomic	GCF_013282235.1	60675	85456.0614	A. xylosoxidans/A. insolitus
GCF_003293535.1	GCF_000967095.2_A.x.NH44784-1996_genomic	60702	85494.0888	A. xylosoxidans/A. insolitus
GCF_001639685.1	GCF_000967095.2_A.x.NH44784-1996_genomic	60741	85549.0173	A. xylosoxidans/A. insolitus
GCF_008245125.1	GCF_000967095.2_A.x.NH44784-1996_genomic	60783	85608.1711	A. xylosoxidans/A. insolitus
GCF_002209555.1	GCF_000967095.2_A.x.NH44784-1996_genomic	60784	85609.5795	A. xylosoxidans/A. insolitus
GCF_001641515.1	GCF_000967095.2_A.x.NH44784-1996_genomic	60784	85609.5795	A. xylosoxidans/A. insolitus
GCF_022325125.1	GCF_000967095.2_A.x.NH44784-1996_genomic	60784	85609.5795	A. xylosoxidans/A. insolitus
GCF_001971645.1	GCF_000967095.2_A.x.NH44784-1996_genomic	60788	85615.2132	A. xylosoxidans/A. insolitus
GCF_011063385.1	GCF_000967095.2_A.x.NH44784-1996_genomic	60808	85643.3817	A. xylosoxidans/A. insolitus
GCF_000783435.2	GCF_000967095.2_A.x.NH44784-1996_genomic	60810	85646.1985	A. xylosoxidans/A. insolitus
GCF_902860205.1_LMG_6001_genomic	GCF_000967095.2_A.x.NH44784-1996_genomic	60817	85656.0575	A. xylosoxidans/A. insolitus
GCF_003293535.1	GCF_001051055.1	60840	85688.4512	A. xylosoxidans/A. insolitus
GCF_001639685.1	GCF_001051055.1	60851	85703.9439	A. xylosoxidans/A. insolitus
GCF_008245125.1	GCF_001051055.1	60896	85767.3229	A. xylosoxidans/A. insolitus
GCF_022325125.1	GCF_001051055.1	60912	85789.8577	A. xylosoxidans/A. insolitus
GCF_011063385.1	GCF_001051055.1	60919	85799.7166	A. xylosoxidans/A. insolitus
GCF_001641515.1	GCF_001051055.1	60928	85812.3924	A. xylosoxidans/A. insolitus
GCF_902860205.1_LMG_6001_genomic	GCF_001051055.1	60940	85829.2935	A. xylosoxidans/A. insolitus
GCF_002209555.1	GCF_001051055.1	60951	85844.7862	A. xylosoxidans/A. insolitus
GCF_001971645.1	GCF_001051055.1	60957	85853.2367	A. xylosoxidans/A. insolitus
GCF_000783435.2	GCF_001051055.1	60961	85858.8704	A. xylosoxidans/A. insolitus
GCF_001457475.1_NCTC10807_genomic	GCF_904423855.1_G1433_genomic	61501	86619.4188	A. xylosoxidans/A. insolitus
GCF_008432465.1	GCF_904423855.1_G1433_genomic	61510	86632.0946	A. xylosoxidans/A. insolitus
GCF_000508285.1	GCF_904423855.1_G1433_genomic	61540	86674.3473	A. xylosoxidans/A. insolitus
GCF_900009115.1_BN2877_genomic	GCF_904423855.1_G1433_genomic	61588	86741.9516	A. xylosoxidans/A. insolitus
GCF_000967095.2_A.x.NH44784-1996_genomic	GCF_904423855.1_G1433_genomic	61653	86833.4991	A. xylosoxidans/A. insolitus
GCF_013282235.1	GCF_904423855.1_G1433_genomic	61667	86853.217	A. xylosoxidans/A. insolitus
GCF_001051055.1	GCF_904423855.1_G1433_genomic	61676	86865.8928	A. xylosoxidans/A. insolitus
GCF_003293535.1	GCF_009363015.1	64474	90806.6602	A. xylosoxidans/A. insolitus
GCF_001639685.1	GCF_009363015.1	64495	90836.237	A. xylosoxidans/A. insolitus
GCF_022325125.1	GCF_009363015.1	64546	90908.0666	A. xylosoxidans/A. insolitus
GCF_008245125.1	GCF_009363015.1	64555	90920.7424	A. xylosoxidans/A. insolitus
GCF_902860205.1_LMG_6001_genomic	GCF_009363015.1	64570	90941.8688	A. xylosoxidans/A. insolitus
GCF_011063385.1	GCF_009363015.1	64574	90947.5024	A. xylosoxidans/A. insolitus
GCF_002209555.1	GCF_009363015.1	64595	90977.0793	A. xylosoxidans/A. insolitus
GCF_001971645.1	GCF_009363015.1	64597	90979.8962	A. xylosoxidans/A. insolitus
GCF_000783435.2	GCF_009363015.1	64604	90989.7551	A. xylosoxidans/A. insolitus
GCF_001641515.1	GCF_009363015.1	64616	91006.6562	A. xylosoxidans/A. insolitus
GCF_003293535.1	GCF_904423855.1_G1433_genomic	65450	92181.2809	A. xylosoxidans/A. insolitus
GCF_001639685.1	GCF_904423855.1_G1433_genomic	65459	92193.9567	A. xylosoxidans/A. insolitus
GCF_022325125.1	GCF_904423855.1_G1433_genomic	65530	92293.9548	A. xylosoxidans/A. insolitus
GCF_902860205.1_LMG_6001_genomic	GCF_904423855.1_G1433_genomic	65535	92300.9969	A. xylosoxidans/A. insolitus
GCF_001641515.1	GCF_904423855.1_G1433_genomic	65537	92303.8137	A. xylosoxidans/A. insolitus
GCF_011063385.1	GCF_904423855.1_G1433_genomic	65557	92331.9822	A. xylosoxidans/A. insolitus
GCF_008245125.1	GCF_904423855.1_G1433_genomic	65563	92340.4327	A. xylosoxidans/A. insolitus
GCF_000783435.2	GCF_904423855.1_G1433_genomic	65565	92343.2496	A. xylosoxidans/A. insolitus
GCF_002209555.1	GCF_904423855.1_G1433_genomic	65584	92370.0096	A. xylosoxidans/A. insolitus
GCF_001971645.1	GCF_904423855.1_G1433_genomic	65586	92372.8265	A. xylosoxidans/A. insolitus
