# Supplementary material for: Tracking gut microbiome and bloodstream infection in critically ill adults
Source: PLoS One. 2023 Oct 10;18(10):e0289923. doi: 10.1371/journal.pone.0289923 (PMC10564172; doi:10.1371/journal.pone.0289923)
Supplement: S6 Table — (PDF) [file pone.0289923.s007.pdf]

Table S6: SNV/Mbp analysis of unrelated reference and study genomes per species

Organism	Number of genomes compared	Median SNV/Mbp	Mean SNV/Mbp	Empiric 99% threshold
A. xylosoxidans	10	75018	47667	>100
A. insolitus	11	4060	20229	>100
E. coli	28	15095	13094	>100
E. faecalis	15	6574	6401	72
K. pneumoniae	13	4109	3985	>100
P. aeruginosa	49	3644	9756	>100
P. mirabilis	27	5386	7054	>100

Empiric 99% threshold calculated by finding cut off SNV/Mbp value encompassing less than 1% of comparisons between each unrelated genomes or if >100 SNV/Mbp, 100 SNV/Mbp

Because Achromobacter was only identified by blood culture at the genus level, both A. xylosxidans and A. insolitus were used for this analysis

Achromobacter xylosoxidans included due to low numbers of unrelated A. insolitus genomes
