# Supplementary material for: Tracking gut microbiome and bloodstream infection in critically ill adults
Source: PLoS One. 2023 Oct 10;18(10):e0289923. doi: 10.1371/journal.pone.0289923 (PMC10564172; doi:10.1371/journal.pone.0289923)
Supplement: S1 File — (PDF) [file pone.0289923.s008.pdf]

Tracking gut microbiome and bloodstream infection in critically ill adults

Christopher H. Gu, Layla A. Khatib, Ayannah S. Fitzgerald, Jevon Graham-Wooten, Caroline A. Ittner, Scott Sherrill-Mix, YuChung Chuang, Laurel J. Glaser, Nuala J. Meyer, Frederic D. Bushman, Ronald G. Collman

Supplemental Methods

Subjects and specimens.

This was a prospective cohort study of patients ≥18 years old admitted to the medical ICU of the Hospital of the University of Pennsylvania. Patients were eligible for enrollment if they were expected to remain in the ICU for at least 72 hours based on clinician judgement or had a clinical diagnosis of sepsis or in some cases, confirmed blood culture, and if they or a surrogate were able to provide timely informed consent. Patients were enrolled in a larger cohort during several discontinuous periods beginning in 2015 and finishing in 2021 that were determined based on staffing availability (and with a significant gap due to the COVID-19 pandemic), and 139 individuals for whom stool was available were included in this study.

Ethics statement: All patients or their surrogates provided written or verbal informed consent under protocol #823392 approved by the University of Pennsylvania IRB. Verbal consent was witnessed by a member of the medical staff. The authors had access to information that could identify individual participants during and after data collection.

Clinical data were extracted from the electronic medical record by both automatic extraction and manual review. The clinical records were manually reviewed by a physician to generate the "Encounter Diagnosis" column in Table S1. HSCT was defined broadly as either allogeneic or autologous stem cell transplant at any time prior to BSI. Cytotoxic chemotherapy was defined as treatment within 1 month prior to BSI. Neutropenia (<1000/uL) was at the time of BSI. Immunosuppressive therapy was defined as active treatment with immunosuppressive biologics, corticosteroids (≥20 mg/dL prednisone equivalent for ≥1 week prior to BSI), tacrolimus, mycophenolate, azathioprine or similar agents. The "Major Diagnosis" in Table 1 was determined based on automatic extraction of ICD9 or ICD10 codes and verified by manual

review.

Stool specimens were obtained by nursing staff from spontaneous movements or from fecal management system containers, refrigerated immediately upon collection, and stored at -80°C until analysis.

Blood cultures were obtained on patients based on clinical indications, cultured as per routine laboratory procedure, and isolates were frozen at -80°C until sequenced. BSI isolate were identified at the species level, except for one that was only identified at the genus level as *Achromobacter*, and analysis was therefore carried out for that BSI at the genus level as described in Results[1, 2]. Acquisition of blood cultures isolates depended on availability and accessibility of blood cultures in the clinical microbiology lab.

Healthy controls were random sequenced stool samples downloaded from the Human Microbiome Project. The accession numbers can be found in Table S2.

Metagenomic and WGS Sequencing

DNA was extracted from stool samples using the DNeasy PowerSoil Kit (Qiagen, Catalog No. 12888-100) following standard protocol. DNA concentration was measured using Qubit Flurometric Quantitation (Life Technologies) or Quant-iT PicoGreen dsDNA Assay (ThermoFisher). Libraries for Illumina sequencing were prepared using the Nextera XT DNA Library Preparation Kit (Illumina, Catalog No. FC-131-1096) with DT® for Illumina Nextera DNA Unique Dual Indexes (Illumina, 20027213, 20027214, 20027215, 20027216) following the standard protocol. Library concentration was assessed by Qubit Fluorometric Quantitation or Quant-iT™ PicoGreen™ dsDNA Assay and quality and size distributions were analyzed with the TapeStation system (Agilent). Sequencing was performed on the NovaSeq6000 using 2x150bp chemistry or the HiSeq2500 using 2x125bp chemistry.

Blood culture isolates were subcultured from the clinical microbiology laboratory on blood agar (TSA w/ 5% sheep blood) plates (ThermoFisher, R01198). Colonies were picked and inoculated into 5mL Blood Heart Infusion Broth (BD, 211059) to grow overnight at 37°C. A 1 mL aliquot was taken from the inoculated culture and used to make a glycerol stock; the rest was pelleted for storage at -20°C. DNA was extracted from the BC isolate pellet using the DNeasy PowerSoil Kit as mentioned previously. Libraries were prepared using the Illumina DNA Prep kit (Illumina, Catalog No. 20018704) with the DT® for Illumina Nextera DNA Unique Dual

Indexes following the standard protocol. All DNA measurement steps were done with Qubit Fluorometric Quantitation. Libraries were quality and size checked using the Agilent TapeStation system. Sequencing was performed on the MiSeq platform with 2x250bp or 2x300bp chemistry.

Metagenomic sequence processing and BSI whole genome assembly

Stool samples were demultiplexed using DNAbc [3]. Sequencing reads were processed and taxonomically classified using the Sunbeam pipeline v3.0.0 [4]. Processing included adaptor trimming and filtering of low quality, low complexity, and human reads. Reads were classified by Kraken 2 pipeline using the PlusPF database which includes genomes from archaea, bacteria, virus, protozoa, and fungi [5, 6]. Classification of reads went down to the species level. Proportions were calculated by tallying the minimum rank classification limited to species over total reads in a sample. Analysis was performed in R version 3.4.0 [7]. R packages used in the analysis were table1 version 1.3, vegan version 2.5-7, tidyverse version 1.3.1, readxl version 1.4.0, xlsx version 0.6.5.9000, lubridate version 1.8.0, eclectic version 0.1.3, dendextend version 1.15.2, ape version 5.5, tabula version 1.6.1, ggpubr version 0.4.0, ggbeeswam version 0.6.0, ggtree version 1.10.5, biostrings version 2.46.0, nptest version 1.0-3, stringr version 1.4.0. Composition was assessed by Bray-Curtis dissimilarity index with a principal coordinate analysis and stacked bar plot (phylum level), diversity by Shannon index, and dominance by Berger-Parker Index. Statistical testing included PERMANOVA or Wilcoxon rank-sum as indicated.

WGS sequences were demultiplexed through bcl2fastq v2.19.0.316. Sequences were processed using the Sunbeam pipeline using the standard options. The processed sequencing reads were assembled using Unicycler v0.4.7 with the Illumina-only assembly method [8] via an inhouse snakemake pipeline called nanoflow [9]. Genomes were quality checked using the program checkM [10] and pangenome carried out using an inhouse snakemake pipeline named CoreSNPs [11, 12]. CoreSNPs uses Prokka v1.14.5 [13] for genome annotation and Roary v3.12.0 [14] to generate hierarchical clusters based on single nucleotide variants in core genes (genes present in all samples) and the presence and absence of accessory genes along with SamTools v0.1.19-96b5f2294a [15] and SNP-sites v2.4.1 [16] for extraction of core genes and SNVs to compare isolates. Command line options for specific programs can be found within the

pipeline snakemake code. For non-bacterial genomes, blastn was used to determine identity of the genome [17].

Gut microbiome/BSI analysis

Gut metagenomic sequence data was examined for presence of the organism responsible for the BSI. To summarize, the genus and species of the BSI isolate was searched for in the classified sequencing reads. Blood cultures were considered if relevant if stool samples were present 40 days prior or after based on previous literature [18] and to be inclusive. Correct hits were considered only if both genus and species were correctly found, or if the BSI had no indication of species, the correct genus with any species.

Associations between detection of BSI species and underlying conditions (Table 3) were calculated as individual univariate logistic regression models with underlying condition as a dependent binary variable and the proportion stool match as a binary independent variable in R using glm. The reference group for each model was <0.01% stool match group.

Reads from the stool samples were mapped to the blood culture genomes and reference genomes from the same species randomly collected from NCBI. First, the contigs of the BSI draft genome were concatenated together to create single contig genomes, replacing gaps between contigs with 100 N characters. Next, we used a modified version of hisss [19, 20], a HIgh-throughput Shotgun Sequence Searcher. In summary, hisss uses a snakemake workflow to download and align reads to target genomes using grabseqs and bowtie2 with the --very-sensitive-local option, respectively [21, 22]. The modified version includes bcftools v1.8 [23] to extract variants calls from mapped reads against the genomes. The variant calls were considered if they had >7 reads covering the position, >30 PHRED quality score, and >14 mapping quality score. For each comparison, variant calls were summarized to gather total reference bases and SNP bases (bases different than the reference). The proportion of genome aligned was calculated to as (REF + SNP)/(ref genome size (bp)). SNV/Mbp was calculated as (*SNP / (proportion genome aligned))*(1000000 base pair / 1 mega base pair). Alignments between stool and WGS genomes included the cognate BSI WGS (“subject”), the WGS of other subjects’ BSI organisms of the same species if any within the study (“cohort”), and unrelated species-matched WGS downloaded from Genbank (“database”).

To establish a threshold, we first calculated within-species genome differences of the species for which we had WGS. We performed pangenome analysis through coreSNPs to align and gather SNVs between core genes within each genome. These alignments were calculated using unrelated same-species genomes from different subjects in our study (Table S3), and WGS downloaded from Genbank (Table S4), which we limited to the first listed sequence per project to try to ensure that only unrelated genomes were included. Twenty out of 2296 pair-wise within-species comparisons were <100 SNP/Mbp (0.9%). Manual review revealed that 5 reflected comparisons between lab-derived strains of a reference strain, three of them reflected comparisons between the same strains sequenced independently, 3 reflected comparisons between strains submitted from the same institutions, while 9 comparisons (2 clusters of 3 strains and 1 cluster of a strain similar to 2 reference strains, and 1 cluster of 2 strains) had no obvious connection (Table S5). We also estimated what differences might result simply from sequencing error, based on data we previously reported on re-sequencing a single Vibrio campbellii genome 39 times . Comparisons were made between each genome within a species with each comparison contributing to the threshold. For example, a set of 10 genomes would have 50 unique comparisons, including comparisons to itself, leaving the total unique combination to be 40. Therefore, for each species we used the SNV/Mbp threshold that excludes 99% of unrelated genomes to call a match between stool reads and BSI WGS, or 100 SNV/Mbp if it exceeded that level (Table S6).

References

1. Isler B, Kidd TJ, Stewart AG, Harris P, Paterson DL. Achromobacter Infections and Treatment Options. Antimicrob Agents Chemother. 2020;64(11). Epub 2020/08/21. doi: 10.1128/aac.01025-20. PubMed PMID: 32816734; PubMed Central PMCID: PMCPMC7577122.

2. Gomila M, Prince-Manzano C, Svensson-Stadler L, Busquets A, Erhard M, Martínez DL, et al. Genotypic and phenotypic applications for the differentiation and species-level identification of achromobacter for clinical diagnoses. PLoS One. 2014;9(12):e114356. Epub

2014/12/05. doi: 10.1371/journal.pone.0114356. PubMed PMID: 25474264; PubMed Central PMCID: PMCPMC4256396.

3. PennCHOP Microbiome Program. https://github.com/PennChopMicrobiomeProgram/dnabc/.

4. Clarke EL, Taylor LJ, Zhao C, Connell A, Lee JJ, Fett B, et al. Sunbeam: an extensible pipeline for analyzing metagenomic sequencing experiments. Microbiome. 2019;7(1):46. Epub 2019/03/25. doi: 10.1186/s40168-019-0658-x. PubMed PMID: 30902113; PubMed Central PMCID: PMCPMC6429786.

5. Wood DE, Lu J, Langmead B. Improved metagenomic analysis with Kraken 2. Genome Biol. 2019;20(1):257. Epub 2019/11/30. doi: 10.1186/s13059-019-1891-0. PubMed PMID: 31779668; PubMed Central PMCID: PMCPMC6883579.

6. Langmead B. Available from: https://benlangmead.github.io/aws-indexes/k2.

7. Core R TTeam. The R Project for Statistical Computing. Available from: https://www.r-project.org/.

8. Wick RR, Judd LM, Gorrie CL, Holt KE. Unicycler: Resolving bacterial genome assemblies from short and long sequencing reads. PLoS Comput Biol. 2017;13(6):e1005595. Epub 2017/06/09. doi: 10.1371/journal.pcbi.1005595. PubMed PMID: 28594827; PubMed Central PMCID: PMCPMC5481147.

9. Zhao C. Nanoflow: a NANOpore sequencing data bioinformatics workFLOW. Available from: https://github.com/zhaoc1/nanoflow.

10. Parks DH, Imelfort M, Skennerton CT, Hugenholtz P, Tyson GW. CheckM: assessing the quality of microbial genomes recovered from isolates, single cells, and metagenomes. Genome Res. 2015;25(7):1043-55. Epub 2015/05/16. doi: 10.1101/gr.186072.114. PubMed PMID: 25977477; PubMed Central PMCID: PMCPMC4484387.

11. Gu C, Zhao, C. . coreSNPs. Available from: https://github.com/chrgu/coreSNPs.

12. Mölder F, Jablonski KP, Letcher B, Hall MB, Tomkins-Tinch CH, Sochat V, et al. Sustainable data analysis with Snakemake. F1000Research. 2021;10:33. Epub 2021/05/29. doi: 10.12688/f1000research.29032.2. PubMed PMID: 34035898; PubMed Central PMCID: PMCPMC8114187.

13. Seemann T. Prokka: rapid prokaryotic genome annotation. Bioinformatics. 2014;30(14):2068-9. Epub 2014/03/20. doi: 10.1093/bioinformatics/btu153. PubMed PMID: 24642063.

14. Page AJ, Cummins CA, Hunt M, Wong VK, Reuter S, Holden MT, et al. Roary: rapid large-scale prokaryote pan genome analysis. Bioinformatics. 2015;31(22):3691-3. Epub 2015/07/23. doi: 10.1093/bioinformatics/btv421. PubMed PMID: 26198102; PubMed Central PMCID: PMCPMC4817141.

15. Li H, Handsaker B, Wysoker A, Fennell T, Ruan J, Homer N, et al. The Sequence Alignment/Map format and SAMtools. Bioinformatics. 2009;25(16):2078-9. Epub 2009/06/10. doi: 10.1093/bioinformatics/btp352. PubMed PMID: 19505943; PubMed Central PMCID: PMCPMC2723002.

16. Page AJ, Taylor B, Delaney AJ, Soares J, Seemann T, Keane JA, et al. SNP-sites: rapid efficient extraction of SNPs from multi-FASTA alignments. Microb Genom. 2016;2(4):e000056. Epub 2017/03/30. doi: 10.1099/mgen.0.000056. PubMed PMID: 28348851; PubMed Central PMCID: PMCPMC5320690.

17. Altschul SF, Gish W, Miller W, Myers EW, Lipman DJ. Basic local alignment search tool. J Mol Biol. 1990;215(3):403-10. Epub 1990/10/05. doi: 10.1016/s0022-2836(05)80360-2. PubMed PMID: 2231712.

18. Tamburini FB, Andermann TM, Tkachenko E, Senchyna F, Banaei N, Bhatt AS. Precision identification of diverse bloodstream pathogens in the gut microbiome. Nat Med. 2018;24(12):1809-14. Epub 2018/10/17. doi: 10.1038/s41591-018-0202-8. PubMed PMID: 30323331; PubMed Central PMCID: PMCPMC6289251.

19. Taylor LJ, Gu C. Available from: https://github.com/louiejtaylor/hisss.

20. Keeler EL, Taylor LJ, Abbas A, Collman RG, Bushman FD. Rengasvirus, a Circular Replication-Associated Protein-Encoding Single-Stranded DNA Virus-Related Genome That Is a Common Contaminant in Metagenomic Data. Microbiol Resour Announc. 2021;10(18). Epub 2021/05/08. doi: 10.1128/mra.00273-21. PubMed PMID: 33958399.

21. Taylor LJ, Abbas A, Bushman FD. grabseqs: simple downloading of reads and metadata from multiple next-generation sequencing data repositories. Bioinformatics. 2020;36(11):3607-9. Epub 2020/03/11. doi: 10.1093/bioinformatics/btaa167. PubMed PMID: 32154830; PubMed Central PMCID: PMCPMC7267817.

22. Langmead B, Salzberg SL. Fast gapped-read alignment with Bowtie 2. Nat Methods. 2012;9(4):357-9. Epub 2012/03/06. doi: 10.1038/nmeth.1923. PubMed PMID: 22388286; PubMed Central PMCID: PMCPMC3322381.

23. Danecek P, Bonfield JK, Liddle J, Marshall J, Ohan V, Pollard MO, et al. Twelve years of SAMtools and BCFtools. Gigascience. 2021;10(2). Epub 2021/02/17. doi: 10.1093/gigascience/giab008. PubMed PMID: 33590861; PubMed Central PMCID: PMCPMC7931819.

24. Gu CH, Zhao C, Hofstaedter C, Tebas P, Glaser L, Baldassano R, et al. Investigating hospital Mycobacterium chelonae infection using whole genome sequencing and hybrid assembly. PLoS One. 2020;15(11):e0236533. Epub 2020/11/10. doi: 10.1371/journal.pone.0236533. PubMed PMID: 33166284; PubMed Central PMCID: PMCPMC7652343.
